# Defining the Minimal Factors Required for Erythropoiesis through Direct Lineage Conversion

**DOI:** 10.1016/j.celrep.2016.05.027

**Published:** 2016-06-02

**Authors:** Sandra Capellera-Garcia, Julian Pulecio, Kishori Dhulipala, Kavitha Siva, Violeta Rayon-Estrada, Sofie Singbrant, Mikael N.E. Sommarin, Carl R. Walkley, Shamit Soneji, Göran Karlsson, Ángel Raya, Vijay G. Sankaran, Johan Flygare

**Affiliations:** 1 Department of Molecular Medicine and Gene Therapy, Lund Stem Cell Center, Lund University, 22184 Lund, Sweden; 2 Center of Regenerative Medicine in Barcelona, Barcelona Biomedical Research Park, Doctor Aiguader 88, 08003 Barcelona, Spain; 3 The Rockefeller University, New York, NY 10065, USA; 4 Division of Molecular Hematology, BMC B12, Lund Stem Cell Center, Lund University, 22184 Lund, Sweden; 5 St. Vincent’s Institute of Medical Research and Department of Medicine, St Vincent’s Hospital, University of Melbourne, Fitzroy, VIC 3065, Australia; 6 Catalan Institution for Research and Advanced Studies, 08010 Barcelona, Spain; 7 Biomedical Research Networking Center in Bioengineering, Biomaterials and Nanomedicine (CIBER-BBN), 28029 Madrid, Spain; 8 Division of Hematology/Oncology, Boston Children’s Hospital and Department of Pediatric Oncology, Dana-Farber Cancer Institute, Harvard Medical School, Boston, MA 02115, USA; 9 Broad Institute of the Massachusetts Institute of Technology and Harvard, Cambridge, MA 02142, USA

## Abstract

Erythroid cell commitment and differentiation proceed through activation of a lineage-restricted transcriptional network orchestrated by a group of well characterized genes. However, the minimal set of factors necessary for instructing red blood cell (RBC) development remains undefined. We employed a screen for transcription factors allowing direct lineage reprograming from fibroblasts to induced erythroid progenitors/precursors (iEPs). We show that *Gata1*, *Tal1*, *Lmo2*, and *c-Myc* (GTLM) can rapidly convert murine and human fibroblasts directly to iEPs. The transcriptional signature of murine iEPs resembled mainly that of primitive erythroid progenitors in the yolk sac, whereas addition of *Klf1* or *Myb* to the GTLM cocktail resulted in iEPs with a more adult-type globin expression pattern. Our results demonstrate that direct lineage conversion is a suitable platform for defining and studying the core factors inducing the different waves of erythroid development.

## Highlights

• *Gata1*, *Tal1*, *Lmo2*, and *c-Myc* reprogram fibroblasts to erythroid progenitors (iEPs)
• iEP gene expression is more similar to that of primitive than definitive erythroblasts
• *Klf1* or *Myb* overexpression induces adult hemoglobin expression in iEPs

## Graphical Abstract

**Figure:**
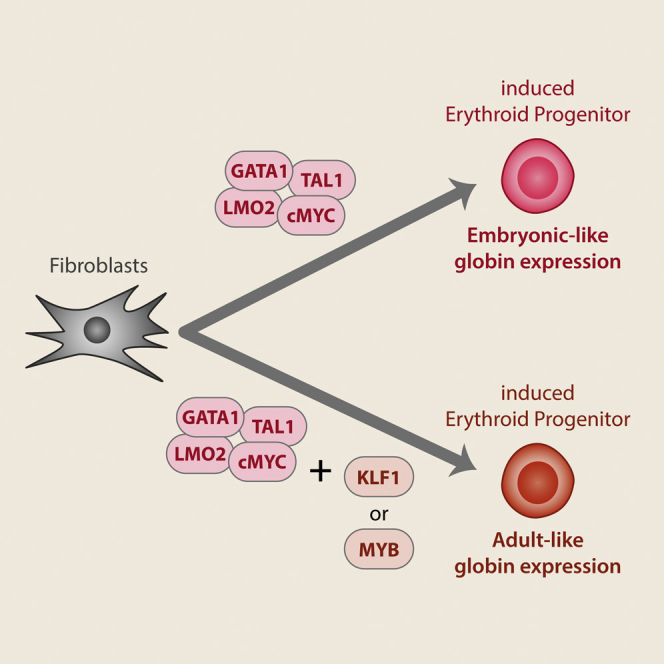

## Teaser

Capellera-Garcia et al. show that *Gata1*, *Tal1*, *Lmo2*, and *c-Myc* directly convert murine and human fibroblasts into erythroid progenitor/precursor cells. This finding defines a conserved transcriptional program instructing erythroid cell fate in mammals.

## Introduction

Although several factors are known to participate in the conserved genetic program instructing development of committed erythroid progenitors, the minimal combination of factors required for direct induction of erythroid cell fate remains unknown. The identification of the key players controlling red blood cell (RBC) development is important for understanding basic biology and can be used to study and recapitulate erythropoiesis in vitro as well as to model and develop new therapies for RBC disorders (Tsiftsoglou et al., 2009). Fate decisions in erythropoiesis have been investigated extensively, focusing on lineage-specific transcription factors and cofactors as the main drivers of the process (Cantor and Orkin, 2002, Shivdasani and Orkin, 1996). Genes found to be essential for normal RBC development in mice include *Gata1*, *Zfpm1*, *Lmo2*, *Klf1*, *Myb*, *Tal1*, *Runx1*, and *Ldb1* (Mead et al., 2001, Palis, 2014). However, the factors constituting the core transcriptional machinery that initiates and specifies erythroid cell fate are still unknown.

A major obstacle for defining core transcriptional networks is the difficulty of discriminating “instructive” factors from “permissive” factors. Numerous studies have demonstrated that it is possible to directly convert a mature cell type into another, bypassing the pluripotent state, using a defined set of lineage-instructive transcription factors (Jopling et al., 2011, Takahashi, 2012). This approach, called direct lineage reprogramming, can yield a wide range of clinically relevant cell types, such as neurons, cardiomyocytes, and hepatocytes (Huang et al., 2011, Ieda et al., 2010, Sekiya and Suzuki, 2011, Vierbuchen et al., 2010). Because the converted cells resemble their bona fide counterparts in terms of phenotype and function, direct lineage reprogramming is currently a widely investigated approach for generating defined cell types for regenerative medicine. In contrast to loss-of-function studies, direct reprogramming distinguishes absolutely essential cell fate-inducing factors from merely permissive factors, revealing the master regulators of specific cell lineages (Vierbuchen and Wernig, 2011). Therefore, we reasoned that direct lineage reprogramming is an unambiguous method for defining the core transcriptional machinery directing RBC development.

Several laboratories have described methods for reprogramming differentiated somatic cells to hematopoietic progenitors with multilineage potential (Batta et al., 2014, Pereira et al., 2013, Riddell et al., 2014, Szabo et al., 2010), whereas others have reported protocols of direct induction to the erythroid lineage starting from B cells (Sadahira et al., 2012) and pluripotent cell sources (Weng and Sheng, 2014). However, none of these studies have shown robust erythroid-restricted fate conversion from non-hematopoietic differentiated somatic cells.

Here we identify the transcription factors *Gata1*, *Tal1*, *Lmo2*, and *c-Myc* (GTLM) as the minimal set of factors for direct conversion of mouse and human fibroblasts into erythroid progenitors. The resulting cells, which we term induced erythroid progenitors/precursors (iEPs), resemble bona fide erythroid cells in terms of morphology, colony-forming capacity, and gene expression. While murine GTLM iEPs express both embryonic and adult globin genes, the addition of *Klf1* or *Myb* induces a switch in globin gene expression to generate iEPs with a predominant definitive-type globin expression pattern. This approach can be used as a model for understanding, controlling, and recapitulating erythroid lineage development and disease.

## Results

### A Combination of Transcription Factors Induces the Erythroid Fate in Murine Fibroblasts

**Figure 1:**
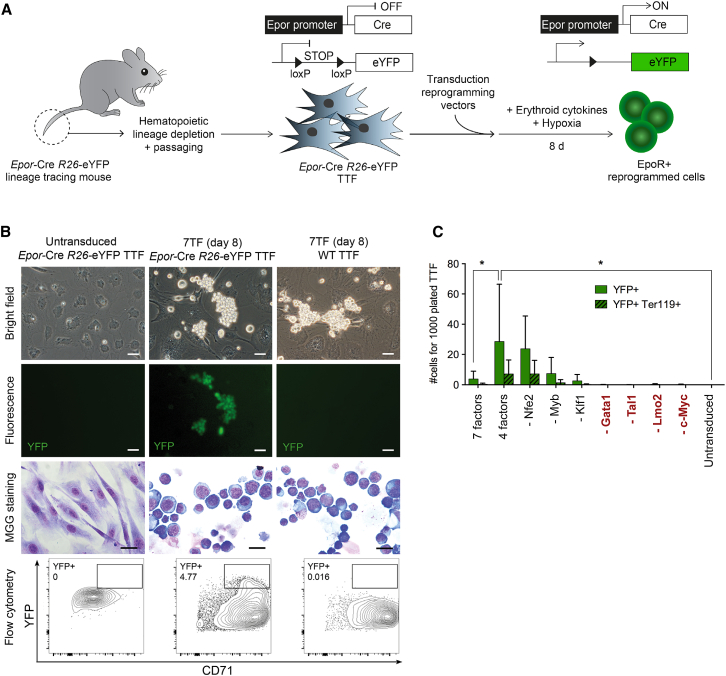
Forced Expression of *Gata1*, *Tal1*, *Lmo2*, and *c-Myc* Reprograms Murine Adult Fibroblasts into Erythroid Progenitors (A) Experimental design for transcription factor-mediated reprogramming of erythroid reporter (*Epor-*Cre *R26*-eYFP) TTFs to EpoR^+^ reprogrammed cells. (B) Representative live-cell, bright-field images, fluorescence images, May-Grünwald-Giemsa staining images, and flow cytometry plots of untransduced TTFs and 7TF-iEPs generated from *Epor-*Cre *R26*-eYFP TTFs and WT TTFs on day 8. Scale bars, 20 μm. (C) Numbers of YFP^+^ and YFP^+^Ter119^+^ total live cells after removal of individual TFs from the 7TF pool on day 8. Data are presented as mean ± SD (n = 2–3). ^∗^p ≤ 0.05. See also Figure S1.

We hypothesized that overexpression of transcription factors involved in hematopoietic and, specifically, erythroid development in fibroblasts could directly convert these cells into erythroid progenitors or precursors. A retroviral library was created from mouse fetal liver (FL) cDNA expressing the coding region of 63 candidate factors (Table S1). Adult tail tip fibroblasts (TTFs) were derived from erythroid lineage-tracing mice (Heinrich et al., 2004), which express the yellow fluorescent protein (eYFP) from the *Rosa26* locus in all cells that have expressed the erythropoietin receptor (*Epor*, Cre knocked into one allele of the endogenous *Epor* locus) transcript at any stage of their development (Figure 1A). In vivo, the expression of eYFP is first detected in bipotent progenitors of megakaryocytes and erythrocytes (pre-MegEs) and is subsequently robustly expressed in erythroid progenitors (Singbrant et al., 2011). Importantly, eYFP was never detected in other hematopoietic lineages or cell types examined. TTF cultures were carefully depleted of hematopoietic cells by magnetic separation using a cocktail of nine hematopoietic antibodies (Experimental Procedures) and passaged at least three times prior to transduction to obtain pure fibroblast cultures. The primary readout for erythroid lineage conversion was the formation of colonies of eYFP^+^ (EpoR^+^) round cells.

Through screening multiple combinations of candidate factors, a combination of seven transcription factors was identified (*Nfe2*, *Myb*, *Klf1*, *Gata1*, *Tal1*, *Lmo2*, and *c-Myc*), that, in 8 days, converted TTFs into clusters of round cells displaying an erythroid precursor-like morphology. These clusters were eYFP^+^ (EpoR^+^), indicating induction of the erythroid transcriptional program (Figure 1B). Hence, we termed these cells iEPs.

### Only Four Factors Are Necessary and Sufficient for iEP Generation

To identify the minimal set of factors required for iEP generation, we performed single factor subtraction experiments. We found that removal of *Gata1*, *Tal1*, *Lmo2*, or c-*Myc* from the factor cocktail completely abrogated iEP formation (Figure 1C; Figure S1). Notably, TTF reprogramming to iEPs was significantly enhanced using only these four factors compared with the initial seven factors (Figure 1C). Thus, we concluded that GTLM factors constitute the minimal set required for iEP generation.

### iEPs Exhibit Morphological and Gene Expression Properties of Erythroid Cells

**Figure 2:**
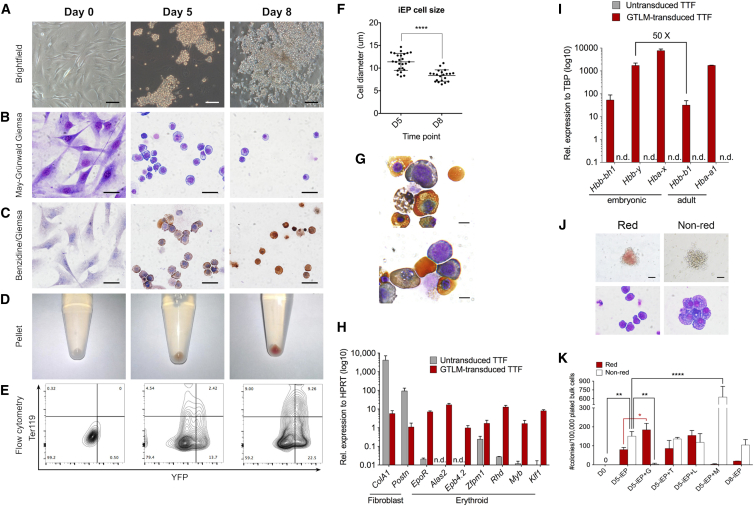
Induced Erythroid Progenitors Exhibit Properties of Bona Fide Erythroid Cells (A–E) Time course of iEP generation of untransduced TTFs (day 0) and bulk GTLM-transduced TTFs on days 5 and 8 (representative of n = 2–3). Transdifferentiation was evaluated by (A) live-cell, bright-field images of single wells (scale bar, 50 μm); (B) May-Grünwald-Giemsa staining cytospin (scale bar, 20 μm); (C) benzidine/Giemsa staining cytospin (scale bar, 20 μm); (D) macroscopic inspection of cell pellets; and (E) representative flow cytometry plots showing YFP/Ter119 expression. (F) Cell diameter of iEPs harvested on days 5 and 8 measured by CellSens Standard 1.6 software from several cytospin slides, showing a decrease in cell size on day 8. Data are presented as mean ± SD (n = 21–25). ^∗∗∗∗^p ≤ 0.0001. (G) Representative high-resolution benzidine/Giemsa cytospin images of GTLM-transduced TTFs on day 8. Scale bar, 5 μm. (H and I) Relative mRNA expression of (H) relevant erythroid and fibroblast-specific genes and (I) globin genes in bulk GTLM-transduced TTFs (red columns) versus untransduced TTF (gray columns) on day 8, determined by qPCR. Data are presented as mean ± SD (n = 4–6 for iEPs, n = 2 for untransduced TTFs). (J) Representative bright-field and May-Grünwald-Giemsa cytospin images of iEP-derived red and non-red colonies. (K) Colony counts generated from plated untransduced TTFs, bulk day 5 iEPs, bulk day 8 iEPs and bulk day 5 iEPs generated by doubling the ratio of each of the GTLM factors. Scale bars, 50 μm (colony images) and 20 μm (cytospin images). Data are presented as mean ± SD (n = 3). ^∗∗^p ≤ 0.001; ^∗∗∗∗^p ≤ 0.0001. See also Figure S2.

To better characterize iEP emergence, we examined reprogramming at different time points: on day 5, when the first YFP^+^ clusters of round cells appeared, and on day 8, when large YFP^+^ colonies could be observed (Figure 2A). On day 5, iEPs displayed an erythroid precursor-like morphology, featuring a characteristic central nucleus, coarse chromatin, and blue cytoplasm after May-Grünwald-Giemsa staining (Figure 2B). Some cells also stained weakly positive with benzidine stain and appeared mildly red when pelleted, indicating that the cells contained hemoglobin (Figures 2C and 2D). On day 5, a small fraction of iEPs co-expressed eYFP and the erythroid-specific surface marker Ter119 (Figure 2E). iEPs harvested on day 8 presented a more differentiated erythroid phenotype. They were significantly smaller, accumulated more hemoglobin, and upregulated Ter119 expression (Figures 2B–2F). Very few enucleated reticulocytes were observed, suggesting inefficient enucleation (Figure 2G). Kinetic analysis by flow cytometry revealed that erythroid precursor output was highest on day 6, followed by day 8, with 10.5% ± 4.6% and 6.6% ± 0.5% of live YFP^+^ cells co-expressing CD71 and Ter119, respectively (Figure S2A). Furthermore, the pan hematopoietic marker CD45, which is downregulated in erythroid cells, was not expressed at any time during reprogramming. This suggests that GTLM reprogramming is direct and does not involve an intermediate hematopoietic progenitor stage.

As expected from the erythroid flow cytometric profile and cell morphology, day 8 iEPs downregulated the expression of fibroblast-specific genes and upregulated the expression of erythroid cell-specific genes (Figure 2H). We then analyzed the expression of the different globin genes from both the α and β globin clusters, which are differentially expressed throughout development. In the mouse, all globin genes of the α (*Hba-x*, *Hba-a1*, and *Hba-a2*) and the β globin loci (*Hbb-y*, *Hbb-bh1*, *Hbb-b1*, and *Hbb-b2*) are expressed in primitive erythroid cells, whereas only adult globin genes (*Hba-a1*, *Hba-a2*, *Hbb-b1*, and *Hbb-b2*) are expressed in definitive erythroid cells (Kingsley et al., 2006). Day 8 iEP expressed both embryonic and adult globins, with predominant expression of the embryonic types (Figure 2I). Specifically for the β-globin locus, iEPs expressed 50 times more embryonic *Hbb-y* than adult *Hbb-b1*, suggesting that, although adult fibroblasts were used, GTLM induces an erythroid program that is more similar to primitive than definitive erythropoiesis.

Next, we assayed the in vitro differentiation capacity of both day 5 and day 8 iEPs by colony-forming assays. After 8 days in methylcellulose supplemented with human Erythropoietin (hEPO), murine stem cell factor (mSCF), and dexamethasone, iEPs formed two types of colonies: distinctly red (red iEP) and not visibly red (non-red iEP) (Figure 2J). Although cells from red iEP colonies displayed erythroblast morphology, cells from non-red colonies were irregular, had a large deep blue and granular cytoplasm, and did not resemble erythroid cells (Figure 2J). Of the day 5 iEPs, approximately 1 in 1,000 formed red colonies, suggesting that most cells reprogrammed to a more differentiated erythroid cell state without colony-forming ability. This ratio was reduced in day 8 iEPs, which could be explained by iEPs undergoing differentiation from days 5–8 and/or the pMX vectors suffering silencing over time (Figure S2B). In parallel, we asked whether different stoichiometric ratios of GTLM factors could improve the iEPs’ erythroid colony output. Interestingly, day 5 iEPs generated with GTLM in a 2:1:1:1 ratio (D5-iEP+G) gave rise to red colonies almost exclusively, whereas day 5 iEPs generated with GTLM in a 1:1:1:2 ratio (D5-iEP+M) generated almost only non-red colonies (Figure 2K). Increasing the ratio of *Tal1* or *Lmo2* had no significant effect. These data imply that optimizing the stoichiometry of the reprogramming factors can further enhance the erythroid output.

In addition to adult tail tip fibroblasts, iEPs could also be generated from murine embryonic fibroblasts (Figures S2C–S2E), demonstrating that the GTLM factors can reprogram fibroblasts from other origins. GTLM induction of the erythroid fate is thus a rapid and direct process, yielding erythroblast-like cells with bona fide properties.

### iEP-Derived Red Colonies Display a Gene Expression Signature Similar to Bona Fide Burst-Forming Unit-Erythroid Colony Cells

**Figure 3:**
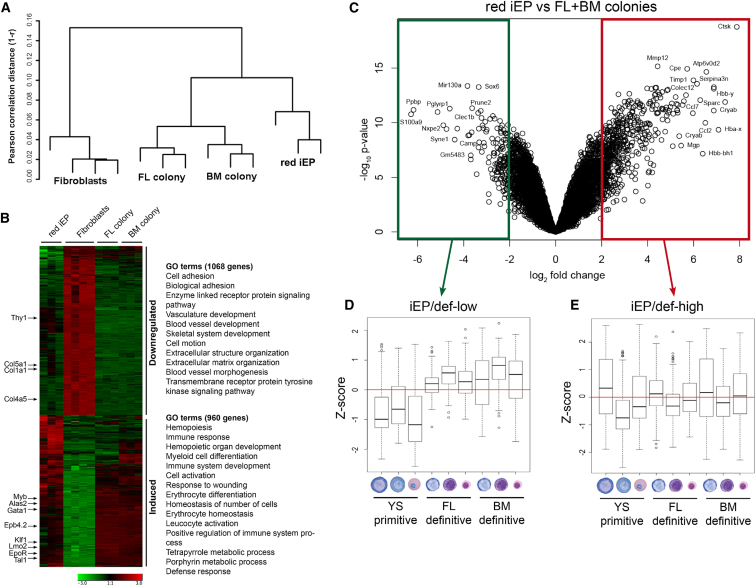
iEP-Derived Red Colonies Retain a Primitive Erythroblast Expression Signature (A) Unsupervised clustering of samples included in microarray analysis: cultured TTFs (fibroblasts), E14.5 fetal liver BFU-Es (FL colony), adult bone marrow BFU-Es (BM colony), and iEP-derived red colonies (red iEP). (B) Heatmap depicting genes with ± 2 log_2_ fold differential expression between red iEPs and fibroblasts. Downregulated and induced gene clusters are highlighted with their most representative GO terms (p ≤ 10^−10^) according to DAVID (Huang et al., 2009a, Huang et al., 2009b). (C) Volcano plot presenting differentially expressed genes between red iEPs and bona fide BFU-Es (FL and BM BFU-Es). The log_2_ fold difference is plotted on the x axis, and the p value-adjusted significance is plotted on the y axis (–log_10_ scale). (D and E) Genes with at least 4-fold higher expression in bona fide BFU-Es than in red iEPs (iEP/def-low) and genes with at least 4-fold higher expression in red iEPs than in bona fide BFU-Es (iEP/def-high) were searched in the publicly available Erythron database (Kingsley et al., 2013). Box plots show the average expression of selected iEP/def-low (D) and iEP/def-high (E) genes in YS primitive, FL-definitive and BM-definitive erythroid cells at different stages of maturation. Pictures were taken with permission from Erythron DB (EMBL-EBI: E-MTAB-1035). See also Figure S3.

To characterize the reprogrammed cells at the molecular level, we performed global gene expression profiling comparing iEPs with bona fide erythroid progenitors and TTFs. To obtain RNA from pure cell populations, burst-forming unit-Erythroid (BFU-E) colony-forming assays were performed on day 5 iEPs as well as on mouse embryonic day (E) 14.5 FL and adult bone marrow (BM) cells. iEP-derived red and non-red colonies were picked separately, and untransduced TTFs were collected as controls. Unsupervised hierarchical clustering revealed that iEP-derived red colonies (red iEPs) clustered together with primary BFU-Es (FL colony and BM colony) (Figure 3A), indicating that their overall transcriptome is more similar to bona fide erythroid progenitors than to their starting fibroblast cell type. Genes differentially expressed more than 2-fold were mined for significantly overrepresented functional categories using the annotation tool DAVID (Database for Annotation, Visualization and Integrated Discovery) (Huang et al., 2009a, Huang et al., 2009b), demonstrating that genes induced in red iEPs were significantly associated with gene ontology (GO) terms relating to hematopoiesis, erythrocyte function, and development (Figure 3B). On the contrary, genes downregulated in red iEPs compared with starting fibroblasts were significantly associated with fibroblast-like function, such as “extracellular matrix organization,” consistent with the vast inactivation of the fibroblast gene expression program. Taken together, these data demonstrate that red iEP-derived colonies show large-scale downregulation of the fibroblast-specific program and extensive activation of genes specific to the erythroid lineage, indicating that GTLM factors are sufficient to trigger global transcriptional modeling toward the erythroid lineage.

### iEP-Derived Non-red Colonies Show Incomplete Reprogramming

To characterize the iEP-derived non-red colonies, we analyzed the differentially expressed genes between red iEPs and non-red iEPs (Figure S3). We found that non-red iEPs lacked induction of genes associated with terminal erythropoiesis, such as globins and genes necessary for heme production (Figure S3A). Moreover, the expression levels of *Gata1*, *Tal1*, and *Lmo2* were 5.6-, 5.2-, and 1.7-fold higher in red iEPs than in non-red iEPs, respectively (Figure S3B). This suggests that non-red iEP colonies have failed to completely reprogram and generate hemoglobinized cells, possibly as a result of inadequate GTL factor expression levels or factor stoichiometry.

### The Gene Expression Profile of iEP-Derived Red Colonies Resembles that of Primitive Erythroblasts

Because red iEPs expressed mainly embryonic globin and did not enucleate efficiently, we asked whether their global gene expression profile resembled primitive rather than definitive erythroid cells. Interestingly, the embryonic globin genes (*Hbb-y*, *Hba-x*, and *Hbb-bh1*) were within the top seven differentially expressed genes between red iEPs and bona fide definitive FL and BM BFU-Es (Figure 3C), confirming that this distinctive feature of the primitive erythroid transcriptional program was active in iEPs compared with definitive erythroid cells. Next, we analyzed genes that were at least 4-fold more expressed in bona fide BFU-Es than in red iEPs (iEP/def-low) and genes that were at least 4-fold more expressed in red iEPs than in bona fide BFU-Es (iEP/def-high) and inspected their behavior in data within the Erythron database, which contains global gene expression profiles from primitive, fetal definitive, and adult definitive erythroid cells in mice (Kingsley et al., 2013). We found that iEP/def-low genes in general had lower expression in primitive erythroid cells from the yolk sac compared with the definitive erythroid cells from FL and BM, suggesting that iEPs retain an expression signature similar to that of primitive erythroblasts (Figure 3D). Regarding the iEP/def-high gene set, we did not observe a correlation with the yolk sac profile, although the three embryonic globin genes were among the top differentially expressed genes in this list (Figure 3E). This suggests that bulk iEPs primarily exhibit a molecular signature that is more comparable with a primitive than definitive erythroid program, possibly because additional factors are necessary to enable definitive erythropoiesis. These findings also raise the question of whether individual iEP clones are different so that some are close to primitive erythroid cells, whereas other clones are more similar to definitive erythroid cells.

### Induction of Adult Globin Expression by *Klf1* and *Myb*

**Figure 4:**
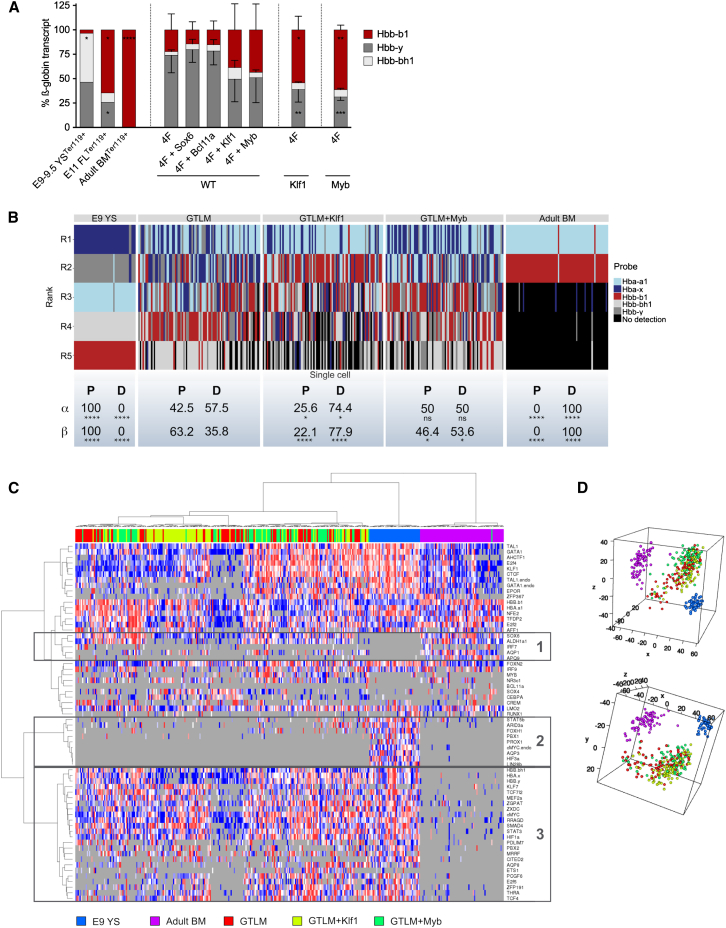
*Klf1* and *Myb* Enhance Adult Hemoglobin Expression in Single iEPs (A) Percentage of embryonic (Hbb-bh1 and Hbb-y) and adult (Hbb-b1) globin transcripts under different test conditions on day 8, determined by qPCR. WT samples denote wild-type TTFs transduced simultaneously with GTLM factors (4F) plus an additional factor. Klf1 and Myb samples denote TTFs with constitutive expression of *Klf1* and *Myb*, respectively, transduced with GTLM factors. Bulk WT GTLM-transduced TTF, Ter119^+^ cells from E9-9.5 yolk sacs, Ter119^+^ cells from E11 fetal livers, and Ter119^+^ cells from adult bone marrow were used as controls. Data are presented as mean ± SD (n = 3–7 for iEP samples, n = 1 for bona fide cells). ^∗^p ≤ 0.05; ^∗∗^p ≤ 0.01; ^∗∗∗^p ≤ 0.001; ^∗∗∗∗^p ≤ 0.0001. (B) Map depicting expression of globin genes in single sorted YFP^+^ Ter119^+^ E9 yolk sac cells, adult bone marrow cells, day 8 GTLM-iEPs, day 8 GTLM^+^Klf1-iEPs, and day 8 GTLM^+^Myb^−^ iEPs. Globin genes are ranked by their iCt values (iCt = 50 − Ct) in each single cell from highest (R1) to lowest (R5) (Figure S4B). Percentages of single cells in each group displaying a “primitive” (P) or definitive (D) globin expression pattern are shown below. P if iCt^Hba-a1^ < iCt^Hba-x^ for the α locus or iCt^Hbb-b1^ < iCt^Hbb-y^ for the β locus; D if iCt^Hba-a1^ > iCt^Hba-x^ for the α locus or iCt^Hbb-b1^ > iCt^Hbb-y^ for the β locus. ^∗^p ≤ 0.05; ^∗∗∗∗^p ≤ 0.0001. (C) Heatmap depicting the expression of 64 genes selected for single-cell qRT-PCR in sorted YFP^+^ Ter119^+^ E9 yolk sac cells (blue), adult bone marrow (purple), day 8 GTLM-iEPs (red), day 8 GTLM^+^Klf1-iEPs (yellow), and day 8 GTLM^+^Myb^−^ iEPs (green). (D) Principal component analysis. Shown are three-dimensional plots of five sample sets shown from two different points of view. Sample color labeling is consistent with Figure 4C. See also Figure S4.

We next asked whether the overexpression of additional factors could increase the expression of adult hemoglobin in iEPs at the expense of the embryonic globins. This would identify key regulators of hemoglobin expression in iEP as well as improve the potential of these cells for therapeutic applications, as highlighted by Trakarnsanga et al. (2014). We evaluated the transcription factors *Sox6*, *Bcl11a*, *Klf1*, and *Myb*, all previously identified to directly or indirectly downregulate the expression of embryonic and fetal globin genes (Sankaran et al., 2009, Sankaran et al., 2011, Yi et al., 2006, Zhou et al., 2010). When overexpressing the GTLM factors (4F) together with a single “switching” factor in wild-type (WT) TTF, only *Klf1* and *Myb* increased the expression ratio of adult *Hbb-b1* over embryonic *Hbb-y* compared with the 4F alone, although differences were not statistically significant (Figure 4A).

To investigate whether constitutive expression of switching factors could reinforce this trend, we cloned *Klf1* and *Myb* independently into retroviral vectors including a blasticidin selection gene and generated TTF cell cultures with constitutive expression of *Klf1* or *Myb* (Klf1 and Myb). iEP generated from these fibroblasts presented a reversed globin expression pattern and predominantly expressed adult *Hbb-b1* globin (54.3% ± 13.9% for Klf1 and 61.3% ± 4.9% for Myb). This expression pattern is similar to that of Ter119^+^ cells harvested from E11 FL, where we still detected *Hbb-y* expression, possibly because of the presence of residual primitive erythroblasts (Figure 4A; McGrath et al., 2011). Thus, the persistence of *Hbb-y* in *Klf1*- and *Myb*-overexpressing cells could reflect the presence of clones with incomplete reprogramming to a definitive-type regulation of globin genes.

Next we investigated the clonal identity and heterogeneity of iEPs and tested whether the addition of *Klf1* and *Myb* generated clones with a more definitive erythroid phenotype overall or merely affected globin expression. We sorted single YFP^+^ Ter119^+^ iEPs that were generated from either *Epor* reporter TTF or *Epor* reporter TTF with constitutive expression of *Klf1* or *Myb* and performed single-cell qRT-PCR (Figure S4A). As for the globin genes, we ranked them from highest (R1) to lowest (R5) based on the inverse Ct value (50 – Ct = iCt) of each gene in each single cell (Figure S4B; Figure 4B). GTLM-iEPs expressed both embryonic and adult globins at the single-cell level, indicating that reprogramming generates iEPs with a mixture of globins (Figure 4B). Consistent with our previous observation, *Klf1* and *Myb* overexpression increased the frequency of single cells with a “definitive” globin expression pattern. Of note, *Klf1* affected the expression of both the α and β loci, whereas *Myb* only modified the β locus (Figure 4B). However, not all *Klf1*- and *Myb*-overexpressing cells displayed a definitive-type regulation of globin genes, denoting clonal heterogeneity in reprogramming and globin loci regulation.

Then we analyzed the expression of primitive-specific and definitive-specific genes in the three iEP subsets. We selected transcriptional regulators and other genes that were previously described to be differentially expressed between primitive and adult erythroblasts, regardless of their differentiation stage (see Table S5 in Kingsley et al., 2013). Unsupervised hierarchical clustering revealed that iEPs clustered together with primitive yolk sac erythroid cells (E9 YS), indicating that, based on the selected genes, iEPs are more similar to primitive than definitive erythroid cells (Figure 4C). The clustering did not change when globin and reprogramming genes were removed from the analysis (data not shown). Closer examination of gene groups revealed that single iEPs expressed some definitive-specific genes, such as *Sox6* and *Aldh1a1* (cluster 1), as well as some primitive-specific genes, such as *Cited2* and *Rragd* (cluster 3). There was also a group of primitive-specific genes, such as *Lin28b* and *Aqp3*, whose expression was clearly suppressed in iEPs (cluster 2). Furthermore, the addition of *Klf1* or *Myb* did not clearly change the expression of primitive- or definitive-specific genes compared with GTLM alone (Figure 4C). In conclusion, single-cell qRT-PCR demonstrates that iEP clones possess an expression profile reflective of a mixture between primitive and definitive erythropoiesis (Figure 4D) and that *Klf1* and *Myb* do not induce a switch from a generally primitive to a definitive gene expression program but, instead, mainly act as globin switching factors in iEPs by increasing adult hemoglobin expression at the expense of embryonic globins.

### Human iEPs Generated by Forced Expression of GTLM

**Figure 5:**
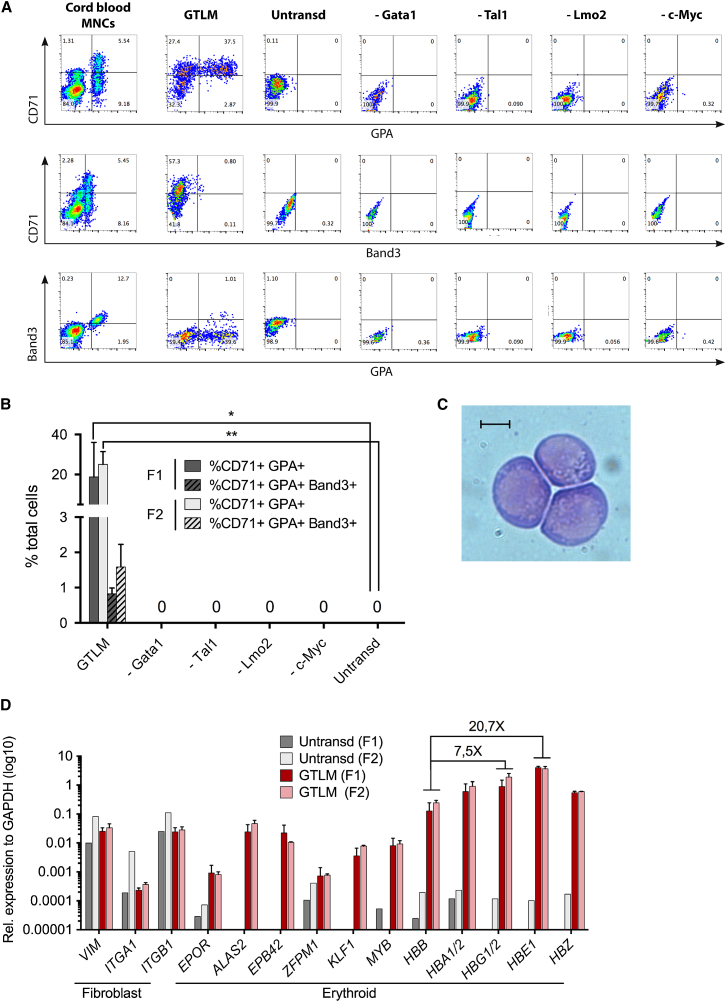
Induction of Erythroid Progenitors from Human Foreskin Fibroblasts by *Gata1*, *Tal1*, *Lmo2*, and *c-Myc* (A) Representative flow cytometry plots showing CD71, Glycophorin A (GlyA), and Band3 expression in bulk GTLM-transduced HFFs on day 12. Cord blood mononuclear cells and factor minus one combinations were used as controls. The plots shown belong to fibroblast line F1 only (n = 2–3). (B) Percentage of CD71^+^GlyA^+^ and CD71^+^GlyA^+^Band3^+^ in total live cells for the different transcription factor combinations on day 12. Data are presented as mean ± SD (n = 2–3) for two different fibroblast lines (F1 and F2). ^∗^p ≤ 0.05; ^∗∗^p ≤ 0.01. (C) May-Grünwald-Giemsa staining cytospin image of bulk GTLM-transduced HFFs on day 12. Scale bar, 10 μm. (D) Relative mRNA expression of relevant human erythroid and fibroblast-specific genes in bulk GTLM-transduced HFFs (red bars) versus untransduced HFFs (gray bars) on day 12, determined by qPCR. Data are presented as mean ± SD (n = 2–3 for hiEPs, n = 1 for untransduced HFFs) for two different fibroblasts lines (F1 and F2). See also Figure S5.

To determine whether GTLM factors could induce the erythroid cell fate in the human setting, we transduced primary cultures of human foreskin fibroblasts (HFFs) from two independent sources with the murine versions of the four factors (Experimental Procedures), and evaluated erythroid reprogramming 12 days thereafter. GTLM-transduced HFFs gave rise to clusters of round cells that co-expressed CD71 and Glycophorin A (GPA), indicating the presence of early erythroid precursor-like cells (Figure 5A; Figure S5). 7.7% ± 5.9% of CD71^+^ GPA^+^ cells were also positive for Band 3, a membrane glycoprotein expressed in late erythroid precursors and mature RBCs (Chen et al., 2009; Figures 5A and 5B; Figure S5). Consistent with the results in the murine system, reprogramming was completely abrogated when one of the GTLM factors was removed, demonstrating that all four are necessary to also induce the erythroid fate in human fibroblasts (Figures 5A and 5B). Bulk human iEPs displayed a round morphology, deep blue cytoplasm, and coarse chromatin, features of early erythroid precursors (Figure 5C). Last, gene expression analysis by qPCR revealed that GTLM factors robustly induced the co-ordinated expression of several erythroid cell-specific genes, including embryonic (*HBE1*), fetal (*HBG1/2*), and adult hemoglobins (*HBB* and *HBA1/2*) (Figure 5D). As observed in the murine setting, human iEPs expressed 7.5 times more fetal *HBG1/2* and 20.7 times more *HBE1* than adult *HBB*, supporting the previous finding that GTLM induces an embryonic erythroid program. Fibroblast-specific genes were less effectively downregulated upon reprogramming than in mouse TTFs, which is likely reflective of the cellular heterogeneity of the bulk populations analyzed.

Altogether, we provide proof-of-principle evidence that iEPs can be generated from human fibroblasts by overexpression of GTLM, reproducing the findings in the murine setting and underscoring a conserved transcriptional program instructing the erythroid cell fate in mammalian cells.

## Discussion

The identification of the minimal set of factors required to instruct erythroid lineage fate could provide a strategy to study and recapitulate erythropoiesis in vitro for medical purposes. We show that murine and human fibroblasts can be rapidly and directly converted into erythroid progenitor/precursor cells by forced expression of *Gata1*, *Tal1*, *Lmo2*, and *c-Myc*. iEPs exhibit properties of bona fide erythroid cells, such as morphology, gene expression, and colony-forming capacity, suggesting that GTLM constitute the core network of the erythroid program capable of orchestrating the battery of factors necessary for normal RBC development.

In erythroid cells, GATA1 and TAL1 are known to assemble within multimeric protein complexes, also including the adaptor molecules LMO2 and LDB1 (Osada et al., 1997) and reviewed by Love et al. (2014). These complexes show widespread binding at erythroid genes and erythroid enhancer elements, functioning as primary mediators of global erythroid gene activation (Li et al., 2013). In addition, the ability of LDB1 protein complexes to oligomerize facilitates long-range associations between promoters and enhancers, which is essential for *β-globin*, *Myb*, and *Epb4.2* gene expression (Song et al., 2007). *Ldb1* is already expressed in fibroblasts (Figure S3D), which likely explains why it is not additionally necessary for iEP generation. Thus, our results suggest that the key event leading to induction of the erythroid fate is the assembly of the GATA1/TAL1/LMO2/LDB1 complex.

The role of *c-Myc* in iEP generation is more ambiguous because its protein is not known to interact with GATA1, TAL1, or LMO2. *c-Myc* is one of the of the four transcription factors used to originally generate induced pluripotent stem cells (iPSCs) from fibroblasts (Takahashi and Yamanaka, 2006). In that context, *c-Myc* has been shown to enhance the early steps of reprograming by repressing fibroblast-specific genes and upregulating the metabolic program of the embryonic state (Sridharan et al., 2009). For iPSC generation, *c-Myc* can be omitted if p53-null fibroblasts are used (Hong et al., 2009). In our system, reprogramming of p53-null fibroblasts greatly enhanced efficiency but did not allow reprogramming without *c-Myc* (data not shown), which suggests that *c-Myc* has a role beyond that shown in iPSC generation. Another indication of *c-Myc*’s erythroid-specific requirement is highlighted by the fact that epiblast-restricted *c-Myc*-null mouse embryos die at E12 from severe anemia (Dubois et al., 2008). Thus, *c-Myc* is likely to have more than just a cell proliferation function during erythroid reprogramming, and future investigation will be needed to determine its precise role in iEP generation. In addition, the observation that iEP reprogramming is enhanced by increasing the amount of *Gata1* while increasing *c-Myc*, instead, blocks development of hemoglobinized cells (Figure 2H) demonstrates that GTLM factor stoichiometry is critical and can be further optimized.

Since the advent of iPSC technology in 2006, many laboratories have been screening for factor combinations that can instruct cell fate changes with the main purpose of generating relevant cell types for regenerative medicine. Although non-hematopoietic cells have never been directly reprogrammed to erythroid cells before, different combinations of transcription factors have been shown to reprogram somatic cells to hematopoietic cells, including hematopoietic stem cells and downstream precursor cells (Doulatov et al., 2013, Kulessa et al., 1995, Pereira et al., 2013, Pulecio et al., 2014, Riddell et al., 2014). Recently, Batta et al. (2014) described the generation of hematopoietic progenitors with subsequent erythroid potential by ectopic expression of *Erg*, *Gata2*, *Lmo2*, *Runx1c*, and *Scl* in murine fibroblasts. However, for the factor combinations to be relevant for determining the core transcriptional machinery directing RBC development, it is necessary to reprogram directly to erythroid-restricted progenitor cells. In this regard, the most informative study is a report demonstrating that differentiated murine B cells can be reprogrammed to erythroid-like cells by forced expression of *Gata1*, *Scl*, and *C/EBPα* (Sadahira et al., 2012). *Gata1* and *Scl* were sufficient for reprogramming, but *C/EBPα* enhanced the process by inactivating *Pax6*, a critical transcription factor for B cell differentiation. A second study, focusing on molecular mechanisms controlling the progression from hematopoiesis to erythropoiesis during embryogenesis, reported that a combination of five transcription factors, *Scl*, *Lmo2*, *Gata2*, *Ldb1*, and *E2A*, together with the inhibition of the fibroblast growth factor (FGF) pathway, directly induced erythroid differentiation in the pluripotent chicken epiblast (Weng and Sheng, 2014). Only *Scl* + *Lmo2* and FGF inhibition were sufficient to convert nascent mesoderm cells in later stages of the chicken embryo, suggesting distinct factor requirements depending on the plasticity of the starting population. *Gata1* and *Klf1* could replace *Gata2* in the transcription factor cocktail, but the efficiency was reduced. This study, however, investigated direct erythroid induction from pluripotent cell populations and not terminally differentiated cells, thus illustrating a case of lineage specification rather than transdifferentiation. Taken together, despite the diverse nature of starting cell sources, most of these studies employ the Gata factor family, *Scl*/*Tal1*, and/or *Lmo2* as conversion factors, validating the essential role of this complex in establishing erythroid lineage identity.

Another common feature of the aforementioned reports and our findings is the detection of embryonic and fetal globins. The inability to express adult globin has also been observed in erythrocytes generated in vitro from pluripotent stem cells or cord blood progenitors, which poses a major barrier to their clinical use (reviewed by Anstee et al. (2012). Together, these observations support the hypothesis that transcription factor-driven reprogramming to blood follows early developmental steps and suggests that the addition of factors mediating the switch to adult globin may be necessary for maturation. Consistent with this, we found that overexpression of *Klf1* or *Myb* changed the globin expression pattern in single iEPs from predominantly embryonic to mainly adult. Although both *Klf1* and *Myb* were found to be dispensable during the initial screening for erythroid fate conversion factors, they were later found to induce adult globin expression. *Myb* is uniquely expressed during definitive erythropoiesis, and its dysregulation is associated with the persistence of embryonic and fetal hemoglobins in Trisomy 13 (Sankaran et al., 2011, Tober et al., 2008). *Klf1*, in contrast, is required for both primitive and definitive erythropoiesis and plays a crucial role in regulating the expression of adult and embryonic globins (Hodge et al., 2006).

Here we show direct and robust red cell fate conversion from a mammalian, non-hematopoietic, differentiated somatic cell. We demonstrate that transcription factor-mediated direct conversion can be employed as an unambiguous method to define the core transcriptional program of a cell type, allowing the distinction of “fate conversion factors” versus “maturation factors.” This method can be applied to define the factors required for human adult erythropoiesis and model disease. Furthermore, the GTLM factors could potentially be used to enhance methods for in vitro production of erythrocytes for personalized transfusion medicine.

## Experimental Procedures

### Mice

*Epor*-Cre *R26-*eYFP mice were on a C57Bl/6 background and have been described previously (Heinrich et al., 2004, Singbrant et al., 2011). WT C57BL/6 mice were purchased from Taconic. All animal experiments were carried out in accordance with Lund University’s ethical regulations (Ethical Permit M253-12).

### Establishment of Fibroblast Cultures from Mouse Tail Tips

The procedure utilized was a modified version of the protocol by Takahashi and Yamanaka (2006). Tail tips were taken from 6- to 8-week-old mice. The fur was dissected out, and the remaining tissue was manually minced with a sterile scalpel into 1-cm-long pieces and further dissected in the presence of 0.4% trypsin (Thermo Scientific). Tissue pieces were plated on cell culture dishes pre-coated with 0.1% gelatin and incubated in fibroblast medium for 5 days at 37°C, 5% CO_2_, 4% O_2_. Confluent cultures were passaged twice before hematopoietic lineage depletion and then used for iEP generation. TTF cultures were derived in DMEM supplemented with 15% fetal calf serum (FCS) (Thermo Scientific), 2 mM L-glutamine (Gibco), 1% nonessential amino acids (Gibco), and 1% antibiotics (fibroblast expansion medium [FEX]).

### Molecular Cloning and Production of Retrovirus

For the initial screening, coding regions for candidate genes (Table S1) were amplified and cloned into the pMXs retroviral vector backbone using BamHI and NotI restriction sites. The details of the primer sequences are provided in Table S4. For hemoglobin switching experiments, coding regions of *Klf1* and *Myb* were digested out of the pMXs vector using BamHI and SalI and cloned into the pWZL-blast retroviral vector backbone using the same restriction sites. For retrovirus production, 2 × 10^6^ Phoenix GP cells were seeded per 100-mm dish without antibodies. The next day, the medium was changed to pure DMEM, and pMXs plasmids and the Ecopac vector were transfected using FuGENE 6 transfection reagent (Promega). 27 μl of FuGENE 6 was added to 400 μl of DMEM and incubated for 5 min at room temperature. 6 μg of pMXs vector and 3 μg of Ecopac vector were diluted in 25 μl of DMEM and added to the previous mixture, which was incubated for 20 min at room temperature. The DNA/FuGENE 6 mixture was added drop by drop onto the Phoenix GP cells, which were incubated overnight at 37°C, 5% CO_2_. The next day, the medium was changed to DMEM + 20% FCS. Viral supernatants were harvested 48 hr after transfection, filtered through a 0.45-μm filter, and frozen for later use.

### iEP Generation

#### Mouse iEP Generation

TTFs were seeded at 1 × 10^4^ cells/cm^2^ on 0.1% gelatin pre-coated dishes and infected 24 hr thereafter with non-concentrated, virus-containing supernatants supplemented with 4 μg/ml of Polybrene (Merck Millipore). Virus supernatant and FEX were combined at a ratio of 0.6:1 during transduction. After 4 hr, the medium was changed to serum-free expansion medium (SFEM) (STEMCELL Technologies) supplemented with 1% antibiotics, 100 ng/ml mSCF, 10 ng/ml mIL-3, 2 U/ml recombinant human Erythropoietin (rhEPO), and 100 nM dexamethasone and cultured at 37°C, 5% CO_2_ and hypoxia (4% O_2_). Emerging murine iEP colonies were scored after 5–8 days after transduction.

#### Human iEP Generation

For human iEPs, 1 × 10^5^ HFFs were infected three times with several combinations of murine retroviruses at 1,800 rpm for 45 min at 32°C in the presence of 4 μg/ml Polybrene and cultured at 32°C for 12 hr between infections. The culture medium containing the viruses was changed the day thereafter for reprogramming medium (Iscove’s modified Delbecco’s medium [IMDM], 0.4% albumin, 1% human serum supplemented with 1% antibiotics, insulin-transferring-selenium [ITS] (Gibco), 100 ng/ml hSCF, 10 ng/ml hIL-3, 2 U/ml rhEPO, and 100 nM dexamethasone) and cultured at 37°C, 5%CO_2_, and 4% O_2_.

### Establishment of Mouse Fibroblast Cultures with Constitutive Expression of *Klf1* or *Myb*

TTFs were seeded and transduced with pWZL-blast-*Klf1* or pWZL-blast-*Myb* as described under iEP Generation. 48 hr after transduction, cells were passaged and cultured in FEX supplemented with 4 μg/ml blasticidin antibiotic (InvivoGen). The whole medium was replaced with fresh antibiotic-containing medium every 3 days. When a population of resistant TTFs was obtained, cells were seeded accordingly and transduced with pMX vectors to generate iEPs. Blasticidin was added to the medium during reprogramming.

### Flow Cytometry

Clusters of round cells were collected through gentle pipetting, and untransduced fibroblasts were trypsinized. Cells were resuspended in 100 μl of PBS + 2% FCS and stained for 20 min at 4°C in the dark. The list of antibodies and dilutions can be found in the Supplemental Experimental Procedures. After staining, cells were washed and resuspended in 300 μl of PBS with 2% FCS. To assess cell viability, cells were also stained with 1:100 DAPI. Compensation controls were set with murine bone marrow cells. The analysis of mouse cells was performed using FACSCanto II (Becton Dickinson), and data were analyzed using FlowJo v10 software. The analysis of human cells was performed using a Gallios flow cytometer (Becton Dickinson).

### RNA Isolation and qPCR

Total RNA was isolated with the RNAeasy kit (QIAGEN) according to the manufacturer’s guidelines. RNA was subjected to cDNA synthesis using Superscript III (Invitrogen). Quantitative PCR analysis was performed in triplicates for each sample and Taqman gene expression assays (Applied Biosystems) or PrimeTime qPCR assays (Integrated DNA Technologies). For each reaction, 10 μl of Taqman master mix, 4 μl of sterile water, and 1 μl of the primer assay were mixed. Then, 5 μl of cDNA (<40 ng/μl) was added to each well. The signal was detected with a 7900HT RT-PCR instrument (Applied Biosystems). The primers used are listed in Tables S2 and S3.

### Colony Assay

For colony-forming assays, MethoCult M3236 (STEMCELL Technologies) was used, which was supplemented with 5 U/ml rhEPO, 50 ng/ml mSCF, 100 nM of dexamethasone, and 1% antibiotics. Cells were resuspended in FCS and added to the MethoCult mixture. All experiments were performed in triplicate. Colonies were scored on day 8.

### Global Gene Expression Analysis

Total RNA was isolated from red iEP, non-red iEP, FL colony, BM colony, and fibroblasts and hybridized to a Mouse Gene 2.1 ST array (Affymetrix) according to the manufacturer’s protocol. The arrays were robust multi-array average (RMA)-normalized, and differentially expressed genes were identified using LIMMA (25605792). The microarray data have been deposited in NCBI’s GEO (Edgar et al., 2002) and are accessible through GEO: GSE73344.

### Single-Cell qRT-qPCR and Data Analysis

Single YFP^+^ Ter119^+^ cells were sorted into 96-well PCR plates (Sarstedt) containing 4 μl of lysis buffer. Next, target-specific pre-amplification was performed using the CellsDirect one-step qRT-PCR kit (Life Technologies). Pre-amplified samples were diluted 1:5 and run on Fluidigm 96.96 arrays on a Biomark device (Fluidigm) together with the Taqman assays listed in Table S2 and reagents according to the manufacturer’s instructions. The data were analyzed using the Single Cell Expression Visualizer web tool (http://stemsysbio.bmc.lu.se/SCexV/) (Lang et al., 2015). See the Supplemental Experimental Procedures for details.

### Statistical Evaluation

Statistical analyses of all endpoints were performed using unpaired Student’s t test (Figure 2F) and two-way ANOVA test (Dunnett correction for multiple comparisons, 95% confidence intervals) (Figures 1C, 2J, 4A and 4B, and 5B) using GraphPad Prism 6.

## Author Contributions

Conceptualization, S.C.G., K.D., K.S., S. Singbrant, V.G.S., and J.F.; Methodology, S.C.G., K.D., K.S., V.G.S., and J.F.; Software and Formal Analysis, S. Soneji; Investigation – Murine Data, S.C.G., K.D., and K.S.; Investigation – Human Data, J.P. and S.C.G.; Library Generation, V.R.E. and J.F.; Single-Cell qRT-PCR Conceptualization and Analysis, M.S., S.C.G., and G.K., Writing – Original Draft, S.C.G. and J.F.; Writing – Review & Editing, S.C.G., V.G.S., S. Singbrant., K.S., S. Soneji, V.R.E., J.P., A.R., C.W., and J.F.; Funding Acquisition, A.R. and J.F.; Resources, C.W., A.R., and J.F.; Supervision, J.F.

## Accession Numbers

The microarray data have been deposited in NCBI’s Gene Expression Omnibus (Edgar et al., 2002) and are accessible through GEO: GSE73344.

## Supplemental Information

Document S1. Supplemental Experimental Procedures, Figures S1–S5, and Tables S1–S4

Document S2. Article plus Supplemental Information
