# Supplementary material for: Defining the Minimal Factors Required for Erythropoiesis through Direct Lineage Conversion
Source: Cell Rep. 2016 Jun 2;15(11):2550–62. doi: 10.1016/j.celrep.2016.05.027 (PMC4914771; doi:10.1016/j.celrep.2016.05.027)
Supplement: Document S1. Supplemental Experimental Procedures, Figures S1–S5, and Tables S1–S4 [file mmc1.pdf]

Cell Reports, Volume *15*

Supplemental Information

Defining the Minimal Factors Required for Erythropoiesis through Direct Lineage Conversion

Sandra Capellera-Garcia, Julian Pulecio, Kishori Dhulipala, Kavitha Siva, Violeta Rayon-Estrada, Sofie Singbrant, Mikael N.E. Sommarin, Carl R. Walkley, Shamit Soneji, Göran Karlsson, Ángel Raya, Vijay G. Sankaran, and Johan Flygare

Figure S1

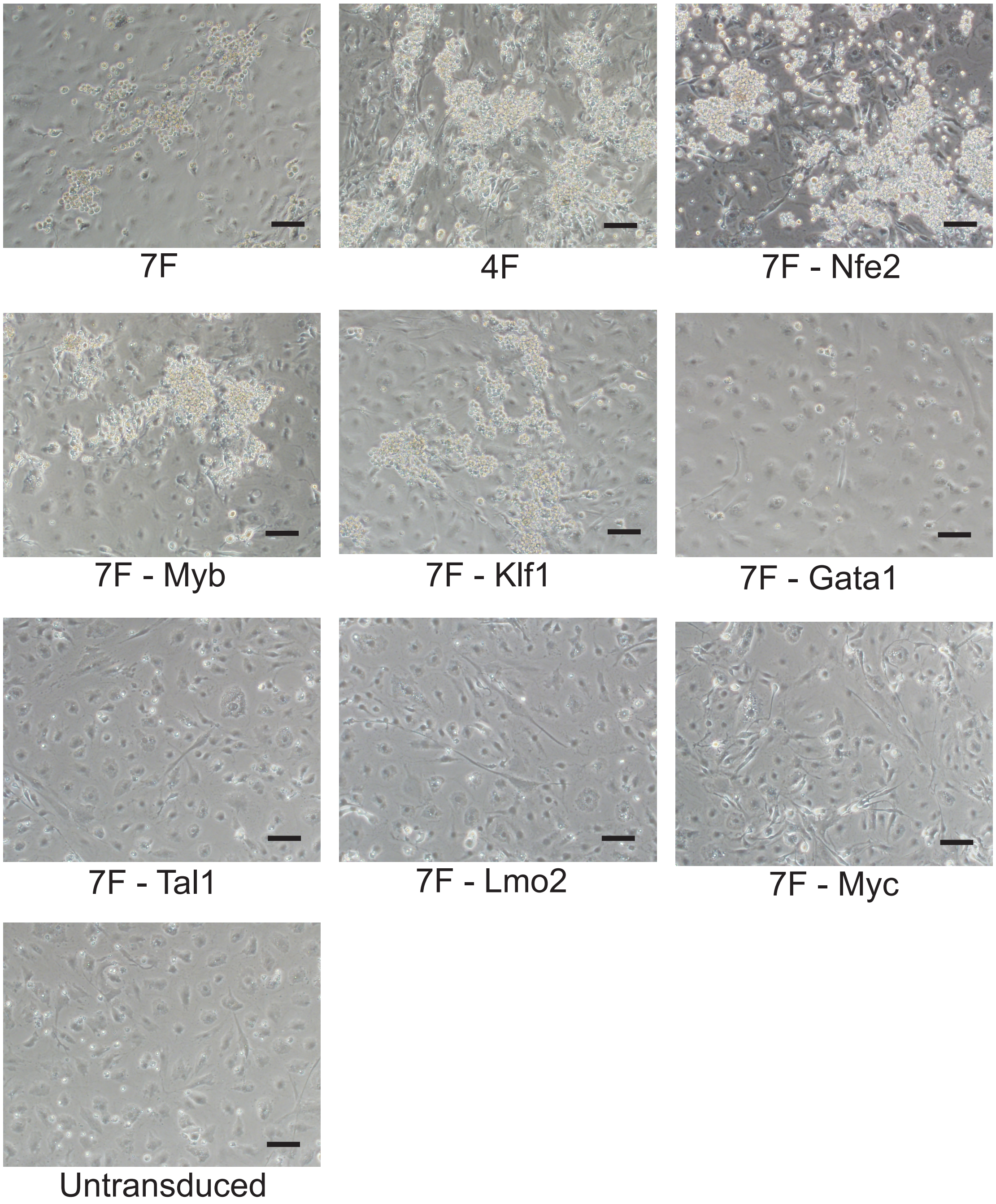
7F
4F
7F - Nfe2
7F - Myb
7F - Klf1
7F - Gata1
7F - Tal1
7F - Lmo2
7F - Myc
Untransduced

Figure S2

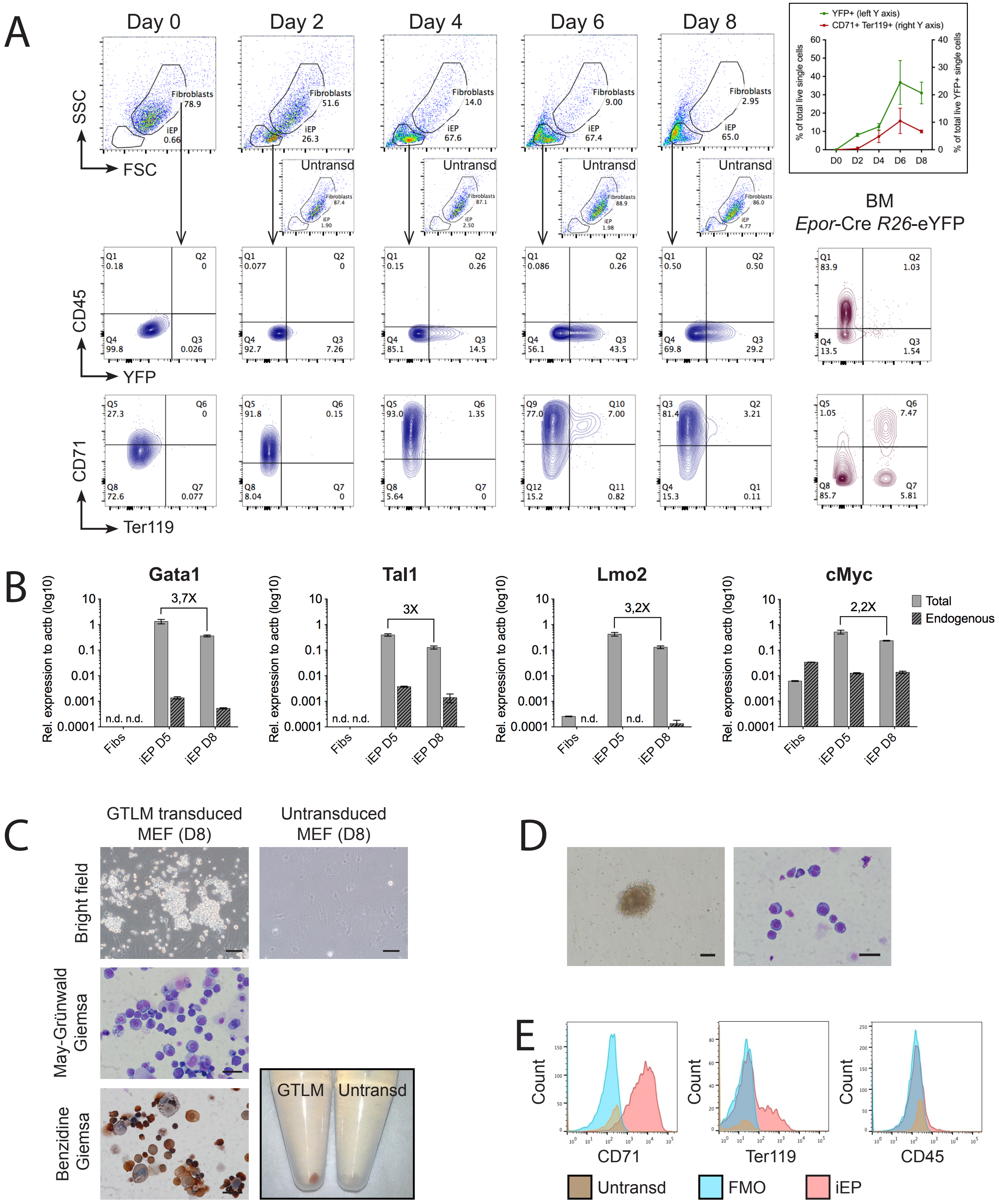
A
Day 0
Day 2
Day 4
Day 6
Day 8
SSC
FSC
Fibroblasts 78.9
iEP 0.66
Fibroblasts 51.6
iEP 26.3
Fibroblasts 14.0
iEP 67.6
Fibroblasts 9.00
iEP 67.4
Fibroblasts 2.95
iEP 65.0
Untransd
YFP+ (left Y axis)
CD71+ Ter119+ (right Y axis)
% of total live single cells
% of total live YFP+ single cells
D0 D2 D4 D6 D8
BM
Epor-Cre R26-eYFP
CD45
YFP
CD71
Ter119
B
Gata1
Tal1
Lmo2
cMyc
3,7X
3X
3,2X
2,2X
Rel. expression to actb (log10)
n.d.
Fibs
iEP D5
iEP D8
Total
Endogenous
C
GTLM transduced MEF (D8)
Untransduced MEF (D8)
Bright field
May-Grünwald Giemsa
Benzidine Giemsa
GTLM
Untransd
D
E
Count
CD71
Ter119
CD45
Untransd
FMO
iEP

Figure S3

A

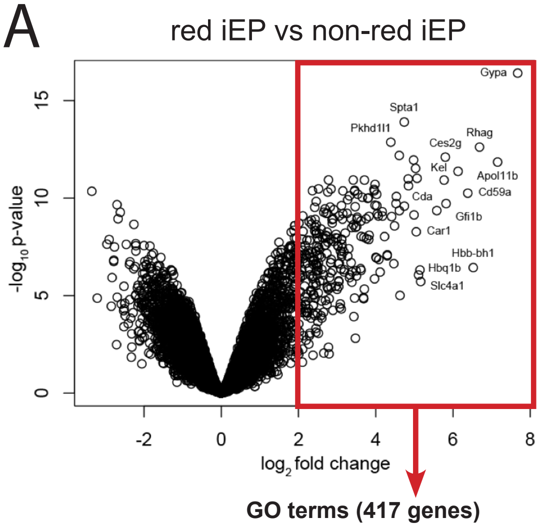

B

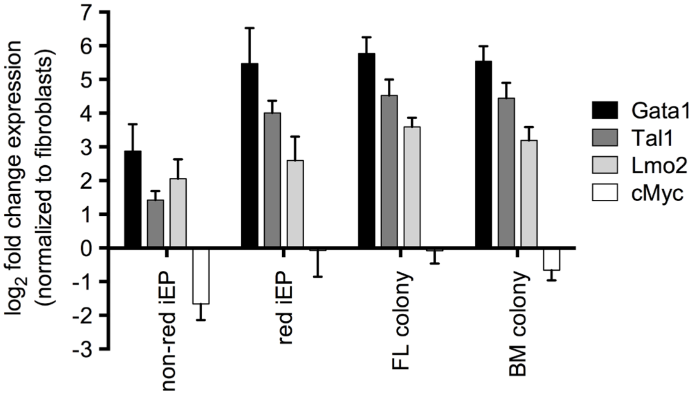

GO terms (417 genes)
Tetrapyrrole biosynthetic process
Porphyrin biosynthetic process
Erythrocyte differentiation
Porphyrin metabolic process
Tetrapyrrole metabolic process
Erythroid homeostasis

C

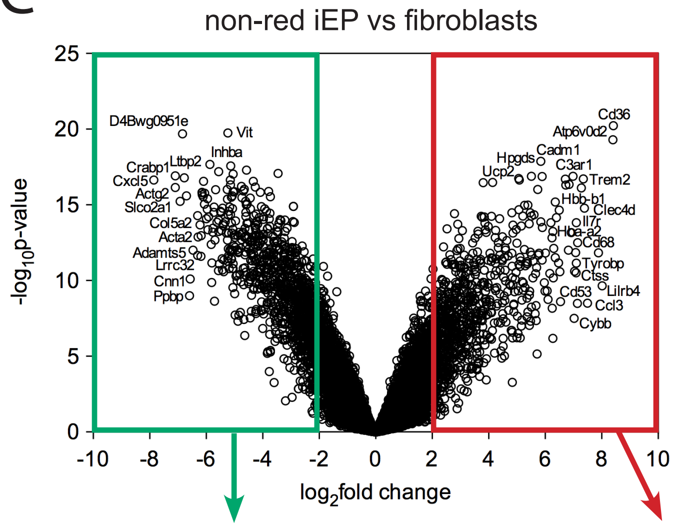

D

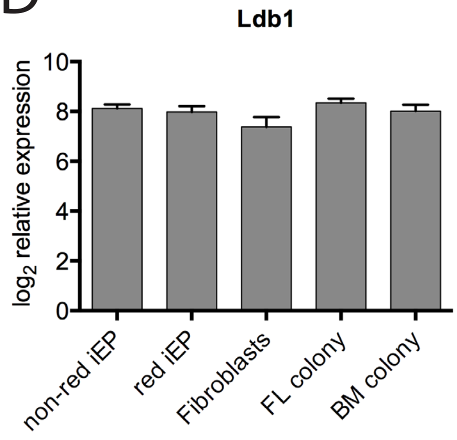

GO terms (1036 genes)
Cell adhesion
Biological adhesion
Vasculature development
Enzyme linked receptor protein signaling pathway
Skeletal system development
Blood vessel development
Cell motion
Transmembrane receptor protein tyrosine kinase signaling pathway
Extracellular structure organization
Chordate embryonic development
Embryonic development ending in birth or egg hatching
Skeletal system morphogenesis
Blood vessel morphogenesis
Cell migration
Tissue morphogenesis
Extracellular matrix organization
Embryonic morphogenesis
Cell morphogenesis involved in differentiation
Regulation of cell proliferation

GO terms (815 genes)
Immune response
Cell activation
Leukocyte activation
Positive regulation of immune system process
Response to wounding
Lymphocyte activation
Positive regulation of response to stimulus
Positive regulation of immune response
Inflammatory response
Defense response

Figure S4

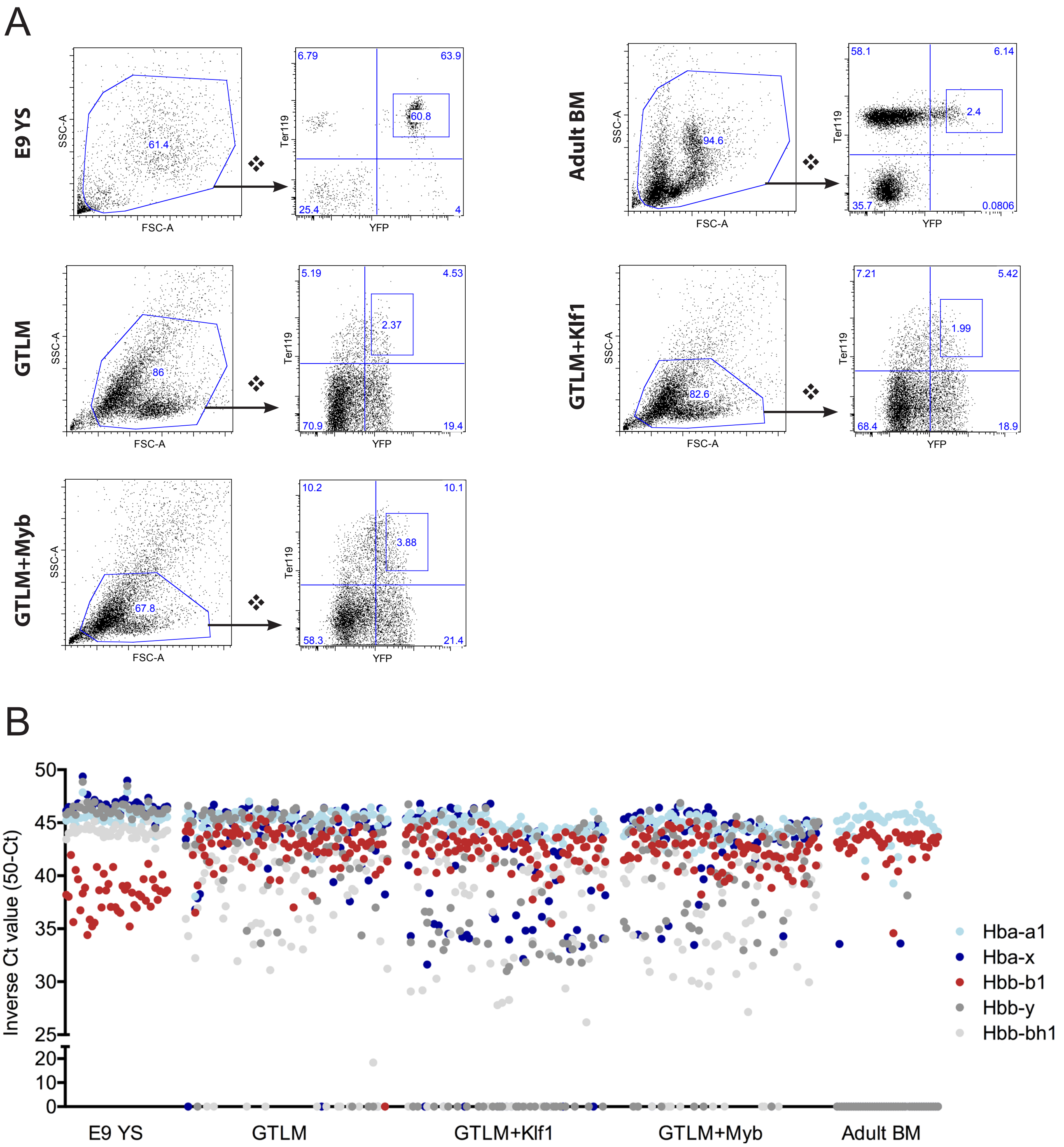

A
E9 YS
SSC-A
FSC-A
61.4
Ter119
YFP
6.79
63.9
60.8
25.4
4
Adult BM
94.6
58.1
6.14
2.4
35.7
0.0806
GTLM
86
5.19
4.53
2.37
70.9
19.4
GTLM+Klf1
82.6
7.21
5.42
1.99
68.4
18.9
GTLM+Myb
67.8
10.2
10.1
3.88
58.3
21.4
B
Inverse Ct value (50-Ct)
50
45
40
35
30
25
20
10
0
E9 YS
GTLM
GTLM+Klf1
GTLM+Myb
Adult BM
Hba-a1
Hba-x
Hbb-b1
Hbb-y
Hbb-bh1

Figure S5

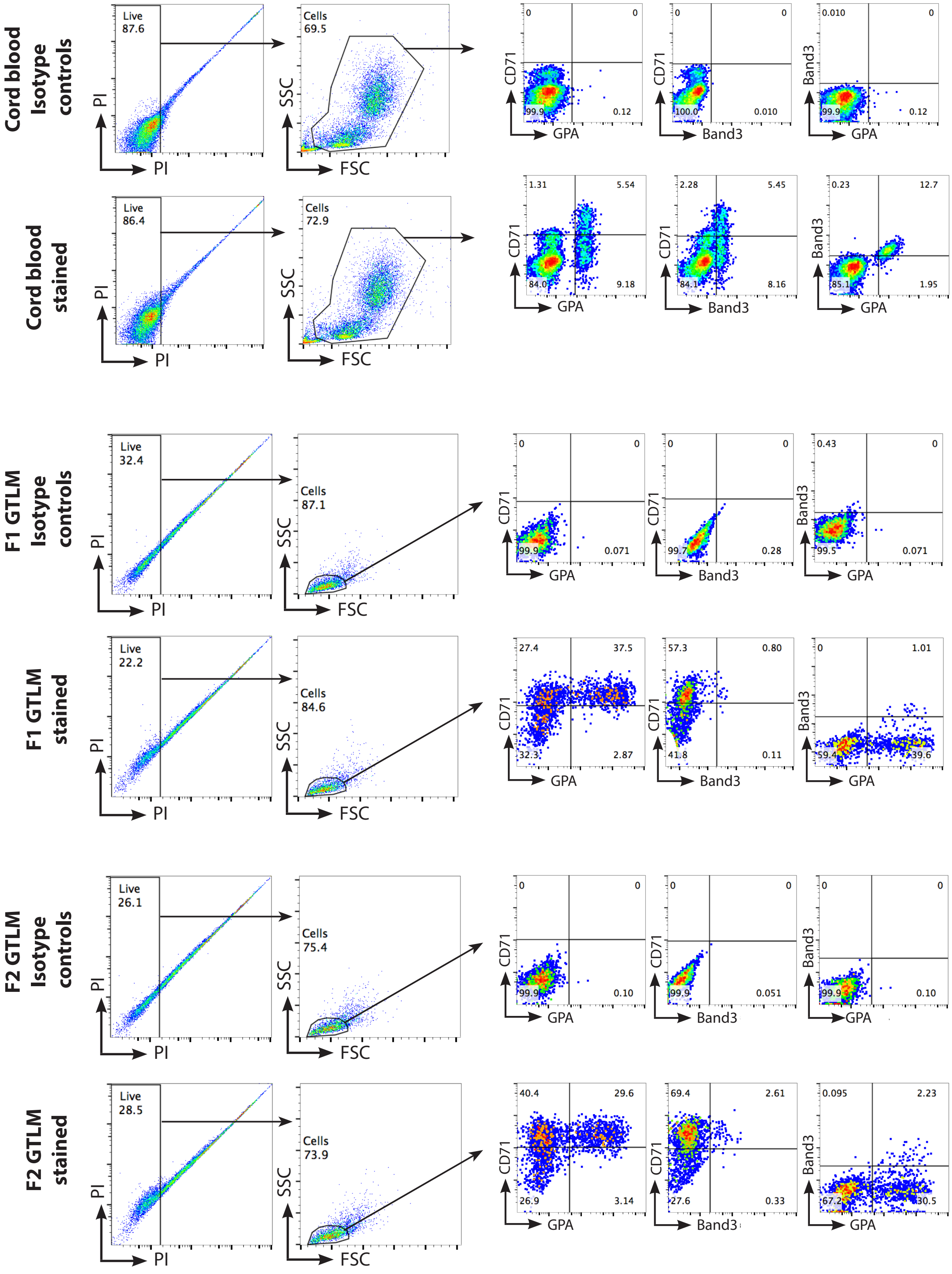

Cord blood Isotype controls
Live 87.6
Cells 69.5
PI
SSC
FSC
CD71
GPA
Band3
0
0
99.9
0.12
100.0
0.010
0.010
Cord blood stained
Live 86.4
Cells 72.9
1.31
5.54
84.0
9.18
2.28
5.45
84.1
8.16
0.23
12.7
85.1
1.95
F1 GTLM Isotype controls
Live 32.4
Cells 87.1
99.9
0.071
99.7
0.28
0.43
99.5
F1 GTLM stained
Live 22.2
Cells 84.6
27.4
37.5
32.3
2.87
57.3
0.80
41.8
0.11
1.01
59.4
39.6
F2 GTLM Isotype controls
Live 26.1
Cells 75.4
99.9
0.10
0.051
F2 GTLM stained
Live 28.5
Cells 73.9
40.4
29.6
26.9
3.14
69.4
2.61
27.6
0.33
0.095
2.23
67.2
30.5

Figure S1, related to Figure 1. Forced Expression of *Gata1*, *Tal1*, *Lmo2* and *c-Myc* Reprograms Murine Adult Fibroblasts into Erythroid Progenitors

Representative live-cell bright field images of single wells after removal of individual TFs from the 7TF pool at day 8. Untransduced fibroblasts as negative control. Scale bar, 50 μm.

Figure S2, related to Figure 2. Induced Erythroid Progenitors Exhibit Properties of *Bona Fide* Erythroid Cells

(A) Time-course flow cytometry analysis of untransduced TTF (day 0) and bulk GTLM-transduced TTF harvested at day 2, 4, 6 and 8 showing YFP, CD45, CD71 and Ter119 expression, along with a graph displaying summary of data (n=3). Bone marrow from an *Epor*-Cre *R26*-eYFP adult mouse is shown as a staining control.

(B) Relative mRNA expression of *Gata1*, *Tal1*, *Lmo2* and *c-Myc* in untransduced TTF and bulk GTLM-transduced TTF harvested at day 5 and day 8, determined by qPCR. Primers were designed so that endogenous expression could be distinguished from total expression. Data are presented as mean ± SD (n=4-6 for iEPs, n=2 for untransduced TTF).

(C) Representative live-cell bright field, May-Grünwald Giemsa staining and Benzidin/Giemsa staining images of GTLM-iEPs generated from MEFs at day 8. Untransduced MEFs as negative control. Scale bar live-cell bright field, 50 μm. Scale bar cytospin, 20 μm. Bottom right, macroscopic inspection of cell pellets of GTLM-iEPs generated from MEFs and untransduced MEFs at day 8.

(D) Bright field and May-Grünwald Giemsa cytospin images of a representative red colony. D5-iEPs generated from MEFs were plated in semisolid media and

scored 8 days after. Scale bar live-cell bright field, 50 μm. Scale bar cytospin, 20 μm.

(E) Flow cytometry analysis of GTLM-iEPs derived from MEFs at day 8, gated on total live cells, showing CD71, Ter119 and CD45 expression.

Figure S3, related to Figure 3. iEP-Derived Red Colonies Retain a Primitive Erythroblast Expression Signature

(A) Volcano plot presenting differentially expressed genes between red iEP and non-red iEP. Genes with at least 4-fold higher expression in red iEP are highlighted with their most representative GO terms (pvalue$\leq 10^{-8}$) according to DAVID. Log_2fold difference is plotted on the *x* axis, and p-value-adjusted significance is plotted on the *y* axis ($-log_{10}$ scale).

(B) Log_2 fold change in expression of GTLM factors in non-red iEP, red iEP, FL colony and BM colony, relative to fibroblasts. Data are represented as mean ± SD (n=3).

(C) Volcano plot presenting differentially expressed genes between non-red iEP and fibroblasts. Genes with at least 4-fold higher expression in non-red iEP and genes with at least 4-fold more expression in fibroblasts are highlighted with their most representative GO terms (pvalue$\leq 10^{-8}$) according to DAVID. Log_2fold difference is plotted on the *x* axis, and p-value-adjusted significance is plotted on the *y* axis ($-log_{10}$ scale).

(D) Log_2 relative expression of Ldb1 determined by microarray. Data are represented as mean ± SD (n=3).

Figure S4, related to Figure 4. *Klf1* and *Myb* Enhance Adult Hemoglobin Expression in single iEPs

(A) Representative flow cytometry plots showing gating strategy used for index sorting of single cells for qRT-PCR. E9 yolk sac (E9 YS) was obtained by crossing an *Epor*-Cre *R26*-eYFP (*Epor* reporter) male with a WT female. Adult bone marrow was obtained from an *Epor* reporter mouse. GTLM-iEPs were obtained by reprogramming *Epor* reporter TTF, whereas GTLM+Klf1-iEPs and GTLM+Myb-iEPs were obtained by reprogramming *Epor* reporter TTF with constitutive expression of *Klf1* and *Myb*, respectively. ❖, doublets and dead-cell exclusion.

(B) Inverse Ct values of globin genes in single cells used to calculate the ranking in Figure 4B.

Figure S5, related to Figure 5. Induction of Erythroid Progenitors from Human Foreskin Fibroblasts by *Gata1*, *Tal1*, *Lmo2* and *c-Myc*

Representative flow cytometry plots showing gating strategy for cord blood mononuclear cells and transduced/untransduced human fibroblast samples.

Table S1, related to Figure 1 and Experimental Procedures “Molecular Cloning and production of retrovirus”. List of Genes Included in the Present Study and Corresponding Accession Numbers

Gene symbol	Gene ID	Species	Gene name
Bcl11a	NM_016707	Mus musculus	B-cell CLL/lymphoma 11A (zinc finger protein)
Bmi1	NM_007552	Mus musculus	Bmi1 polycomb ring finger oncogene
Cbfa2t3	NM_009824	Mus musculus	core-binding factor, runt domain, alpha subunit 2, translocated to, 3 (human)
Cbfb	NM_022309	Mus musculus	core binding factor beta
Cdx4	NM_007674	Mus musculus	caudal type homeo box 4
Cited2	NM_010828	Mus musculus	Cbp/p300-interacting transactivator, with Glu/Asp-rich carboxy-terminal domain, 2
E2F1	NM_007891	Mus musculus	E2F transcription factor 1
E2F2	NM_177733	Mus musculus	E2F transcription factor 2
E2F4	NM_148952	Mus musculus	E2F transcription factor 4
E2F8	NM_001013368	Mus musculus	E2F transcription factor 8
Egr1	NM_007913	Mus musculus	early growth response 1
Erg	NM_133659	Mus musculus	avian erythroblastosis virus E-26 (v-ets) oncogene related
Ets1	NM_011808	Mus musculus	E26 avian leukemia oncogene 1, 5' domain
Etv6	NM_007961	Mus musculus	ets variant gene 6 (TEL oncogene)
Fli1	NM_008026	Mus musculus	Friend leukemia integration 1
Zfpm1 (Fog1)	NM_009569	Mus musculus	zinc finger protein, multitype 1
Fos	NM_010234	Mus musculus	FBJ osteosarcoma oncogene
Foxo3a	NM_019740	Mus musculus	forkhead box O3
Gata1	NM_008089	Mus musculus	GATA binding protein 1
Gata2	NM_008090	Mus musculus	GATA binding protein 2
Gfi1b	NM_008114	Mus musculus	growth factor independent 1B
HoxA9	NM_010456	Mus musculus	homeo box A9
HoxB4	NM_010459	Mus musculus	homeo box B4
Ikzf1	NM_009578	Mus musculus	IKAROS family zinc finger 1
Irf2	NM_008391	Mus musculus	interferon regulatory factor 2
Klf1	NM_010635	Mus musculus	Kruppel-like factor 1 (erythroid)
Klf13	NM_021366	Mus musculus	Kruppel-like factor 13
Klf3	NM_008453	Mus musculus	Kruppel-like factor 3 (basic); similar to BKLF
Ldb1	NM_010697	Mus musculus	LIM domain binding 1
Lmo2	NM_008505	Mus musculus	LIM domain only 2
Lyl1	NM_008535	Mus musculus	lymphoblastomic leukemia 1
Mafk	NM_010757	Mus musculus	v-maf musculoaponeurotic fibrosarcoma oncogene family, protein K (avian)
Mef2c	NM_025282	Mus musculus	myocyte enhancer factor 2C
Meis1	NM_010789	Mus musculus	Meis homeobox 1

Mllt11	NM_019914	Mus musculus	myeloid/lymphoid or mixed-lineage leukemia (trithorax homolog, Drosophila); translocated to, 11
Myb	NM_010848	Mus musculus	myeloblastosis oncogene
Myc	NM_010849	Mus musculus	myelocytomatosis oncogene
Nfe2	NM_008685	Mus musculus	nuclear factor, erythroid derived 2
Nfkb1	NM_008689	Mus musculus	nuclear factor of kappa light polypeptide gene enhancer in B-cells 1, p105
Pou5f1 (Oct4)	NM_013633	Mus musculus	POU domain, class 5, transcription factor 1
Pbx1	NM_183355	Mus musculus	pre B-cell leukemia transcription factor 1; region containing RIKEN cDNA 2310056B04 gene; pre B-cell leukemia transcription factor 1
Runx1	NM_009821	Mus musculus	runt related transcription factor 1
Runx3	NM_019732	Mus musculus	runt related transcription factor 3
Sla	NM_009192	Mus musculus	src-like adaptor
Smarca4	NM_011417	Mus musculus	SWI/SNF related, matrix associated, actin dependent regulator of chromatin, subfamily a, member 4
Smarca5	NM_053124	Mus musculus	predicted gene 13034; SWI/SNF related, matrix associated, actin dependent regulator of chromatin, subfamily a, member 5
Sox12	NM_011438	Mus musculus	SRY-box containing gene 12
Sox6	NM_011445	Mus musculus	SRY-box containing gene 6
Sp3	NM_001098425	Mus musculus	trans-acting transcription factor 3
T (Brachyury)	NM_009309	Mus musculus	brachyury (T)
Tal1	NM_011527	Mus musculus	T-cell acute lymphocytic leukemia 1
Tf3 (E47)	NM_011548	Mus musculus	transcription factor 3
Tfdp1	NM_009361	Mus musculus	predicted gene 7390; transcription factor Dp 1; similar to Transcription factor Dp-1 (E2F dimerization partner 1) (DRTF1-polypeptide 1)
Tfdp2	NM_178667	Mus musculus	transcription factor Dp 2
Trib2	NM_144551	Mus musculus	tribbles homolog 2 (Drosophila)
Trim10	NM_011280	Mus musculus	tripartite motif-containing 10
Trim28	NM_011588	Mus musculus	tripartite motif-containing 28
Trim58	NM_001039047	Mus musculus	tripartite motif-containing 58
Zfp1	NM_011742	Mus musculus	zinc finger protein 1
Zfp143	NM_009281	Mus musculus	zinc finger protein 143
Zfp207	NM_011751	Mus musculus	zinc finger protein 207
Zfp219	NM_027248	Mus musculus	zinc finger protein 219
Zfp36l2	NM_001001806	Mus musculus	zinc finger protein 36, C3H type-like 2

Table S2, related to Figure 2, 4 and 5. List of the Taqman Assays Used for qPCR and single-cell qRT-PCR

Taqman assay ID	Gene	Taqman assay ID	Gene
Mm00836035_m1	Aff1	Mm00801891_m1	Nfe2
Mm00550729_m1	Ahctf1	Mm00433832_m1	Nr3c1
Mm00802083_m1	Alas2	Mm04207617_m1	Pbx1
Mm00657317_m1	Aldh1a1	Mm00479560_m1	Pbx2
Mm00508097_m1	Apq9	Mm00551055_m1	Pcgf6
Mm00431834_m1	Aqp1	Mm00482816_m1	Pdlim7
Mm01208559_m1	Aqp3	Mm00450111_m1	Postn
Mm01278161_m1	Aqp8	Mm00435969_m1	Prox1
Mm00492248_m1	Arid3a	Mm00456910_m1	RhD
Mm00479358_m1	Bcl11a	Mm00546741_m1	Rragd
Mm00487804_m1	c-Myc	Mm01213404_m1	Runx1
Mm00514283_s1	Cebpa	Mm03023996_m1	Smad4
Mm00516121_m1	Cited2	Mm00486320_s1	Sox4
Mm00801666_g1	Col1a1	Mm00488393_m1	Sox6
Mm00516347_m1	Crem	Mm01219775_m1	Stat3
Mm00484027_m1	Ctcf	Mm00839889_m1	Stat5b
Mm00624964_m1	E2f2	Mm01187033_m1	Tal1
Mm00514160_m1	E2f4	Mm00446971_m1	TBP
Mm00468171_m1	E2f5	Mm00443210_m1	Tcf4
Mm01329769_m1	Epb4.2	Mm00501505_m1	Tcf7l2
Mm00438760_m1	Epor	Mm00618407_m1	Tfdp2
Mm01175819_m1	Ets1	Mm00579691_m1	Thra
Mm00514851_m1	Foxh1	Mm01158806_m1	Zfp191
Mm01229605_g1	Foxn2	Mm00615562_m1	Zfp367
Mm01352636_m1	Gata1	Mm00494336_m1	Zfpm1
Mm00845395_s1	Hba-a1	Mm00523205_m1	Zgpat
Mm03039879_m1	Hba-x	Mm00518819_m1	Zxdc
Mm01611268_g1	Hbb-b1	Hs00163601_m1	ALAS2
Mm00433932_g1	Hbb-bh1	Hs00996794_m1	EPB42
Mm00433936_g1	Hbb-y	Hs00959427_m1	EPOR
Mm00468869_m1	Hif1a	Hs02758991_g1	GAPDH
Mm00469375_m1	Hif3a	Hs00361191_g1	HBA2/1
Mm00446968_m1	HPRT	Hs00758889_s1	HBB
Mm00516788_m1	Irf7	Hs00362216_m1	HBE1
Mm00492679_m1	Irf9	Hs00361131_g1	HBG2/1
Mm00516096_m1	Klf1	Hs00923579_m1	HBZ
Mm01254146_mH	Klf7	Hs00235006_m1	ITGA1
Mm01190673_m1	Lin28b	Hs00559595_m1	ITGB1
Mm01281680_m1	Lmo2	Hs00610592_m1	KLF1
Mm01318991_m1	Mef2a	Hs00920556_m1	MYB
Mm00841956_mH	Mrrf	Hs00958111_m1	VIM

Mm00501741_m1	Myb	Hs00419119_m1	ZFPM1

Table S3, related to Figure S2. List of Primers and Internal Oligos Used to Check Expression of Reprogramming Genes (see Figure S2B).

Gene	Primer	Sequence
Gata1 endogenous	F	agggagactcagaggccaag
	R	GCCCCTAGACCAGGAAAATC
	Internal oligo	aagcccaggttcaaccccag
Tal1 endogenous	F	Gtcctacctcgacccctctc
	R	CGTCCTGTCCCTCTAGTTGC
	Internal oligo	tatgccccaggATGACGGAG
Lmo2 endogenous	F	GTCCGAGTCCAGGCAGCTA
	R	CTCATCCACGGGTTCCTCAG
	Internal oligo	GCCAGCCACGCGCCACAAAGGG
c-Myc endogenous	F	tgaaggctggatttcctttg
	R	TTCTCTTCCTCGTCGCAGAT
	Internal oligo	cgATGCCCCTCAACGTGAAC
Tal1 total	F	GCCTCACTAGGCAGTGGGTT
	R	CCTCTTCACCCGGTTGTTGT
	Internal oligo	TGGGGAACCGGATGCCTTCCCCA
Lmo2 total	F	GGACGGAAATTGTGCAGGAG
	R	CTTTGTCTTTCACCCGCATC
	Internal oligo	TGTGACAAGCGGATCCGTGC

Gene	Company	ID
Gata1 total	IDT	Mm.PT.56a.10444529
c-Myc total	IDT	Mm.PT.56a.28494642
Actb	IDT	Mm.PT.58.33540333

Table S4, related to Experimental Procedures “Molecular Cloning and production of retrovirus”. List of the Primers Used to Clone Constructs

cDNA (restriction) orientation	Primer sequence
Gata1 (BamHI) F	caaccGGATCCGCCACCATGGATTTTCCTGGTCTAGG
Gata1 (NotI) R	cacaagGCGGCCGCtattctgtgtacctTCAAGAAC
Klf1 (BamHI) F	TcctaGGATCCGCCACCATGAGGCAGAAGAGAGAGAGGAGGCCTG
Klf1 (NotI) R	cttgtGCGGCCGCacTCAGAGGTGACGCTTCATGTGCAGAGC
Lmo2 (BglII) F	ACGCCGAGATCTGCCACCATGTCCTCGGCCATCGAAAGG
Lmo2 (NotI) R	cctggGCGGCCGCCTAGATGATCCCATTGATCTTG
Myb (BamHI) F	gccccGGATCCGCCACCatggcccggagaccccgacacagc
Myb (NotI) R	cataatGCGGCCGCtggaaatgtctcacatgaccagagttc
c-Myc (BamHI) F	cgttGGATCCGCCACCATGCCCCTCAACGTGAACTTC
c-Myc (NotI) R	attccaGCGGCCGCgagttaggtcagtTTATGCACCAG
Nfe2 (BamHI) F	tcagctGGATCCGCCACCATGCCCCCGTGTCCTCCTCAG
Nfe2 (NotI) R	agcaccaGCGGCCGCtctctagaccagcTCAATCTGTAG
Sox6 (BglII) F	gaaggaaAGATCTgaaagagagaagaATGTCTTCCAAGC
Sox6 (NotI) R	agcaaacaGCGGCCGCTCAGTTGGCACTGACAGGCTCTG
Tal1 (BamHI) F	taaatatGGATCCGCCACCATGACGGAGAGGCCGCCGAGCGAGGC
Tal1 (NotI) R	tcagttggGCGGCCGCaaagttcaaactgtaagggaagttc

SUPPLEMENTAL EXPERIMENTAL PROCEDURES

Hematopoietic lineage depletion of mouse fibroblast cultures

Fibroblast cultures were trypsinized and re-suspended at $1x10^7$ cells/mL in PBS + 2% FCS. 10μg purified rat anti-mouse antibodies (CD45, Ter119, Gr-1, B220, CD4, CD5, CD11b and CD8a (Biolegend) and CD105 (SouthernBiotech)) were added per 10^7 cells and incubated for 10 min at 4ºC. Cells were washed and re-suspended in PBS + 2% FCS to $1x10^7$ cells/mL and mixed with 100ul Dynabeads sheep anti-rat IgG (Life Technologies). Cell suspension was incubated for 30 min at 4ºC with gentle rotation and then placed in contact with a magnet for 2 min. Supernatant containing the unbound cells was kept and cryopreserved for subsequent experiments.

Human fibroblast cultures

Two primary fibroblast lines were derived from human foreskin biopsies obtained with informed consent from patients. Fibroblasts were cultured in DMEM containing 10% FBS, 2 mM GlutaMAX (Invitrogen), 1% antibiotics (Invitrogen). All cell lines were maintained in an incubator (37°C, 5% CO_2) with media changes every second day.

Isolation of mononuclear cells from cord blood bags

Human Cord Blood bags were obtained from Banc de Sang i Teixits, Hospital Duran i Reynals, Barcelona. Mononuclear cells (MNC) were isolated from CB using Lympholyte-H (Cederlane, Ontario, CA) by density gradient centrifugation, red blood-lysis and two rounds of washing with PBS.

Cytospin and May-Grünwald Giemsa staining

Around 10,000 cells were centrifuged and resuspended in 50μL of PBS with 2% FCS. Cells were cytocentrifuged using Shandon cytospin 3 (Block Scientific, Inc., NY, USA) at 500g for 3 minutes to allow adherence to the slides. Working Giemsa solution was prepared by mixing 3mL of Giemsa stock solution (Histolabs, Gothenburg) to 60mL of distilled water. Air-dried slides were stained in May-Grünwald solution (Merck, Germany) for 5 mins, briefly rinsed with distilled water and transferred to working Giemsa solution for 10 min. The slides were finally washed in distilled water and allowed to dry before examined under the microscope.

Flow cytometry antibodies and dilutions

Anti-mouse CD71-Biotin, 1:500 (BD Biosciences), Streptavidin-Qdot605, 1:600 (Life Technologies), anti-mouse Ter119-APC, 1:400 (Biolegend), anti-mouse CD45.2-APC-Cy7, 1:200 (eBioscience), anti-human CD71-FITC, (dilution as recommended by manufacturer) (BD Biosciences), anti-human CD235a-APC, (dilution as recommended by manufacturer) (Immunostep) and anti-human Band3-PE, (dilution as recommended by manufacturer) (IBGRL Research Products).

Single cell qRT-qPCR and data analysis

Single YFP+ Ter119+ cells were sorted into 96-well PCR plates (Sarstedt) containing 4 μl of lysis buffer [0.4% of NP40 buffer (Sigma), 65μM of dNTP mix (Takara), 2.4mM DTT (Invitrogen), 0.5 U/μL RNAseOUT (Life

Technologies) in nuclease-free water] using the index-sorting function of the Diva software and according to the gating strategy shown in Figure S4A. Target-specific pre-amplification was performed using CellsDirect One-Step qRT-PCR Kit (Life Technologies). Pre-amplification master mix [6.25μL of 2X reaction buffer, 1μL of SuperScriptIII RT/Platinum Taq mix, 1.5μL of Taqman assays equal volume mix and 0.007μL of XenoRNA control (Ambion, Life Technologies)] was added to each well and reverse transcription followed by pre-amplification was performed in a Biorad T100 thermal cycler using the following program: 1h at 50ºC, 2 min at 95ºC and 25 cycles of 15 sec at 95ºC and 4 min at 60ºC. No-RT controls were treated the same way except that SuperscriptIII RT enzyme was substituted by Taq Polymerase (Life Technologies). Pre-amplified samples were diluted 1:5 and run on Fluidigm 96.96 arrays on a Biomark device (Fluidigm) together with the Taqman assays listed in Table S2 and reagents according to manufacturer's instructions. Positive controls (10 and 20 cells) were also included in each 96-well plates. Data analysis was first performed in Fluidigm Real-Time PCR Analysis software, and Ct thresholds were set automatically using the auto detector function. Reactions with Ct>40 were considered negative. This data was then analyzed using the Single Cell Expression Visualizer webtool (http://stemsysbio.bmc.lu.se/SCexV/) (Lang et al., 2015) to generate heatmaps and Principal Component Analysis plots. XenoRNA (Ambion, Life Technologies) was used as a loading control and normalization method. Cells displaying a Ct value >14 in the XenoRNA were excluded from the analysis, as well as control samples.
