# Supplementary material for: Defining the Minimal Factors Required for Erythropoiesis through Direct Lineage Conversion
Source: Cell Rep. 2016 Jun 2;15(11):2550–62. doi: 10.1016/j.celrep.2016.05.027 (PMC4914771; doi:10.1016/j.celrep.2016.05.027)
Supplement: Document S2. Article plus Supplemental Information [file mmc2.pdf]

Cell Reports

Article

Defining the Minimal Factors Required for Erythropoiesis through Direct Lineage Conversion

Graphical Abstract

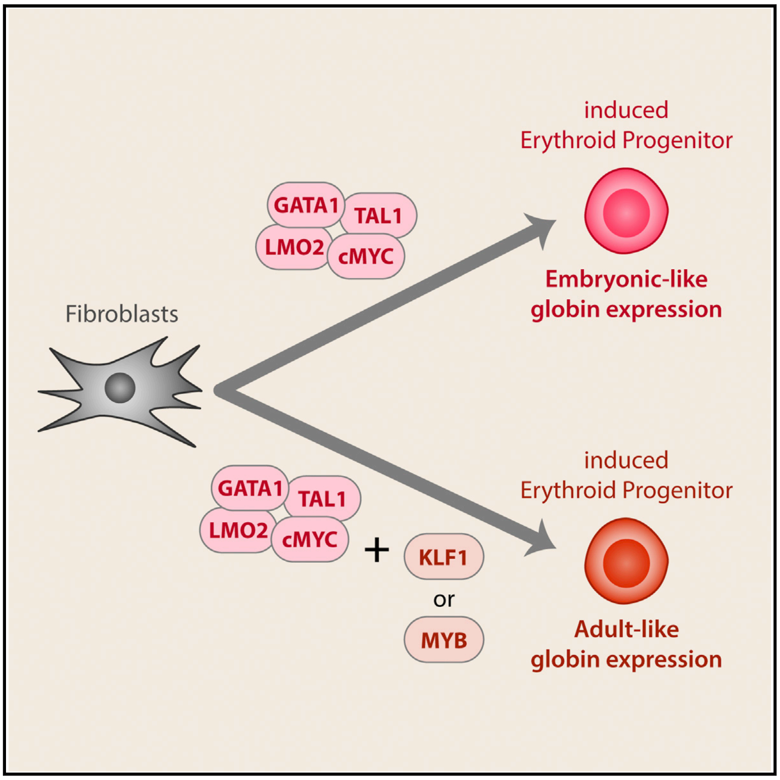

Authors

Sandra Capellera-Garcia, Julian Pulecio, Kishori Dhulipala, ..., Ángel Raya, Vijay G. Sankaran, Johan Flygare

Correspondence

johan.flygare@med.lu.se

In Brief

Capellera-Garcia et al. show that *Gata1*, *Tal1*, *Lmo2*, and *c-Myc* directly convert murine and human fibroblasts into erythroid progenitor/precursor cells. This finding defines a conserved transcriptional program instructing erythroid cell fate in mammals.

Highlights

- *Gata1*, *Tal1*, *Lmo2*, and *c-Myc* reprogram fibroblasts to erythroid progenitors (iEPs)
- iEP gene expression is more similar to that of primitive than definitive erythroblasts
- *Klf1* or *Myb* overexpression induces adult hemoglobin expression in iEPs

Accession Numbers

GSE73344

Capellera-Garcia et al., 2016, Cell Reports *15*, 2550–2562
June 14, 2016 © 2016 The Author(s).
http://dx.doi.org/10.1016/j.celrep.2016.05.027

CellPress

OPEN ACCESS
CellPress

Cell Reports
Article

Defining the Minimal Factors Required for Erythropoiesis through Direct Lineage Conversion

Sandra Capellera-Garcia,[1] Julian Pulecio,[2] Kishori Dhulipala,[1] Kavitha Siva,[1] Violeta Rayon-Estrada,[3] Sofie Singbrant,[1] Mikael N.E. Sommarin,[4] Carl R. Walkley,[5] Shamit Soneji,[4] Göran Karlsson,[4] Ángel Raya,[2,6,7] Vijay G. Sankaran,[8,9] and Johan Flygare[1,*]

[1]Department of Molecular Medicine and Gene Therapy, Lund Stem Cell Center, Lund University, 22184 Lund, Sweden
[2]Center of Regenerative Medicine in Barcelona, Barcelona Biomedical Research Park, Doctor Aiguader 88, 08003 Barcelona, Spain
[3]The Rockefeller University, New York, NY 10065, USA
[4]Division of Molecular Hematology, BMC B12, Lund Stem Cell Center, Lund University, 22184 Lund, Sweden
[5]St. Vincent's Institute of Medical Research and Department of Medicine, St Vincent's Hospital, University of Melbourne, Fitzroy, VIC 3065, Australia
[6]Catalan Institution for Research and Advanced Studies, 08010 Barcelona, Spain
[7]Biomedical Research Networking Center in Bioengineering, Biomaterials and Nanomedicine (CIBER-BBN), 28029 Madrid, Spain
[8]Division of Hematology/Oncology, Boston Children's Hospital and Department of Pediatric Oncology, Dana-Farber Cancer Institute, Harvard Medical School, Boston, MA 02115, USA
[9]Broad Institute of the Massachusetts Institute of Technology and Harvard, Cambridge, MA 02142, USA
*Correspondence: johan.flygare@med.lu.se
http://dx.doi.org/10.1016/j.celrep.2016.05.027

SUMMARY

Erythroid cell commitment and differentiation proceed through activation of a lineage-restricted transcriptional network orchestrated by a group of well characterized genes. However, the minimal set of factors necessary for instructing red blood cell (RBC) development remains undefined. We employed a screen for transcription factors allowing direct lineage reprograming from fibroblasts to induced erythroid progenitors/precursors (iEPs). We show that *Gata1*, *Tal1*, *Lmo2*, and *c-Myc* (GTLM) can rapidly convert murine and human fibroblasts directly to iEPs. The transcriptional signature of murine iEPs resembled mainly that of primitive erythroid progenitors in the yolk sac, whereas addition of *Klf1* or *Myb* to the GTLM cocktail resulted in iEPs with a more adult-type globin expression pattern. Our results demonstrate that direct lineage conversion is a suitable platform for defining and studying the core factors inducing the different waves of erythroid development.

INTRODUCTION

Although several factors are known to participate in the conserved genetic program instructing development of committed erythroid progenitors, the minimal combination of factors required for direct induction of erythroid cell fate remains unknown. The identification of the key players controlling red blood cell (RBC) development is important for understanding basic biology and can be used to study and recapitulate erythropoiesis in vitro as well as to model and develop new therapies for RBC disorders (Tsiftsoglou et al., 2009). Fate decisions in erythropoiesis have been investigated extensively, focusing on lineage-specific transcription factors and cofactors as the main drivers of the process (Cantor and Orkin, 2002; Shivdasani and Orkin, 1996). Genes found to be essential for normal RBC development in mice include *Gata1*, *Zfpm1*, *Lmo2*, *Klf1*, *Myb*, *Tal1*, *Runx1*, and *Ldb1* (Mead et al., 2001; Palis, 2014). However, the factors constituting the core transcriptional machinery that initiates and specifies erythroid cell fate are still unknown.

A major obstacle for defining core transcriptional networks is the difficulty of discriminating "instructive" factors from "permissive" factors. Numerous studies have demonstrated that it is possible to directly convert a mature cell type into another, bypassing the pluripotent state, using a defined set of lineage-instructive transcription factors (Jopling et al., 2011; Takahashi, 2012). This approach, called direct lineage reprogramming, can yield a wide range of clinically relevant cell types, such as neurons, cardiomyocytes, and hepatocytes (Huang et al., 2011; Ieda et al., 2010; Sekiya and Suzuki, 2011; Vierbuchen et al., 2010). Because the converted cells resemble their bona fide counterparts in terms of phenotype and function, direct lineage reprogramming is currently a widely investigated approach for generating defined cell types for regenerative medicine. In contrast to loss-of-function studies, direct reprogramming distinguishes absolutely essential cell fate-inducing factors from merely permissive factors, revealing the master regulators of specific cell lineages (Vierbuchen and Wernig, 2011). Therefore, we reasoned that direct lineage reprogramming is an unambiguous method for defining the core transcriptional machinery directing RBC development.

 © 2016 The Author(s).
This is an open access article under the CC BY license (http://creativecommons.org/licenses/by/4.0/).

Several laboratories have described methods for reprogramming differentiated somatic cells to hematopoietic progenitors with multilineage potential (Batta et al., 2014; Pereira et al., 2013; Riddell et al., 2014; Szabo et al., 2010), whereas others have reported protocols of direct induction to the erythroid lineage starting from B cells (Sadahira et al., 2012) and pluripotent cell sources (Weng and Sheng, 2014). However, none of these studies have shown robust erythroid-restricted fate conversion from non-hematopoietic differentiated somatic cells.

Here we identify the transcription factors *Gata1*, *Tal1*, *Lmo2*, and *c-Myc* (GTLM) as the minimal set of factors for direct conversion of mouse and human fibroblasts into erythroid progenitors. The resulting cells, which we term induced erythroid progenitors/precursors (iEPs), resemble bona fide erythroid cells in terms of morphology, colony-forming capacity, and gene expression. While murine GTLM iEPs express both embryonic and adult globin genes, the addition of *Klf1* or *Myb* induces a switch in globin gene expression to generate iEPs with a predominant definitive-type globin expression pattern. This approach can be used as a model for understanding, controlling, and recapitulating erythroid lineage development and disease.

RESULTS

A Combination of Transcription Factors Induces the Erythroid Fate in Murine Fibroblasts

We hypothesized that overexpression of transcription factors involved in hematopoietic and, specifically, erythroid development in fibroblasts could directly convert these cells into erythroid progenitors or precursors. A retroviral library was created from mouse fetal liver (FL) cDNA expressing the coding region of 63 candidate factors (Table S1). Adult tail tip fibroblasts (TTFs) were derived from erythroid lineage-tracing mice (Heinrich et al., 2004), which express the yellow fluorescent protein (eYFP) from the *Rosa26* locus in all cells that have expressed the erythropoietin receptor (*Epor*, Cre knocked into one allele of the endogenous *Epor* locus) transcript at any stage of their development (Figure 1A). In vivo, the expression of eYFP is first detected in bipotent progenitors of megakaryocytes and erythrocytes (pre-MegEs) and is subsequently robustly expressed in erythroid progenitors (Singbrant et al., 2011). Importantly, eYFP was never detected in other hematopoietic lineages or cell types examined. TTF cultures were carefully depleted of hematopoietic cells by magnetic separation using a cocktail of nine hematopoietic antibodies (Experimental Procedures) and passaged at least three times prior to transduction to obtain pure fibroblast cultures. The primary readout for erythroid lineage conversion was the formation of colonies of $eYFP^+$ ($EpoR^+$) round cells.

Through screening multiple combinations of candidate factors, a combination of seven transcription factors was identified (*Nfe2*, *Myb*, *Klf1*, *Gata1*, *Tal1*, *Lmo2*, and *c-Myc*), that, in 8 days, converted TTFs into clusters of round cells displaying an erythroid precursor-like morphology. These clusters were $eYFP^+$ ($EpoR^+$), indicating induction of the erythroid transcriptional program (Figure 1B). Hence, we termed these cells iEPs.

Only Four Factors Are Necessary and Sufficient for iEP Generation

To identify the minimal set of factors required for iEP generation, we performed single factor subtraction experiments. We found that removal of *Gata1*, *Tal1*, *Lmo2*, or c-*Myc* from the factor cocktail completely abrogated iEP formation (Figure 1C; Figure S1). Notably, TTF reprogramming to iEPs was significantly enhanced using only these four factors compared with the initial seven factors (Figure 1C). Thus, we concluded that GTLM factors constitute the minimal set required for iEP generation.

iEPs Exhibit Morphological and Gene Expression Properties of Erythroid Cells

To better characterize iEP emergence, we examined reprogramming at different time points: on day 5, when the first YFP^+ clusters of round cells appeared, and on day 8, when large YFP^+ colonies could be observed (Figure 2A). On day 5, iEPs displayed an erythroid precursor-like morphology, featuring a characteristic central nucleus, coarse chromatin, and blue cytoplasm after May-Grünwald-Giemsa staining (Figure 2B). Some cells also stained weakly positive with benzidine stain and appeared mildly red when pelleted, indicating that the cells contained hemoglobin (Figures 2C and 2D). On day 5, a small fraction of iEPs co-expressed eYFP and the erythroid-specific surface marker Ter119 (Figure 2E). iEPs harvested on day 8 presented a more differentiated erythroid phenotype. They were significantly smaller, accumulated more hemoglobin, and upregulated Ter119 expression (Figures 2B–2F). Very few enucleated reticulocytes were observed, suggesting inefficient enucleation (Figure 2G). Kinetic analysis by flow cytometry revealed that erythroid precursor output was highest on day 6, followed by day 8, with 10.5% ± 4.6% and 6.6% ± 0.5% of live YFP^+ cells co-expressing CD71 and Ter119, respectively (Figure S2A). Furthermore, the pan hematopoietic marker CD45, which is downregulated in erythroid cells, was not expressed at any time during reprogramming. This suggests that GTLM reprogramming is direct and does not involve an intermediate hematopoietic progenitor stage.

As expected from the erythroid flow cytometric profile and cell morphology, day 8 iEPs downregulated the expression of fibroblast-specific genes and upregulated the expression of erythroid cell-specific genes (Figure 2H). We then analyzed the expression of the different globin genes from both the α and β globin clusters, which are differentially expressed throughout development. In the mouse, all globin genes of the α (*Hba-x*, *Hba-a1*, and *Hba-a2*) and the β globin loci (*Hbb-y*, *Hbb-bh1*, *Hbb-b1*, and *Hbb-b2*) are expressed in primitive erythroid cells, whereas only adult globin genes (*Hba-a1*, *Hba-a2*, *Hbb-b1*, and *Hbb-b2*) are expressed in definitive erythroid cells (Kingsley et al., 2006). Day 8 iEP expressed both embryonic and adult globins, with predominant expression of the embryonic types (Figure 2I). Specifically for the β-globin locus, iEPs expressed 50 times more embryonic *Hbb-y* than adult *Hbb-b1*, suggesting that, although adult fibroblasts were used, GTLM induces an erythroid program that is more similar to primitive than definitive erythropoiesis.

Next, we assayed the in vitro differentiation capacity of both day 5 and day 8 iEPs by colony-forming assays. After 8 days

OPEN
ACCESS
CellPress

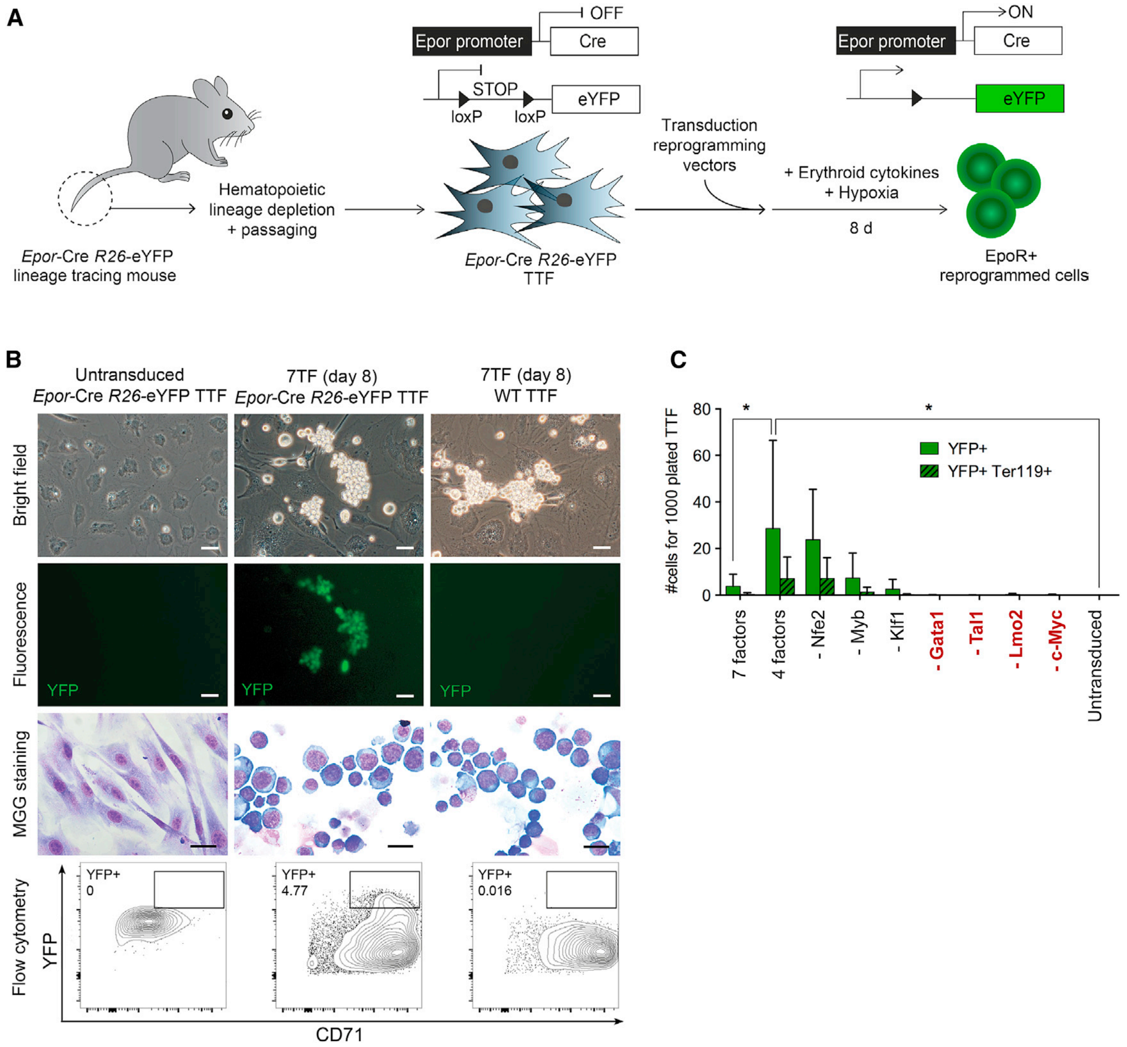

Figure 1. Forced Expression of *Gata1*, *Tal1*, *Lmo2*, and *c-Myc* Reprograms Murine Adult Fibroblasts into Erythroid Progenitors

(A) Experimental design for transcription factor-mediated reprogramming of erythroid reporter (*Epor*-Cre *R26*-eYFP) TTFs to EpoR^{+} reprogrammed cells.

(B) Representative live-cell, bright-field images, fluorescence images, May-Grünwald-Giemsa staining images, and flow cytometry plots of untransduced TTFs and 7TF-iEPs generated from *Epor*-Cre *R26*-eYFP TTFs and WT TTFs on day 8. Scale bars, 20 μm.

(C) Numbers of YFP^{+} and YFP^{+}Ter119^{+} total live cells after removal of individual TFs from the 7TF pool on day 8. Data are presented as mean ± SD (n = 2–3). *$p \leq 0.05$.

See also Figure S1.

in methylcellulose supplemented with human Erythropoietin (hEPO), murine stem cell factor (mSCF), and dexamethasone, iEPs formed two types of colonies: distinctly red (red iEP) and not visibly red (non-red iEP) (Figure 2J). Although cells from red iEP colonies displayed erythroblast morphology, cells from non-red colonies were irregular, had a large deep blue and granular cytoplasm, and did not resemble erythroid cells (Figure 2J). Of the day 5 iEPs, approximately 1 in 1,000 formed red colonies, suggesting that most cells reprogrammed to a more differentiated erythroid cell state without colony-forming ability. This ratio was reduced in day 8 iEPs, which could be explained by iEPs undergoing differentiation from days 5–8 and/or the pMX vectors suffering silencing over time (Figure S2B). In parallel, we asked whether different stoichiometric ratios of GTLM factors could improve the iEPs' erythroid colony output. Interestingly, day 5 iEPs generated with GTLM in a 2:1:1:1 ratio (D5-iEP+G) gave rise to red colonies almost exclusively, whereas day 5 iEPs generated with GTLM in a 1:1:1:2 ratio (D5-iEP+M) generated almost only non-red colonies (Figure 2K). Increasing the ratio of *Tal1* or *Lmo2* had no significant effect. These data imply that optimizing the stoichiometry of the reprogramming factors can further enhance the erythroid output.

In addition to adult tail tip fibroblasts, iEPs could also be generated from murine embryonic fibroblasts (Figures S2C–S2E), demonstrating that the GTLM factors can reprogram fibroblasts from other origins. GTLM induction of the erythroid fate is

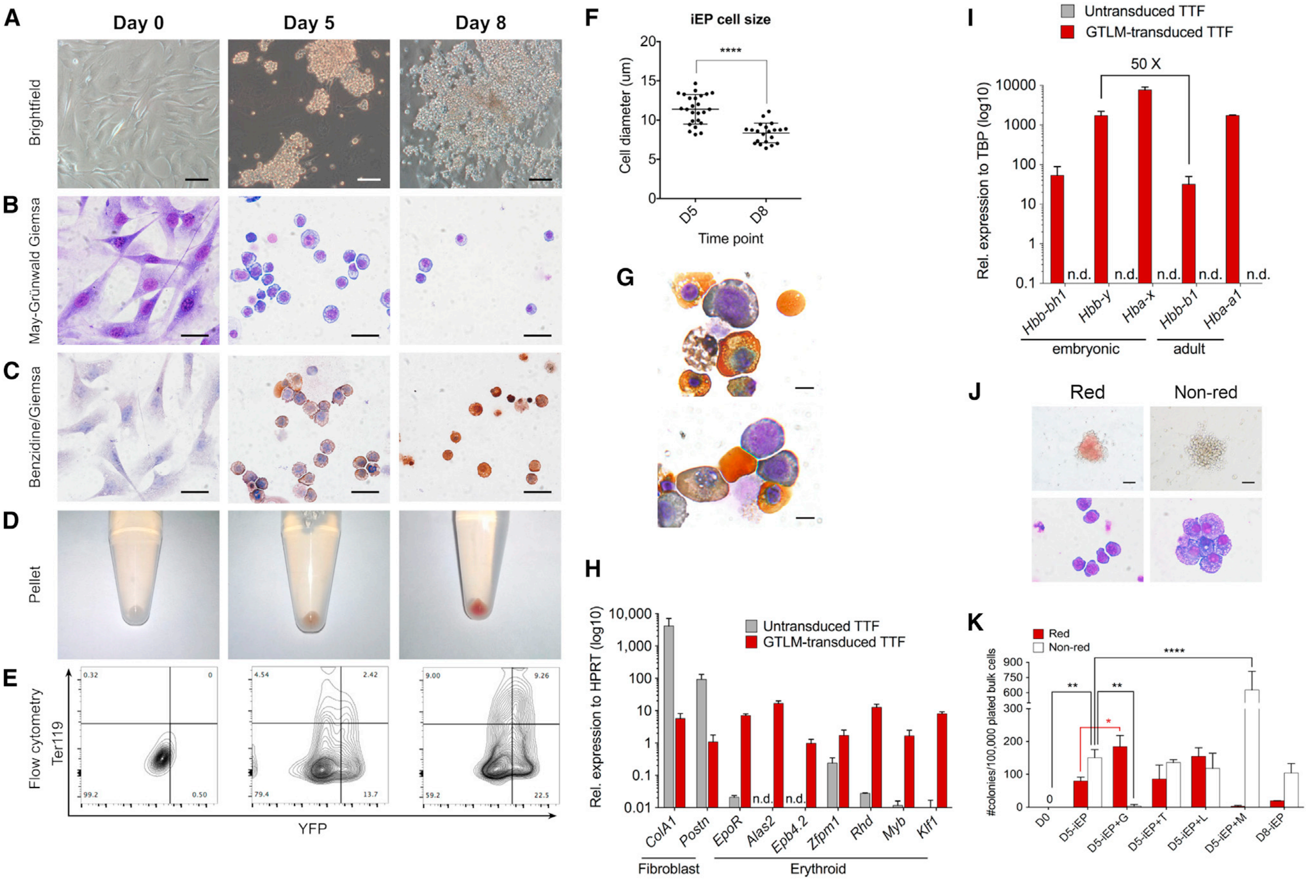

Figure 2. Induced Erythroid Progenitors Exhibit Properties of Bona Fide Erythroid Cells

(A–E) Time course of iEP generation of untransduced TTFs (day 0) and bulk GTLM-transduced TTFs on days 5 and 8 (representative of n = 2–3). Transdifferentiation was evaluated by (A) live-cell, bright-field images of single wells (scale bar, 50 μm); (B) May-Grünwald-Giemsa staining cytospin (scale bar, 20 μm); (C) benzidine/Giemsa staining cytospin (scale bar, 20 μm); (D) macroscopic inspection of cell pellets; and (E) representative flow cytometry plots showing YFP/Ter119 expression.

(F) Cell diameter of iEPs harvested on days 5 and 8 measured by CellSens Standard 1.6 software from several cytospin slides, showing a decrease in cell size on day 8. Data are presented as mean ± SD (n = 21–25). ****$p \leq 0.0001$.

(G) Representative high-resolution benzidine/Giemsa cytospin images of GTLM-transduced TTFs on day 8. Scale bar, 5 μm.

(H and I) Relative mRNA expression of (H) relevant erythroid and fibroblast-specific genes and (I) globin genes in bulk GTLM-transduced TTFs (red columns) versus untransduced TTF (gray columns) on day 8, determined by qPCR. Data are presented as mean ± SD (n = 4–6 for iEPs, n = 2 for untransduced TTFs).

(J) Representative bright-field and May-Grünwald-Giemsa cytospin images of iEP-derived red and non-red colonies.

(K) Colony counts generated from plated untransduced TTFs, bulk day 5 iEPs, bulk day 8 iEPs and bulk day 5 iEPs generated by doubling the ratio of each of the GTLM factors. Scale bars, 50 μm (colony images) and 20 μm (cytospin images). Data are presented as mean ± SD (n = 3). **$p \leq 0.001$; ****$p \leq 0.0001$.

See also Figure S2.

thus a rapid and direct process, yielding erythroblast-like cells with bona fide properties.

iEP-Derived Red Colonies Display a Gene Expression Signature Similar to Bona Fide Burst-Forming Unit-Erythroid Colony Cells

To characterize the reprogrammed cells at the molecular level, we performed global gene expression profiling comparing iEPs with bona fide erythroid progenitors and TTFs. To obtain RNA from pure cell populations, burst-forming unit-Erythroid (BFU-E) colony-forming assays were performed on day 5 iEPs as well as on mouse embryonic day (E) 14.5 FL and adult bone marrow (BM) cells. iEP-derived red and non-red colonies were picked separately, and untransduced TTFs were collected as controls. Unsupervised hierarchical clustering revealed that iEP-derived red colonies (red iEPs) clustered together with primary BFU-Es (FL colony and BM colony) (Figure 3A), indicating that their overall transcriptome is more similar to bona fide erythroid progenitors than to their starting fibroblast cell type. Genes differentially expressed more than 2-fold were mined for significantly overrepresented functional categories using the annotation tool DAVID (Database for Annotation, Visualization and Integrated Discovery) (Huang et al., 2009a, 2009b), demonstrating that genes induced in red iEPs were significantly associated with gene ontology (GO) terms relating to hematopoiesis, erythrocyte function, and development (Figure 3B). On the

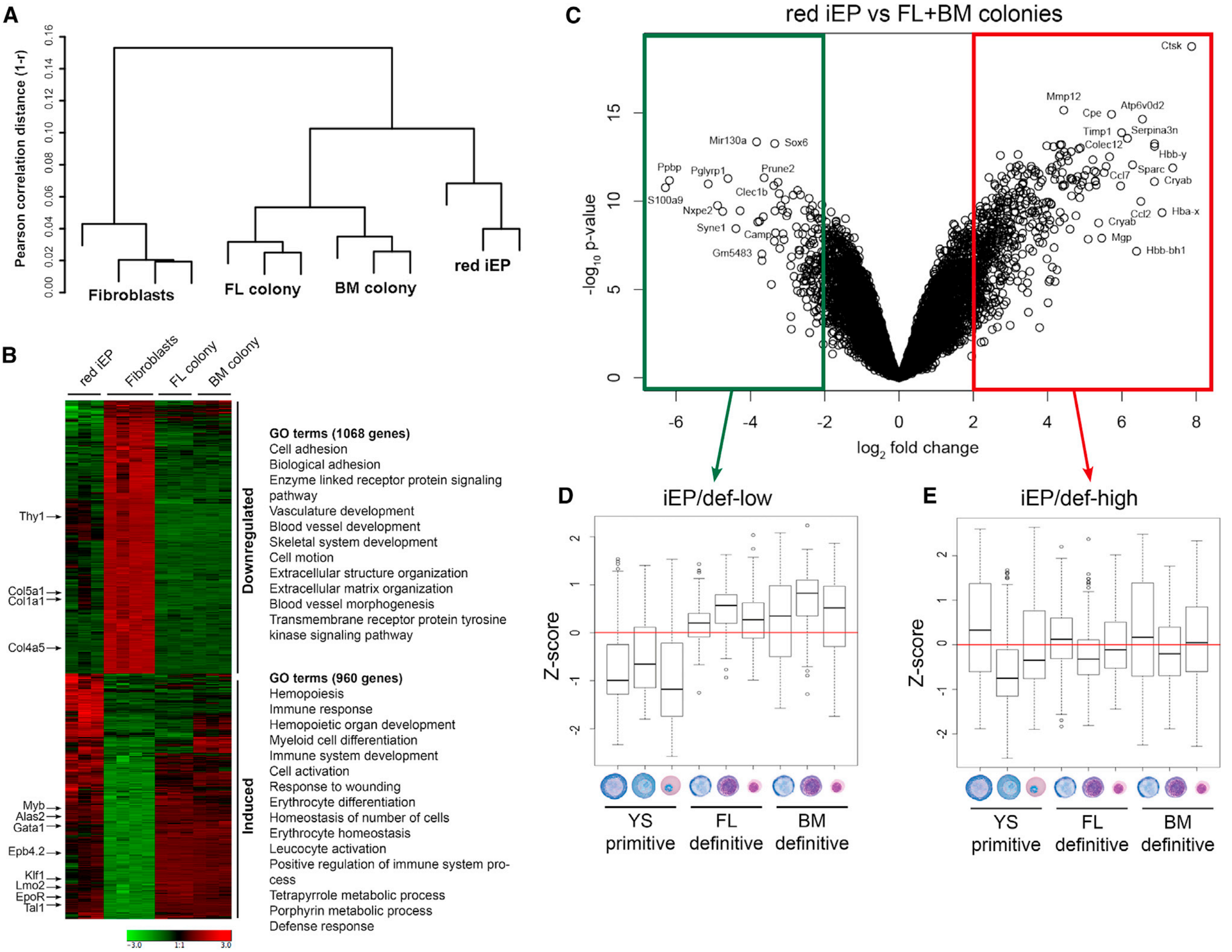

Figure 3. iEP-Derived Red Colonies Retain a Primitive Erythroblast Expression Signature

(A) Unsupervised clustering of samples included in microarray analysis: cultured TTFs (fibroblasts), E14.5 fetal liver BFU-Es (FL colony), adult bone marrow BFU-Es (BM colony), and iEP-derived red colonies (red iEP).

(B) Heatmap depicting genes with ± 2 $\log_2$ fold differential expression between red iEPs and fibroblasts. Downregulated and induced gene clusters are highlighted with their most representative GO terms ($p \leq 10^{-10}$) according to DAVID (Huang et al., 2009a, 2009b).

(C) Volcano plot presenting differentially expressed genes between red iEPs and bona fide BFU-Es (FL and BM BFU-Es). The $\log_2$ fold difference is plotted on the x axis, and the p value-adjusted significance is plotted on the y axis ($-\log_{10}$ scale).

(D and E) Genes with at least 4-fold higher expression in bona fide BFU-Es than in red iEPs (iEP/def-low) and genes with at least 4-fold higher expression in red iEPs than in bona fide BFU-Es (iEP/def-high) were searched in the publicly available Erythron database (Kingsley et al., 2013). Box plots show the average expression of selected iEP/def-low (D) and iEP/def-high (E) genes in YS primitive, FL-definitive and BM-definitive erythroid cells at different stages of maturation. Pictures were taken with permission from Erythron DB (EMBL-EBI: E-MTAB-1035).

See also Figure S3.

contrary, genes downregulated in red iEPs compared with starting fibroblasts were significantly associated with fibroblast-like function, such as "extracellular matrix organization," consistent with the vast inactivation of the fibroblast gene expression program. Taken together, these data demonstrate that red iEP-derived colonies show large-scale downregulation of the fibroblast-specific program and extensive activation of genes specific to the erythroid lineage, indicating that GTLM factors are sufficient to trigger global transcriptional modeling toward the erythroid lineage.

iEP-Derived Non-red Colonies Show Incomplete Reprogramming

To characterize the iEP-derived non-red colonies, we analyzed the differentially expressed genes between red iEPs and non-red iEPs (Figure S3). We found that non-red iEPs lacked induction of genes associated with terminal erythropoiesis, such as globins and genes necessary for heme production (Figure S3A). Moreover, the expression levels of *Gata1*, *Tal1*, and *Lmo2* were 5.6-, 5.2-, and 1.7-fold higher in red iEPs than in non-red iEPs, respectively (Figure S3B). This suggests that non-red iEP

OPEN
ACCESS
CellPress

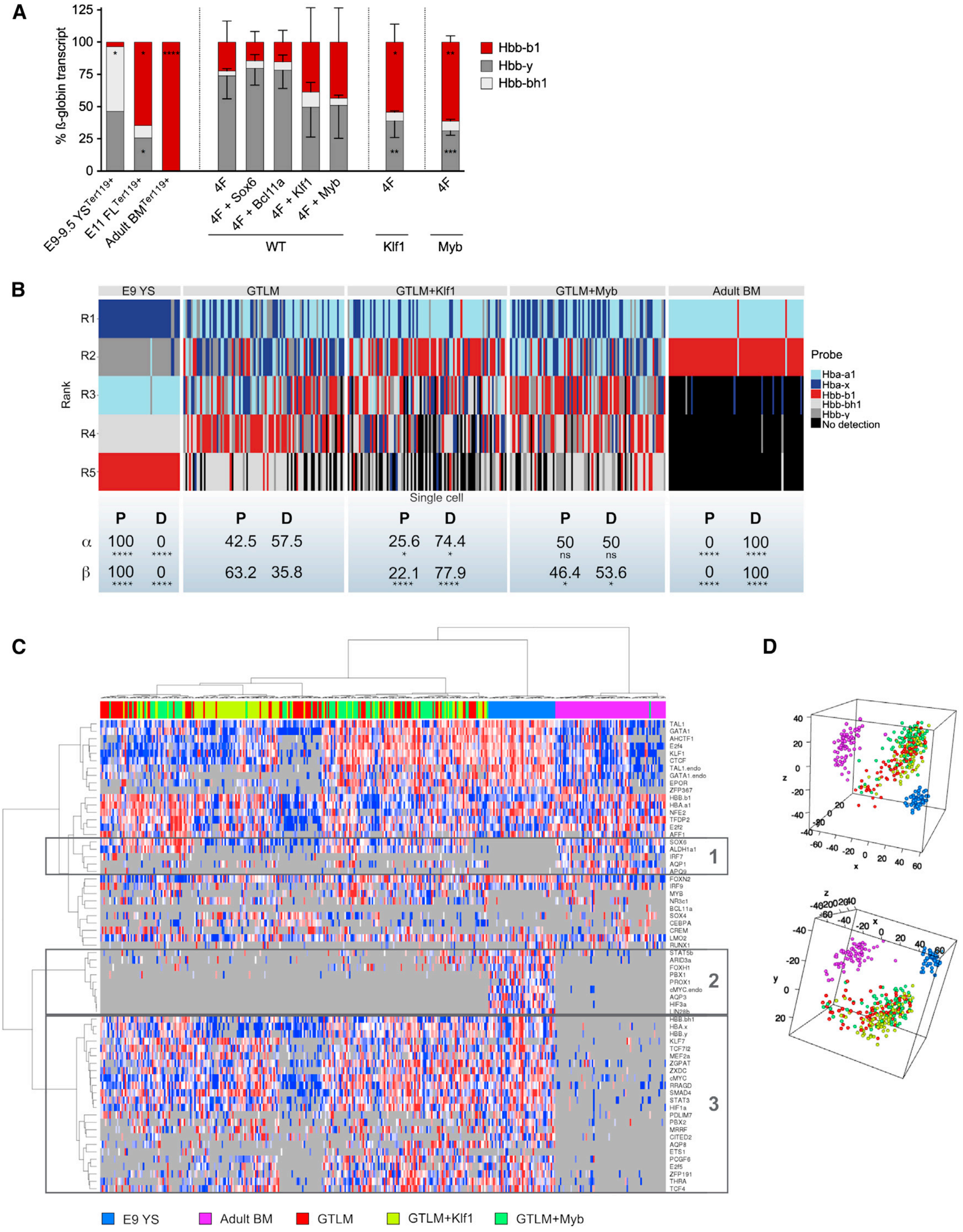

Figure 4. ***Klf1*** **and** ***Myb*** **Enhance Adult Hemoglobin Expression in Single iEPs**

(A) Percentage of embryonic (Hbb-bh1 and Hbb-y) and adult (Hbb-b1) globin transcripts under different test conditions on day 8, determined by qPCR. WT samples denote wild-type TTFs transduced simultaneously with GTLM factors (4F) plus an additional factor. Klf1 and Myb samples denote TTFs with constitutive expression of *Klf1* and *Myb*, respectively, transduced with GTLM factors. Bulk WT GTLM-transduced TTF, Ter119$^+$ cells from E9-9.5 yolk sacs, Ter119$^+$ cells from E11 fetal livers, and Ter119$^+$ cells from adult bone marrow were used as controls. Data are presented as mean ± SD (n = 3–7 for iEP samples, n = 1 for bona fide cells). *p ≤ 0.05; **p ≤ 0.01; ***p ≤ 0.001; ****p ≤ 0.0001.

(B) Map depicting expression of globin genes in single sorted YFP$^+$ Ter119$^+$ E9 yolk sac cells, adult bone marrow cells, day 8 GTLM-iEPs, day 8 GTLM$^+$Klf1-iEPs, and day 8 GTLM$^+$Myb$^-$ iEPs. Globin genes are ranked by their iCt values (iCt = 50 − Ct) in each single cell from highest (R1) to lowest (R5) (Figure S4B).

(legend continued on next page)

colonies have failed to completely reprogram and generate hemoglobinized cells, possibly as a result of inadequate GTL factor expression levels or factor stoichiometry.

The Gene Expression Profile of iEP-Derived Red Colonies Resembles that of Primitive Erythroblasts

Because red iEPs expressed mainly embryonic globin and did not enucleate efficiently, we asked whether their global gene expression profile resembled primitive rather than definitive erythroid cells. Interestingly, the embryonic globin genes (*Hbb-y*, *Hba-x*, and *Hbb-bh1*) were within the top seven differentially expressed genes between red iEPs and bona fide definitive FL and BM BFU-Es (Figure 3C), confirming that this distinctive feature of the primitive erythroid transcriptional program was active in iEPs compared with definitive erythroid cells. Next, we analyzed genes that were at least 4-fold more expressed in bona fide BFU-Es than in red iEPs (iEP/def-low) and genes that were at least 4-fold more expressed in red iEPs than in bona fide BFU-Es (iEP/def-high) and inspected their behavior in data within the Erythron database, which contains global gene expression profiles from primitive, fetal definitive, and adult definitive erythroid cells in mice (Kingsley et al., 2013). We found that iEP/def-low genes in general had lower expression in primitive erythroid cells from the yolk sac compared with the definitive erythroid cells from FL and BM, suggesting that iEPs retain an expression signature similar to that of primitive erythroblasts (Figure 3D). Regarding the iEP/def-high gene set, we did not observe a correlation with the yolk sac profile, although the three embryonic globin genes were among the top differentially expressed genes in this list (Figure 3E). This suggests that bulk iEPs primarily exhibit a molecular signature that is more comparable with a primitive than definitive erythroid program, possibly because additional factors are necessary to enable definitive erythropoiesis. These findings also raise the question of whether individual iEP clones are different so that some are close to primitive erythroid cells, whereas other clones are more similar to definitive erythroid cells.

Induction of Adult Globin Expression by *Klf1* and *Myb*

We next asked whether the overexpression of additional factors could increase the expression of adult hemoglobin in iEPs at the expense of the embryonic globins. This would identify key regulators of hemoglobin expression in iEP as well as improve the potential of these cells for therapeutic applications, as highlighted by Trakarnsanga et al. (2014). We evaluated the transcription factors *Sox6*, *Bcl11a*, *Klf1*, and *Myb*, all previously identified to directly or indirectly downregulate the expression of embryonic and fetal globin genes (Sankaran et al., 2009, 2011; Yi et al., 2006; Zhou et al., 2010). When overexpressing the GTLM factors (4F) together with a single "switching" factor in wild-type (WT) TTF, only *Klf1* and *Myb* increased the expression ratio of adult *Hbb-b1* over embryonic *Hbb-y* compared with the 4F alone, although differences were not statistically significant (Figure 4A).

To investigate whether constitutive expression of switching factors could reinforce this trend, we cloned *Klf1* and *Myb* independently into retroviral vectors including a blasticidin selection gene and generated TTF cell cultures with constitutive expression of *Klf1* or *Myb* (Klf1 and Myb). iEP generated from these fibroblasts presented a reversed globin expression pattern and predominantly expressed adult *Hbb-b1* globin (54.3% ± 13.9% for Klf1 and 61.3% ± 4.9% for Myb). This expression pattern is similar to that of Ter119$^+$ cells harvested from E11 FL, where we still detected *Hbb-y* expression, possibly because of the presence of residual primitive erythroblasts (Figure 4A; McGrath et al., 2011). Thus, the persistence of *Hbb-y* in *Klf1*- and *Myb*-overexpressing cells could reflect the presence of clones with incomplete reprogramming to a definitive-type regulation of globin genes.

Next we investigated the clonal identity and heterogeneity of iEPs and tested whether the addition of *Klf1* and *Myb* generated clones with a more definitive erythroid phenotype overall or merely affected globin expression. We sorted single YFP$^+$ Ter119$^+$ iEPs that were generated from either *Epor* reporter TTF or *Epor* reporter TTF with constitutive expression of *Klf1* or *Myb* and performed single-cell qRT-PCR (Figure S4A). As for the globin genes, we ranked them from highest (R1) to lowest (R5) based on the inverse Ct value (50 – Ct = iCt) of each gene in each single cell (Figure S4B; Figure 4B). GTLM-iEPs expressed both embryonic and adult globins at the single-cell level, indicating that reprogramming generates iEPs with a mixture of globins (Figure 4B). Consistent with our previous observation, *Klf1* and *Myb* overexpression increased the frequency of single cells with a "definitive" globin expression pattern. Of note, *Klf1* affected the expression of both the α and β loci, whereas *Myb* only modified the β locus (Figure 4B). However, not all *Klf1*- and *Myb*-overexpressing cells displayed a definitive-type regulation of globin genes, denoting clonal heterogeneity in reprogramming and globin loci regulation.

Then we analyzed the expression of primitive-specific and definitive-specific genes in the three iEP subsets. We selected transcriptional regulators and other genes that were previously described to be differentially expressed between primitive and adult erythroblasts, regardless of their differentiation stage (see Table S5 in Kingsley et al., 2013). Unsupervised hierarchical clustering revealed that iEPs clustered together with primitive yolk sac erythroid cells (E9 YS), indicating that, based on the selected genes, iEPs are more similar to primitive than definitive erythroid cells (Figure 4C). The clustering did not change when globin and reprogramming genes were removed from the analysis (data not shown). Closer examination of gene groups

Percentages of single cells in each group displaying a "primitive" (P) or definitive (D) globin expression pattern are shown below. P if $iCt^{Hba\text{-}a1} < iCt^{Hba\text{-}x}$ for the α locus or $iCt^{Hbb\text{-}b1} < iCt^{Hbb\text{-}y}$ for the β locus; D if $iCt^{Hba\text{-}a1} > iCt^{Hba\text{-}x}$ for the α locus or $iCt^{Hbb\text{-}b1} > iCt^{Hbb\text{-}y}$ for the β locus. *$p \leq 0.05$; ****$p \leq 0.0001$.

(C) Heatmap depicting the expression of 64 genes selected for single-cell qRT-PCR in sorted YFP$^+$ Ter119$^+$ E9 yolk sac cells (blue), adult bone marrow (purple), day 8 GTLM-iEPs (red), day 8 GTLM$^+$Klf1-iEPs (yellow), and day 8 GTLM$^+$Myb$^-$ iEPs (green).

(D) Principal component analysis. Shown are three-dimensional plots of five sample sets shown from two different points of view. Sample color labeling is consistent with Figure 4C.

See also Figure S4.

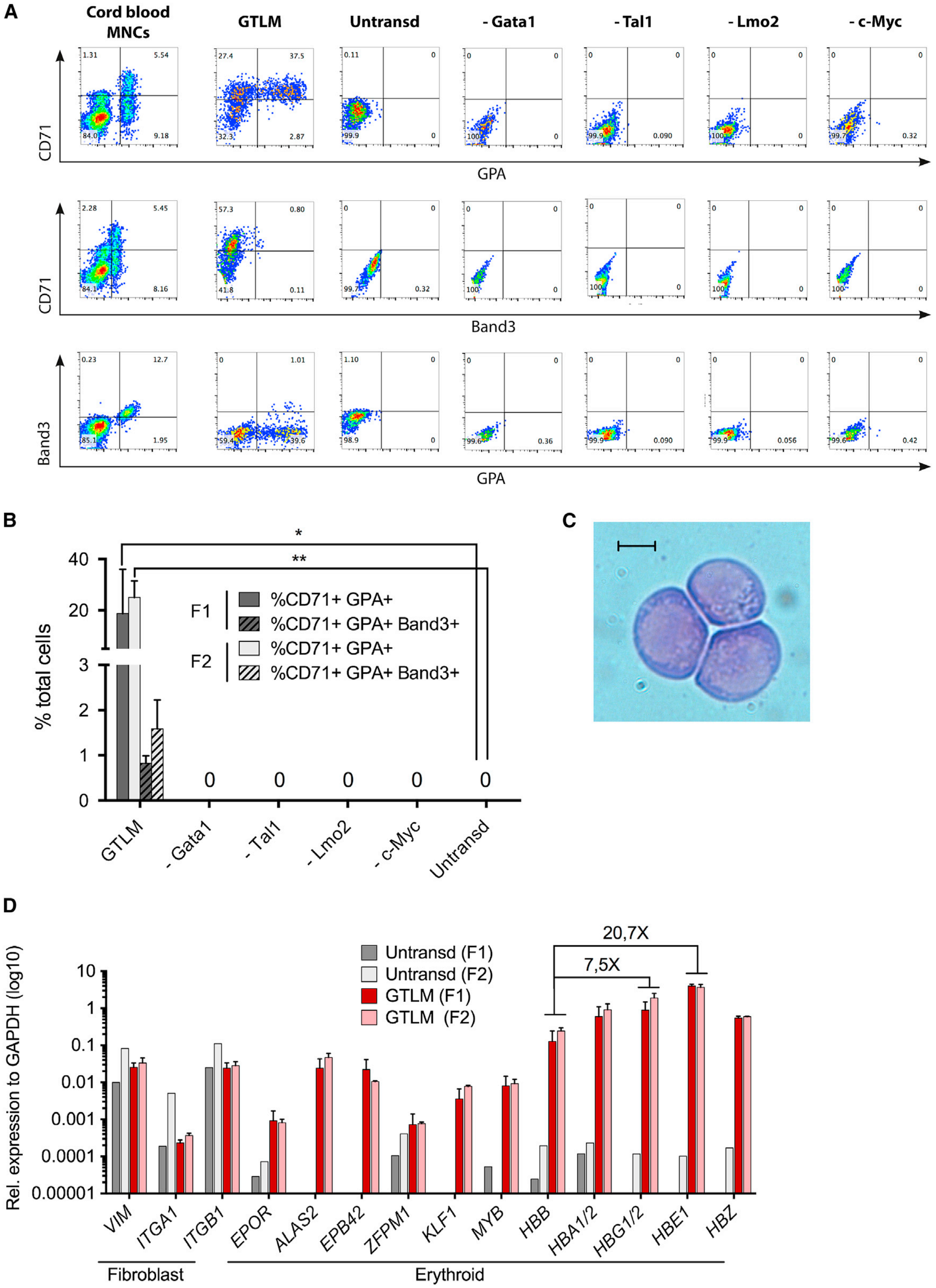

Figure 5. Induction of Erythroid Progenitors from Human Foreskin Fibroblasts by *Gata1*, *Tal1*, *Lmo2*, and *c-Myc*

(A) Representative flow cytometry plots showing CD71, Glycophorin A (GlyA), and Band3 expression in bulk GTLM-transduced HFFs on day 12. Cord blood mononuclear cells and factor minus one combinations were used as controls. The plots shown belong to fibroblast line F1 only (n = 2–3).

(legend continued on next page)

revealed that single iEPs expressed some definitive-specific genes, such as *Sox6* and *Aldh1a1* (cluster 1), as well as some primitive-specific genes, such as *Cited2* and *Rragd* (cluster 3). There was also a group of primitive-specific genes, such as *Lin28b* and *Aqp3*, whose expression was clearly suppressed in iEPs (cluster 2). Furthermore, the addition of *Klf1* or *Myb* did not clearly change the expression of primitive- or definitive-specific genes compared with GTLM alone (Figure 4C). In conclusion, single-cell qRT-PCR demonstrates that iEP clones possess an expression profile reflective of a mixture between primitive and definitive erythropoiesis (Figure 4D) and that *Klf1* and *Myb* do not induce a switch from a generally primitive to a definitive gene expression program but, instead, mainly act as globin switching factors in iEPs by increasing adult hemoglobin expression at the expense of embryonic globins.

Human iEPs Generated by Forced Expression of GTLM

To determine whether GTLM factors could induce the erythroid cell fate in the human setting, we transduced primary cultures of human foreskin fibroblasts (HFFs) from two independent sources with the murine versions of the four factors (Experimental Procedures), and evaluated erythroid reprogramming 12 days thereafter. GTLM-transduced HFFs gave rise to clusters of round cells that co-expressed CD71 and Glycophorin A (GPA), indicating the presence of early erythroid precursor-like cells (Figure 5A; Figure S5). 7.7% ± 5.9% of $CD71^+$ GPA^+ cells were also positive for Band 3, a membrane glycoprotein expressed in late erythroid precursors and mature RBCs (Chen et al., 2009; Figures 5A and 5B; Figure S5). Consistent with the results in the murine system, reprogramming was completely abrogated when one of the GTLM factors was removed, demonstrating that all four are necessary to also induce the erythroid fate in human fibroblasts (Figures 5A and 5B). Bulk human iEPs displayed a round morphology, deep blue cytoplasm, and coarse chromatin, features of early erythroid precursors (Figure 5C). Last, gene expression analysis by qPCR revealed that GTLM factors robustly induced the coordinated expression of several erythroid cell-specific genes, including embryonic (*HBE1*), fetal (*HBG1/2*), and adult hemoglobins (*HBB* and *HBA1/2*) (Figure 5D). As observed in the murine setting, human iEPs expressed 7.5 times more fetal *HBG1/2* and 20.7 times more *HBE1* than adult *HBB*, supporting the previous finding that GTLM induces an embryonic erythroid program. Fibroblast-specific genes were less effectively downregulated upon reprogramming than in mouse TTFs, which is likely reflective of the cellular heterogeneity of the bulk populations analyzed.

Altogether, we provide proof-of-principle evidence that iEPs can be generated from human fibroblasts by overexpression of GTLM, reproducing the findings in the murine setting and underscoring a conserved transcriptional program instructing the erythroid cell fate in mammalian cells.

DISCUSSION

The identification of the minimal set of factors required to instruct erythroid lineage fate could provide a strategy to study and recapitulate erythropoiesis in vitro for medical purposes. We show that murine and human fibroblasts can be rapidly and directly converted into erythroid progenitor/precursor cells by forced expression of *Gata1*, *Tal1*, *Lmo2*, and *c-Myc*. iEPs exhibit properties of bona fide erythroid cells, such as morphology, gene expression, and colony-forming capacity, suggesting that GTLM constitute the core network of the erythroid program capable of orchestrating the battery of factors necessary for normal RBC development.

In erythroid cells, GATA1 and TAL1 are known to assemble within multimeric protein complexes, also including the adaptor molecules LMO2 and LDB1 (Osada et al., 1997) and reviewed by Love et al. (2014). These complexes show widespread binding at erythroid genes and erythroid enhancer elements, functioning as primary mediators of global erythroid gene activation (Li et al., 2013). In addition, the ability of LDB1 protein complexes to oligomerize facilitates long-range associations between promoters and enhancers, which is essential for *β-globin*, *Myb*, and *Epb4.2* gene expression (Song et al., 2007). *Ldb1* is already expressed in fibroblasts (Figure S3D), which likely explains why it is not additionally necessary for iEP generation. Thus, our results suggest that the key event leading to induction of the erythroid fate is the assembly of the GATA1/TAL1/LMO2/LDB1 complex.

The role of *c-Myc* in iEP generation is more ambiguous because its protein is not known to interact with GATA1, TAL1, or LMO2. *c-Myc* is one of the of the four transcription factors used to originally generate induced pluripotent stem cells (iPSCs) from fibroblasts (Takahashi and Yamanaka, 2006). In that context, *c-Myc* has been shown to enhance the early steps of reprograming by repressing fibroblast-specific genes and upregulating the metabolic program of the embryonic state (Sridharan et al., 2009). For iPSC generation, *c-Myc* can be omitted if p53-null fibroblasts are used (Hong et al., 2009). In our system, reprogramming of p53-null fibroblasts greatly enhanced efficiency but did not allow reprogramming without *c-Myc* (data not shown), which suggests that *c-Myc* has a role beyond that shown in iPSC generation. Another indication of *c-Myc*'s erythroid-specific requirement is highlighted by the fact that epiblast-restricted *c-Myc*-null mouse embryos die at E12 from severe anemia (Dubois et al., 2008). Thus, *c-Myc* is likely to have more than just a cell proliferation function during erythroid reprogramming, and future investigation will be needed to determine its precise role in iEP generation. In addition, the

(B) Percentage of $CD71^+GlyA^+$ and $CD71^+GlyA^+Band3^+$ in total live cells for the different transcription factor combinations on day 12. Data are presented as mean ± SD (n = 2–3) for two different fibroblast lines (F1 and F2). $^*p \leq 0.05$; $^{**}p \leq 0.01$.

(C) May-Grünwald-Giemsa staining cytospin image of bulk GTLM-transduced HFFs on day 12. Scale bar, 10 μm.

(D) Relative mRNA expression of relevant human erythroid and fibroblast-specific genes in bulk GTLM-transduced HFFs (red bars) versus untransduced HFFs (gray bars) on day 12, determined by qPCR. Data are presented as mean ± SD (n = 2–3 for hiEPs, n = 1 for untransduced HFFs) for two different fibroblasts lines (F1 and F2).

See also Figure S5.

observation that iEP reprogramming is enhanced by increasing the amount of *Gata1* while increasing *c-Myc*, instead, blocks development of hemoglobinized cells (Figure 2H) demonstrates that GTLM factor stoichiometry is critical and can be further optimized.

Since the advent of iPSC technology in 2006, many laboratories have been screening for factor combinations that can instruct cell fate changes with the main purpose of generating relevant cell types for regenerative medicine. Although non-hematopoietic cells have never been directly reprogrammed to erythroid cells before, different combinations of transcription factors have been shown to reprogram somatic cells to hematopoietic cells, including hematopoietic stem cells and downstream precursor cells (Doulatov et al., 2013; Kulessa et al., 1995; Pereira et al., 2013; Pulecio et al., 2014; Riddell et al., 2014). Recently, Batta et al. (2014) described the generation of hematopoietic progenitors with subsequent erythroid potential by ectopic expression of *Erg*, *Gata2*, *Lmo2*, *Runx1c*, and *Scl* in murine fibroblasts. However, for the factor combinations to be relevant for determining the core transcriptional machinery directing RBC development, it is necessary to reprogram directly to erythroid-restricted progenitor cells. In this regard, the most informative study is a report demonstrating that differentiated murine B cells can be reprogrammed to erythroid-like cells by forced expression of *Gata1*, *Scl*, and *C/EBPα* (Sadahira et al., 2012). *Gata1* and *Scl* were sufficient for reprogramming, but *C/EBPα* enhanced the process by inactivating *Pax6*, a critical transcription factor for B cell differentiation. A second study, focusing on molecular mechanisms controlling the progression from hematopoiesis to erythropoiesis during embryogenesis, reported that a combination of five transcription factors, *Scl*, *Lmo2*, *Gata2*, *Ldb1*, and *E2A*, together with the inhibition of the fibroblast growth factor (FGF) pathway, directly induced erythroid differentiation in the pluripotent chicken epiblast (Weng and Sheng, 2014). Only *Scl* + *Lmo2* and FGF inhibition were sufficient to convert nascent mesoderm cells in later stages of the chicken embryo, suggesting distinct factor requirements depending on the plasticity of the starting population. *Gata1* and *Klf1* could replace *Gata2* in the transcription factor cocktail, but the efficiency was reduced. This study, however, investigated direct erythroid induction from pluripotent cell populations and not terminally differentiated cells, thus illustrating a case of lineage specification rather than transdifferentiation. Taken together, despite the diverse nature of starting cell sources, most of these studies employ the Gata factor family, *Scl/Tal1*, and/or *Lmo2* as conversion factors, validating the essential role of this complex in establishing erythroid lineage identity.

Another common feature of the aforementioned reports and our findings is the detection of embryonic and fetal globins. The inability to express adult globin has also been observed in erythrocytes generated in vitro from pluripotent stem cells or cord blood progenitors, which poses a major barrier to their clinical use (reviewed by Anstee et al. (2012). Together, these observations support the hypothesis that transcription factor-driven reprogramming to blood follows early developmental steps and suggests that the addition of factors mediating the switch to adult globin may be necessary for maturation. Consistent with this, we found that overexpression of *Klf1* or *Myb* changed the globin expression pattern in single iEPs from predominantly embryonic to mainly adult. Although both *Klf1* and *Myb* were found to be dispensable during the initial screening for erythroid fate conversion factors, they were later found to induce adult globin expression. *Myb* is uniquely expressed during definitive erythropoiesis, and its dysregulation is associated with the persistence of embryonic and fetal hemoglobins in Trisomy 13 (Sankaran et al., 2011; Tober et al., 2008). *Klf1*, in contrast, is required for both primitive and definitive erythropoiesis and plays a crucial role in regulating the expression of adult and embryonic globins (Hodge et al., 2006).

Here we show direct and robust red cell fate conversion from a mammalian, non-hematopoietic, differentiated somatic cell. We demonstrate that transcription factor-mediated direct conversion can be employed as an unambiguous method to define the core transcriptional program of a cell type, allowing the distinction of “fate conversion factors” versus “maturation factors.” This method can be applied to define the factors required for human adult erythropoiesis and model disease. Furthermore, the GTLM factors could potentially be used to enhance methods for in vitro production of erythrocytes for personalized transfusion medicine.

EXPERIMENTAL PROCEDURES

Mice

Epor-Cre *R26*-eYFP mice were on a C57Bl/6 background and have been described previously (Heinrich et al., 2004; Singbrant et al., 2011). WT C57BL/6 mice were purchased from Taconic. All animal experiments were carried out in accordance with Lund University’s ethical regulations (Ethical Permit M253-12).

Establishment of Fibroblast Cultures from Mouse Tail Tips

The procedure utilized was a modified version of the protocol by Takahashi and Yamanaka (2006). Tail tips were taken from 6- to 8-week-old mice. The fur was dissected out, and the remaining tissue was manually minced with a sterile scalpel into 1-cm-long pieces and further dissected in the presence of 0.4% trypsin (Thermo Scientific). Tissue pieces were plated on cell culture dishes pre-coated with 0.1% gelatin and incubated in fibroblast medium for 5 days at 37°C, 5% CO_2, 4% O_2. Confluent cultures were passaged twice before hematopoietic lineage depletion and then used for iEP generation. TTF cultures were derived in DMEM supplemented with 15% fetal calf serum (FCS) (Thermo Scientific), 2 mM L-glutamine (Gibco), 1% nonessential amino acids (Gibco), and 1% antibiotics (fibroblast expansion medium [FEX]).

Molecular Cloning and Production of Retrovirus

For the initial screening, coding regions for candidate genes (Table S1) were amplified and cloned into the pMXs retroviral vector backbone using BamHI and NotI restriction sites. The details of the primer sequences are provided in Table S4. For hemoglobin switching experiments, coding regions of *Klf1* and *Myb* were digested out of the pMXs vector using BamHI and SalI and cloned into the pWZL-blast retroviral vector backbone using the same restriction sites. For retrovirus production, 2×10^6 Phoenix GP cells were seeded per 100-mm dish without antibodies. The next day, the medium was changed to pure DMEM, and pMXs plasmids and the Ecopac vector were transfected using FuGENE 6 transfection reagent (Promega). 27 μl of FuGENE 6 was added to 400 μl of DMEM and incubated for 5 min at room temperature. 6 μg of pMXs vector and 3 μg of Ecopac vector were diluted in 25 μl of DMEM and added to the previous mixture, which was incubated for 20 min at room temperature. The DNA/FuGENE 6 mixture was added drop by drop onto the Phoenix GP cells, which were incubated overnight at 37°C,

5% CO_2. The next day, the medium was changed to DMEM + 20% FCS. Viral supernatants were harvested 48 hr after transfection, filtered through a 0.45-μm filter, and frozen for later use.

iEP Generation

Mouse iEP Generation

TTFs were seeded at 1×10^4 cells/cm^2 on 0.1% gelatin pre-coated dishes and infected 24 hr thereafter with non-concentrated, virus-containing supernatants supplemented with 4 μg/ml of Polybrene (Merck Millipore). Virus supernatant and FEX were combined at a ratio of 0.6:1 during transduction. After 4 hr, the medium was changed to serum-free expansion medium (SFEM) (STEMCELL Technologies) supplemented with 1% antibiotics, 100 ng/ml mSCF, 10 ng/ml mIL-3, 2 U/ml recombinant human Erythropoietin (rhEPO), and 100 nM dexamethasone and cultured at 37°C, 5% CO_2 and hypoxia (4% O_2). Emerging murine iEP colonies were scored after 5–8 days after transduction.

Human iEP Generation

For human iEPs, 1×10^5 HFFs were infected three times with several combinations of murine retroviruses at 1,800 rpm for 45 min at 32°C in the presence of 4 μg/ml Polybrene and cultured at 32°C for 12 hr between infections. The culture medium containing the viruses was changed the day thereafter for reprogramming medium (Iscove's modified Delbecco's medium [IMDM], 0.4% albumin, 1% human serum supplemented with 1% antibiotics, insulin-transferring-selenium [ITS] (Gibco), 100 ng/ml hSCF, 10 ng/ml hIL-3, 2 U/ml rhEPO, and 100 nM dexamethasone) and cultured at 37°C, 5%CO_2, and 4% O_2.

Establishment of Mouse Fibroblast Cultures with Constitutive Expression of *Klf1* or *Myb*

TTFs were seeded and transduced with pWZL-blast-*Klf1* or pWZL-blast-*Myb* as described under iEP Generation. 48 hr after transduction, cells were passaged and cultured in FEX supplemented with 4 μg/ml blasticidin antibiotic (InvivoGen). The whole medium was replaced with fresh antibiotic-containing medium every 3 days. When a population of resistant TTFs was obtained, cells were seeded accordingly and transduced with pMX vectors to generate iEPs. Blasticidin was added to the medium during reprogramming.

Flow Cytometry

Clusters of round cells were collected through gentle pipetting, and untransduced fibroblasts were trypsinized. Cells were resuspended in 100 μl of PBS + 2% FCS and stained for 20 min at 4°C in the dark. The list of antibodies and dilutions can be found in the Supplemental Experimental Procedures. After staining, cells were washed and resuspended in 300 μl of PBS with 2% FCS. To assess cell viability, cells were also stained with 1:100 DAPI. Compensation controls were set with murine bone marrow cells. The analysis of mouse cells was performed using FACSCanto II (Becton Dickinson), and data were analyzed using FlowJo v10 software. The analysis of human cells was performed using a Gallios flow cytometer (Becton Dickinson).

RNA Isolation and qPCR

Total RNA was isolated with the RNAeasy kit (QIAGEN) according to the manufacturer's guidelines. RNA was subjected to cDNA synthesis using Superscript III (Invitrogen). Quantitative PCR analysis was performed in triplicates for each sample and Taqman gene expression assays (Applied Biosystems) or PrimeTime qPCR assays (Integrated DNA Technologies). For each reaction, 10 μl of Taqman master mix, 4 μl of sterile water, and 1 μl of the primer assay were mixed. Then, 5 μl of cDNA (<40 ng/μl) was added to each well. The signal was detected with a 7900HT RT-PCR instrument (Applied Biosystems). The primers used are listed in Tables S2 and S3.

Colony Assay

For colony-forming assays, MethoCult M3236 (STEMCELL Technologies) was used, which was supplemented with 5 U/ml rhEPO, 50 ng/ml mSCF, 100 nM of dexamethasone, and 1% antibiotics. Cells were resuspended in FCS and added to the MethoCult mixture. All experiments were performed in triplicate. Colonies were scored on day 8.

Global Gene Expression Analysis

Total RNA was isolated from red iEP, non-red iEP, FL colony, BM colony, and fibroblasts and hybridized to a Mouse Gene 2.1 ST array (Affymetrix) according to the manufacturer's protocol. The arrays were robust multi-array average (RMA)-normalized, and differentially expressed genes were identified using LIMMA (25605792). The microarray data have been deposited in NCBI's GEO (Edgar et al., 2002) and are accessible through GEO: GSE73344.

Single-Cell qRT-qPCR and Data Analysis

Single YFP$^+$ Ter119$^+$ cells were sorted into 96-well PCR plates (Sarstedt) containing 4 μl of lysis buffer. Next, target-specific pre-amplification was performed using the CellsDirect one-step qRT-PCR kit (Life Technologies). Pre-amplified samples were diluted 1:5 and run on Fluidigm 96.96 arrays on a Biomark device (Fluidigm) together with the Taqman assays listed in Table S2 and reagents according to the manufacturer's instructions. The data were analyzed using the Single Cell Expression Visualizer web tool (http://stemsysbio.bmc.lu.se/SCexV/) (Lang et al., 2015). See the Supplemental Experimental Procedures for details.

Statistical Evaluation

Statistical analyses of all endpoints were performed using unpaired Student's t test (Figure 2F) and two-way ANOVA test (Dunnett correction for multiple comparisons, 95% confidence intervals) (Figures 1C, 2J, 4A and 4B, and 5B) using GraphPad Prism 6.

ACCESSION NUMBERS

The microarray data have been deposited in NCBI's Gene Expression Omnibus (Edgar et al., 2002) and are accessible through GEO: GSE73344.

SUPPLEMENTAL INFORMATION

Supplemental Information includes Supplemental Experimental Procedures, five figures, and four tables and can be found with this article online at http://dx.doi.org/10.1016/j.celrep.2016.05.027.

AUTHOR CONTRIBUTIONS

Conceptualization, S.C.G., K.D., K.S., S. Singbrant, V.G.S., and J.F.; Methodology, S.C.G., K.D., K.S., V.G.S., and J.F.; Software and Formal Analysis, S. Soneji; Investigation – Murine Data, S.C.G., K.D., and K.S.; Investigation – Human Data, J.P. and S.C.G.; Library Generation, V.R.E. and J.F.; Single-Cell qRT-PCR Conceptualization and Analysis, M.S., S.C.G., and G.K., Writing – Original Draft, S.C.G. and J.F.; Writing – Review & Editing, S.C.G., V.G.S., S. Singbrant., K.S., S. Soneji, V.R.E., J.P., A.R., C.W., and J.F.; Funding Acquisition, A.R. and J.F.; Resources, C.W., A.R., and J.F.; Supervision, J.F.

ACKNOWLEDGMENTS

We thank Evelyn Wang and Gregory Hyde (Whitehead Institute, Cambridge, MA) for cloning and Harvey Lodish (Whitehead Institute) for providing many of the plasmids used for generating the retroviral library. We thank Jacob Ulirsch (Broad Institute of MIT and Harvard) for early contributions to the global gene expression analysis. We thank Roger Rönn (Department of Molecular Medicine and Gene Therapy) for input on the manuscript and Zhi Ma (Lund Stem Cell Center) for flow cytometry assistance. This work was supported by the Ragnar Söderberg Foundation (to J.F.); the Swedish Research Council (to J.F.); Stiftelsen Olle Engkvist Byggmästare (to J.F.); the Swedish Foundation for Strategic Research (to J.F.); Åke Wiberg's Foundation (to J.F.); a Marie Curie integration grant (to J.F.); and grants from MINECO (SAF2012-33526) (to A.R.), ISCIII – FEDER (RETICS RD12/0019/0019, RD12/0019/0034) (to A.R.), Fundació Marató de TV3 (121430) (to A.R.), and AGAUR (2014-SGR-1460, 2014-SGR-1570) (to A.R.). J.P. was partially supported by a Juan de la Cierva postdoctoral fellowship (JCI-2012-15293).

Received: October 13, 2015
Revised: April 5, 2016
Accepted: May 5, 2016
Published: June 2, 2016

REFERENCES

Anstee, D.J., Gampel, A., and Toye, A.M. (2012). Ex-vivo generation of human red cells for transfusion. Curr. Opin. Hematol. *19*, 163–169.

Batta, K., Florkowska, M., Kouskoff, V., and Lacaud, G. (2014). Direct reprogramming of murine fibroblasts to hematopoietic progenitor cells. Cell Rep. *9*, 1871–1884.

Cantor, A.B., and Orkin, S.H. (2002). Transcriptional regulation of erythropoiesis: an affair involving multiple partners. Oncogene *21*, 3368–3376.

Chen, K., Liu, J., Heck, S., Chasis, J.A., An, X., and Mohandas, N. (2009). Resolving the distinct stages in erythroid differentiation based on dynamic changes in membrane protein expression during erythropoiesis. Proc. Natl. Acad. Sci. USA *106*, 17413–17418.

Doulatov, S., Vo, L.T., Chou, S.S., Kim, P.G., Arora, N., Li, H., Hadland, B.K., Bernstein, I.D., Collins, J.J., Zon, L.I., and Daley, G.Q. (2013). Induction of multipotential hematopoietic progenitors from human pluripotent stem cells via respecification of lineage-restricted precursors. Cell Stem Cell *13*, 459–470.

Dubois, N.C., Adolphe, C., Ehninger, A., Wang, R.A., Robertson, E.J., and Trumpp, A. (2008). Placental rescue reveals a sole requirement for c-Myc in embryonic erythroblast survival and hematopoietic stem cell function. Development *135*, 2455–2465.

Edgar, R., Domrachev, M., and Lash, A.E. (2002). Gene Expression Omnibus: NCBI gene expression and hybridization array data repository. Nucleic Acids Res. *30*, 207–210.

Heinrich, A.C., Pelanda, R., and Klingmüller, U. (2004). A mouse model for visualization and conditional mutations in the erythroid lineage. Blood *104*, 659–666.

Hodge, D., Coghill, E., Keys, J., Maguire, T., Hartmann, B., McDowall, A., Weiss, M., Grimmond, S., and Perkins, A. (2006). A global role for EKLF in definitive and primitive erythropoiesis. Blood *107*, 3359–3370.

Hong, H., Takahashi, K., Ichisaka, T., Aoi, T., Kanagawa, O., Nakagawa, M., Okita, K., and Yamanaka, S. (2009). Suppression of induced pluripotent stem cell generation by the p53-p21 pathway. Nature *460*, 1132–1135.

Huang, W., Sherman, B.T., and Lempicki, R.A. (2009a). Bioinformatics enrichment tools: paths toward the comprehensive functional analysis of large gene lists. Nucleic Acids Res. *37*, 1–13.

Huang, W., Sherman, B.T., and Lempicki, R.A. (2009b). Systematic and integrative analysis of large gene lists using DAVID bioinformatics resources. Nat. Protoc. *4*, 44–57.

Huang, P., He, Z., Ji, S., Sun, H., Xiang, D., Liu, C., Hu, Y., Wang, X., and Hui, L. (2011). Induction of functional hepatocyte-like cells from mouse fibroblasts by defined factors. Nature *475*, 386–389.

Ieda, M., Fu, J.D., Delgado-Olguin, P., Vedantham, V., Hayashi, Y., Bruneau, B.G., and Srivastava, D. (2010). Direct reprogramming of fibroblasts into functional cardiomyocytes by defined factors. Cell *142*, 375–386.

Jopling, C., Boue, S., and Izpisua Belmonte, J.C. (2011). Dedifferentiation, transdifferentiation and reprogramming: three routes to regeneration. Nat. Rev. Mol. Cell Biol. *12*, 79–89.

Kingsley, P.D., Malik, J., Emerson, R.L., Bushnell, T.P., McGrath, K.E., Bloedorn, L.A., Bulger, M., and Palis, J. (2006). "Maturational" globin switching in primary primitive erythroid cells. Blood *107*, 1665–1672.

Kingsley, P.D., Greenfest-Allen, E., Frame, J.M., Bushnell, T.P., Malik, J., McGrath, K.E., Stoeckert, C.J., and Palis, J. (2013). Ontogeny of erythroid gene expression. Blood *121*, e5–e13.

Kulessa, H., Frampton, J., and Graf, T. (1995). GATA-1 reprograms avian myelomonocytic cell lines into eosinophils, thromboblasts, and erythroblasts. Genes Dev. *9*, 1250–1262.

Lang, S., Ugale, A., Erlandsson, E., Karlsson, G., Bryder, D., and Soneji, S. (2015). SCExV: a webtool for the analysis and visualisation of single cell qRT-PCR data. BMC Bioinformatics *16*, 320.

Li, L., Freudenberg, J., Cui, K., Dale, R., Song, S.H., Dean, A., Zhao, K., Jothi, R., and Love, P.E. (2013). Ldb1-nucleated transcription complexes function as primary mediators of global erythroid gene activation. Blood *121*, 4575–4585.

Love, P.E., Warzecha, C., and Li, L. (2014). Ldb1 complexes: the new master regulators of erythroid gene transcription. Trends Genet. *30*, 1–9.

McGrath, K.E., Frame, J.M., Fromm, G.J., Koniski, A.D., Kingsley, P.D., Little, J., Bulger, M., and Palis, J. (2011). A transient definitive erythroid lineage with unique regulation of the β-globin locus in the mammalian embryo. Blood *117*, 4600–4608.

Mead, P.E., Deconinck, A.E., Huber, T.L., Orkin, S.H., and Zon, L.I. (2001). Primitive erythropoiesis in the Xenopus embryo: the synergistic role of LMO-2, SCL and GATA-binding proteins. Development *128*, 2301–2308.

Osada, H., Grutz, G.G., Axelson, H., Forster, A., and Rabbitts, T.H. (1997). LIM-only protein Lmo2 forms a protein complex with erythroid transcription factor GATA-1. Leukemia *11* (*Suppl 3*), 307–312.

Palis, J. (2014). Primitive and definitive erythropoiesis in mammals. Front. Physiol. *5*, 3.

Pereira, C.F., Chang, B., Qiu, J., Niu, X., Papatsenko, D., Hendry, C.E., Clark, N.R., Nomura-Kitabayashi, A., Kovacic, J.C., Ma'ayan, A., et al. (2013). Induction of a hemogenic program in mouse fibroblasts. Cell Stem Cell *13*, 205–218.

Pulecio, J., Nivet, E., Sancho-Martinez, I., Vitaloni, M., Guenechea, G., Xia, Y., Kurian, L., Dubova, I., Bueren, J., Laricchia-Robbio, L., and Izpisua Belmonte, J.C. (2014). Conversion of human fibroblasts into monocyte-like progenitor cells. Stem Cells *32*, 2923–2938.

Riddell, J., Gazit, R., Garrison, B.S., Guo, G., Saadatpour, A., Mandal, P.K., Ebina, W., Volchkov, P., Yuan, G.C., Orkin, S.H., and Rossi, D.J. (2014). Reprogramming committed murine blood cells to induced hematopoietic stem cells with defined factors. Cell *157*, 549–564.

Sadahira, K., Fukuchi, Y., Kunimono, H., Sakurai, M., Ikeda, Y., Okamoto, S., and Nakajima, H. (2012). Direct reprogramming of terminally differentiated B cells into erythroid lineage. FEBS Lett. *586*, 3645–3652.

Sankaran, V.G., Xu, J., Ragoczy, T., Ippolito, G.C., Walkley, C.R., Maika, S.D., Fujiwara, Y., Ito, M., Groudine, M., Bender, M.A., et al. (2009). Developmental and species-divergent globin switching are driven by BCL11A. Nature *460*, 1093–1097.

Sankaran, V.G., Menne, T.F., Šćepanović, D., Vergilio, J.A., Ji, P., Kim, J., Thiru, P., Orkin, S.H., Lander, E.S., and Lodish, H.F. (2011). MicroRNA-15a and -16-1 act via MYB to elevate fetal hemoglobin expression in human trisomy 13. Proc. Natl. Acad. Sci. USA *108*, 1519–1524.

Sekiya, S., and Suzuki, A. (2011). Direct conversion of mouse fibroblasts to hepatocyte-like cells by defined factors. Nature *475*, 390–393.

Shivdasani, R.A., and Orkin, S.H. (1996). The transcriptional control of hematopoiesis. Blood *87*, 4025–4039.

Singbrant, S., Russell, M.R., Jovic, T., Liddicoat, B., Izon, D.J., Purton, L.E., Sims, N.A., Martin, T.J., Sankaran, V.G., and Walkley, C.R. (2011). Erythropoietin couples erythropoiesis, B-lymphopoiesis, and bone homeostasis within the bone marrow microenvironment. Blood *117*, 5631–5642.

Song, S.H., Hou, C., and Dean, A. (2007). A positive role for NLI/Ldb1 in long-range beta-globin locus control region function. Mol. Cell *28*, 810–822.

Sridharan, R., Tchieu, J., Mason, M.J., Yachechko, R., Kuoy, E., Horvath, S., Zhou, Q., and Plath, K. (2009). Role of the murine reprogramming factors in the induction of pluripotency. Cell *136*, 364–377.

Szabo, E., Rampalli, S., Risueño, R.M., Schnerch, A., Mitchell, R., Fiebig-Comyn, A., Levadoux-Martin, M., and Bhatia, M. (2010). Direct conversion of human fibroblasts to multilineage blood progenitors. Nature *468*, 521–526.

Takahashi, K. (2012). Cellular reprogramming–lowering gravity on Waddington's epigenetic landscape. J. Cell Sci. *125*, 2553–2560.

Takahashi, K., and Yamanaka, S. (2006). Induction of pluripotent stem cells from mouse embryonic and adult fibroblast cultures by defined factors. Cell *126*, 663–676.

Tober, J., McGrath, K.E., and Palis, J. (2008). Primitive erythropoiesis and megakaryopoiesis in the yolk sac are independent of c-myb. Blood *111*, 2636–2639.

Trakarnsanga, K., Wilson, M.C., Lau, W., Singleton, B.K., Parsons, S.F., Sakuntanaga, P., Kurita, R., Nakamura, Y., Anstee, D.J., and Frayne, J. (2014). Induction of adult levels of β-globin in human erythroid cells that intrinsically express embryonic or fetal globin by transduction with KLF1 and BCL11A-XL. Haematologica *99*, 1677–1685.

Tsiftsoglou, A.S., Vizirianakis, I.S., and Strouboulis, J. (2009). Erythropoiesis: model systems, molecular regulators, and developmental programs. IUBMB Life *61*, 800–830.

Vierbuchen, T., and Wernig, M. (2011). Direct lineage conversions: unnatural but useful? Nat. Biotechnol. *29*, 892–907.

Vierbuchen, T., Ostermeier, A., Pang, Z.P., Kokubu, Y., Südhof, T.C., and Wernig, M. (2010). Direct conversion of fibroblasts to functional neurons by defined factors. Nature *463*, 1035–1041.

Weng, W., and Sheng, G. (2014). Five transcription factors and FGF pathway inhibition efficiently induce erythroid differentiation in the epiblast. Stem Cell Reports *2*, 262–270.

Yi, Z., Cohen-Barak, O., Hagiwara, N., Kingsley, P.D., Fuchs, D.A., Erickson, D.T., Epner, E.M., Palis, J., and Brilliant, M.H. (2006). Sox6 directly silences epsilon globin expression in definitive erythropoiesis. PLoS Genet. *2*, e14.

Zhou, D., Liu, K., Sun, C.W., Pawlik, K.M., and Townes, T.M. (2010). KLF1 regulates BCL11A expression and gamma- to beta-globin gene switching. Nat. Genet. *42*, 742–744.

Cell Reports, Volume *15*

Supplemental Information

Defining the Minimal Factors Required for Erythropoiesis through Direct Lineage Conversion

Sandra Capellera-Garcia, Julian Pulecio, Kishori Dhulipala, Kavitha Siva, Violeta Rayon-Estrada, Sofie Singbrant, Mikael N.E. Sommarin, Carl R. Walkley, Shamit Soneji, Göran Karlsson, Ángel Raya, Vijay G. Sankaran, and Johan Flygare

Figure S1

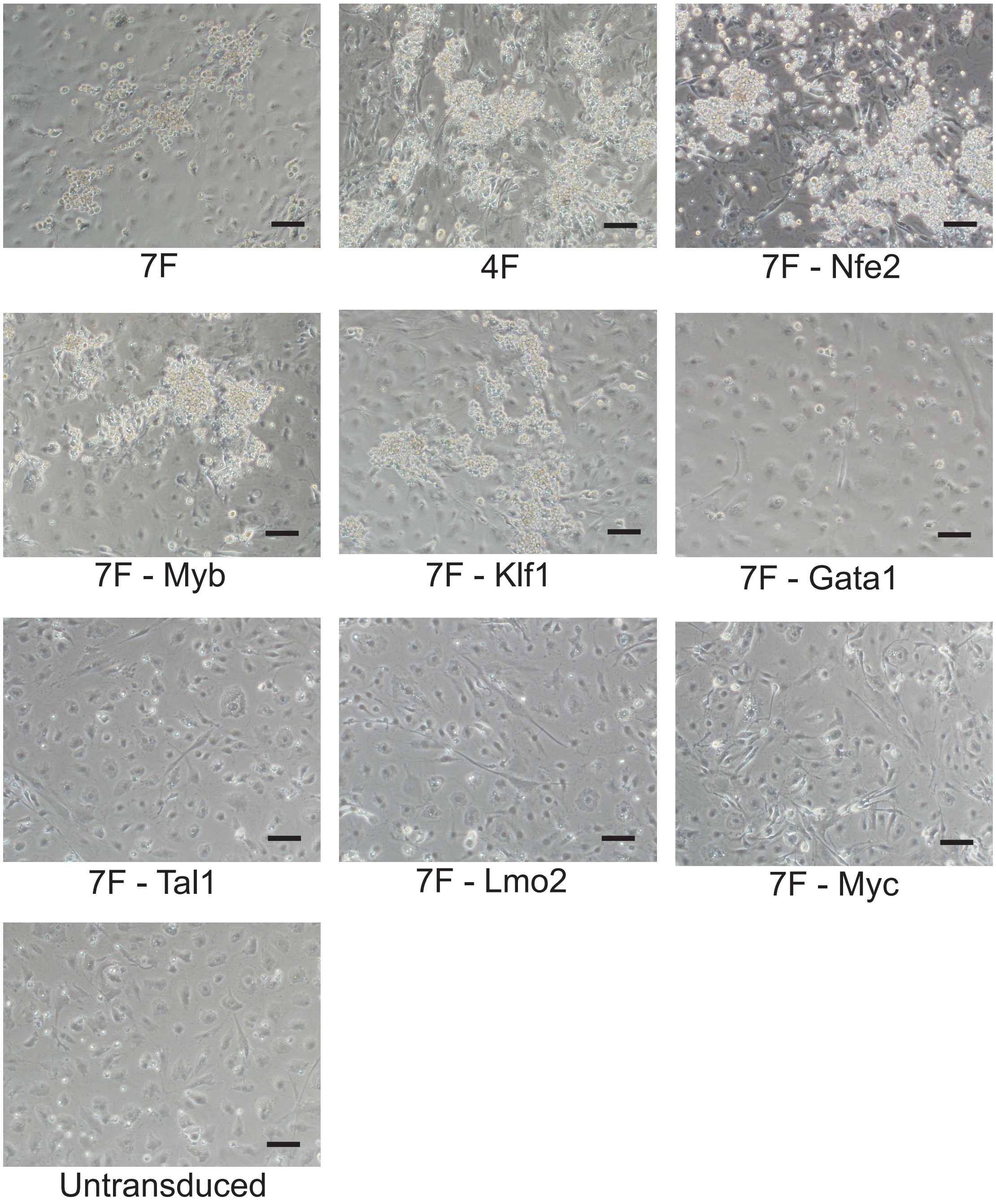
7F
4F
7F - Nfe2
7F - Myb
7F - Klf1
7F - Gata1
7F - Tal1
7F - Lmo2
7F - Myc
Untransduced

Figure S2

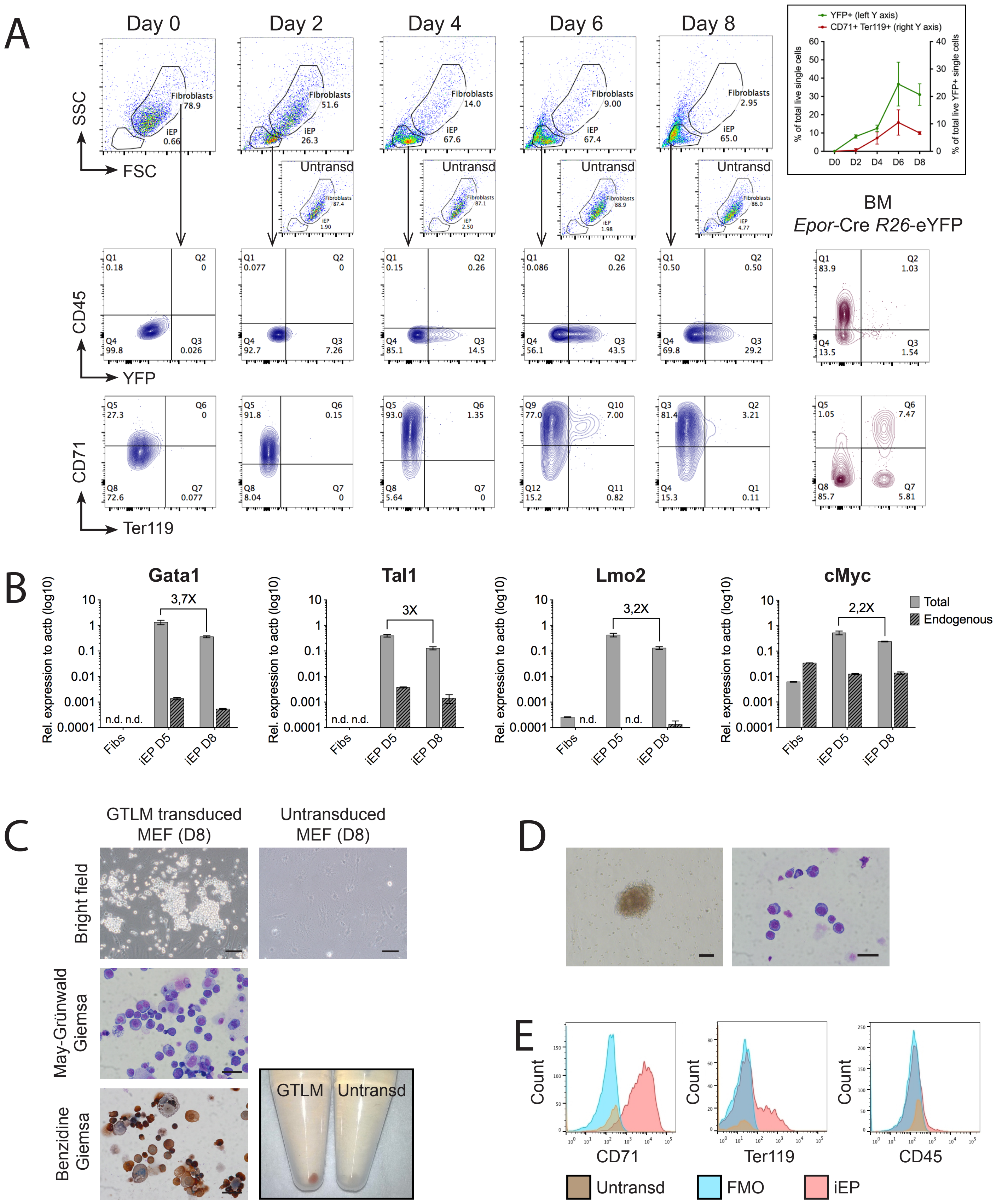
A
Day 0
Day 2
Day 4
Day 6
Day 8
SSC
FSC
Fibroblasts 78.9
iEP 0.66
Fibroblasts 51.6
iEP 26.3
Fibroblasts 14.0
iEP 67.6
Fibroblasts 9.00
iEP 67.4
Fibroblasts 2.95
iEP 65.0
Untransd
YFP+ (left Y axis)
CD71+ Ter119+ (right Y axis)
% of total live single cells
% of total live YFP+ single cells
D0 D2 D4 D6 D8
BM
Epor-Cre R26-eYFP
CD45
YFP
CD71
Ter119
B
Gata1
Tal1
Lmo2
cMyc
3,7X
3X
3,2X
2,2X
Rel. expression to actb (log10)
n.d.
Fibs
iEP D5
iEP D8
Total
Endogenous
C
GTLM transduced MEF (D8)
Untransduced MEF (D8)
Bright field
May-Grünwald Giemsa
Benzidine Giemsa
GTLM
Untransd
D
E
Count
CD71
Ter119
CD45
Untransd
FMO
iEP

Figure S3

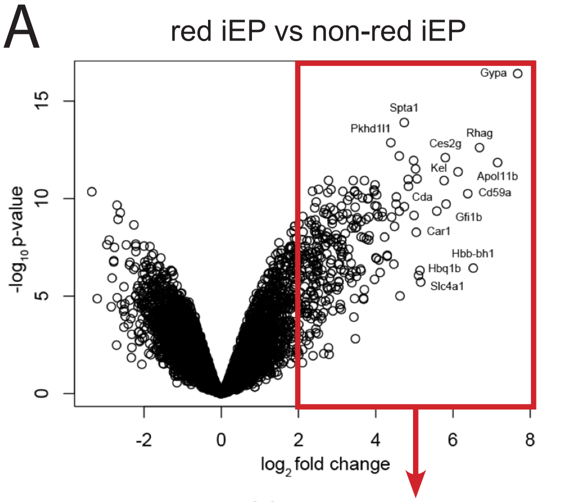

GO terms (417 genes)
Tetrapyrrole biosynthetic process
Porphyrin biosynthetic process
Erythrocyte differentiation
Porphyrin metabolic process
Tetrapyrrole metabolic process
Erythroid homeostasis

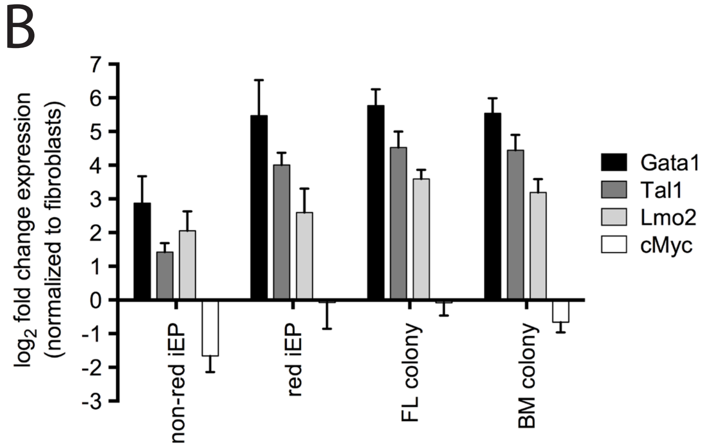

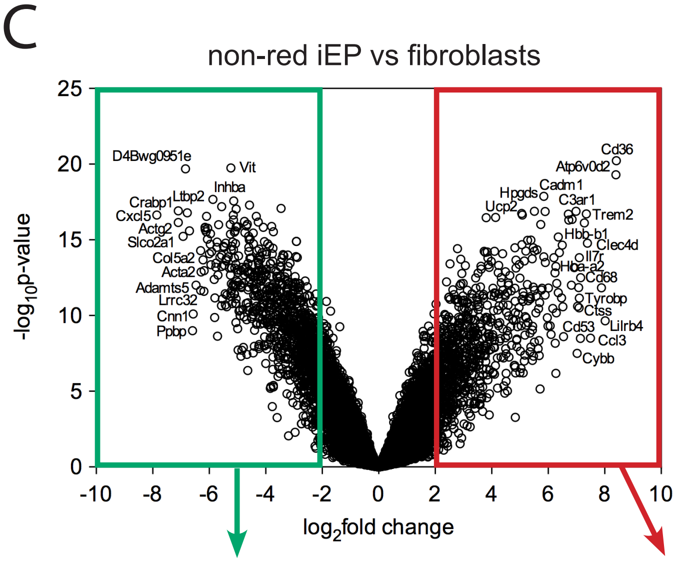

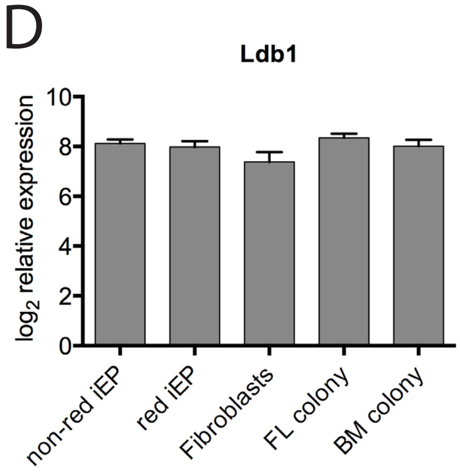

GO terms (1036 genes)
Cell adhesion
Biological adhesion
Vasculature development
Enzyme linked receptor protein signaling pathway
Skeletal system development
Blood vessel development
Cell motion
Transmembrane receptor protein tyrosine kinase signaling pathway
Extracellular structure organization
Chordate embryonic development
Embryonic development ending in birth or egg hatching
Skeletal system morphogenesis
Blood vessel morphogenesis
Cell migration
Tissue morphogenesis
Extracellular matrix organization
Embryonic morphogenesis
Cell morphogenesis involved in differentiation
Regulation of cell proliferation

GO terms (815 genes)
Immune response
Cell activation
Leukocyte activation
Positive regulation of immune system process
Response to wounding
Lymphocyte activation
Positive regulation of response to stimulus
Positive regulation of immune response
Inflammatory response
Defense response

Figure S4

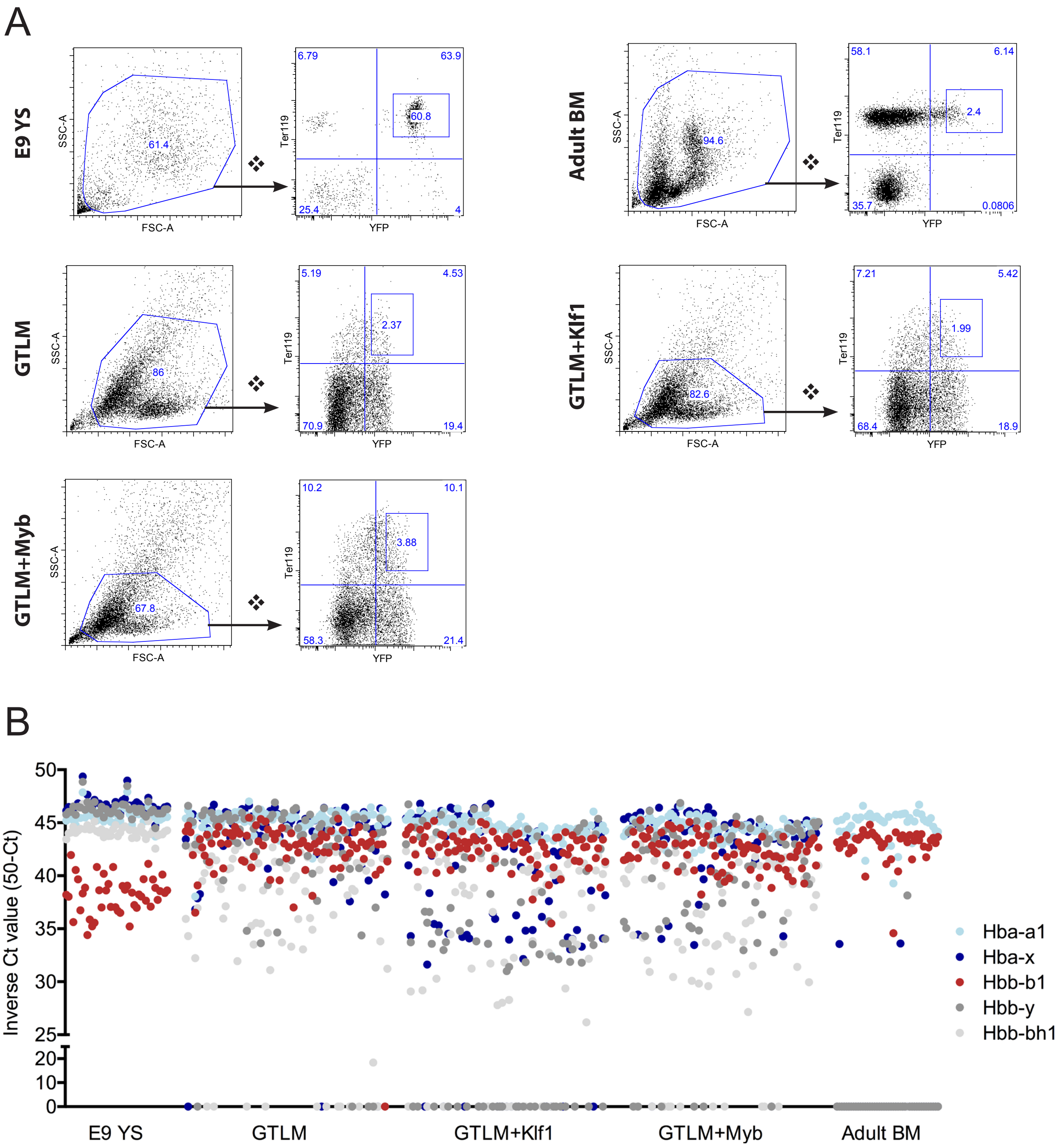
A
E9 YS
Adult BM
GTLM
GTLM+Klf1
GTLM+Myb
SSC-A
FSC-A
Ter119
YFP
61.4
6.79
63.9
60.8
25.4
4
94.6
58.1
6.14
2.4
35.7
0.0806
86
5.19
4.53
2.37
70.9
19.4
82.6
7.21
5.42
1.99
68.4
18.9
67.8
10.2
10.1
3.88
58.3
21.4
B
Inverse Ct value (50-Ct)
E9 YS
GTLM
GTLM+Klf1
GTLM+Myb
Adult BM
Hba-a1
Hba-x
Hbb-b1
Hbb-y
Hbb-bh1

Figure S5

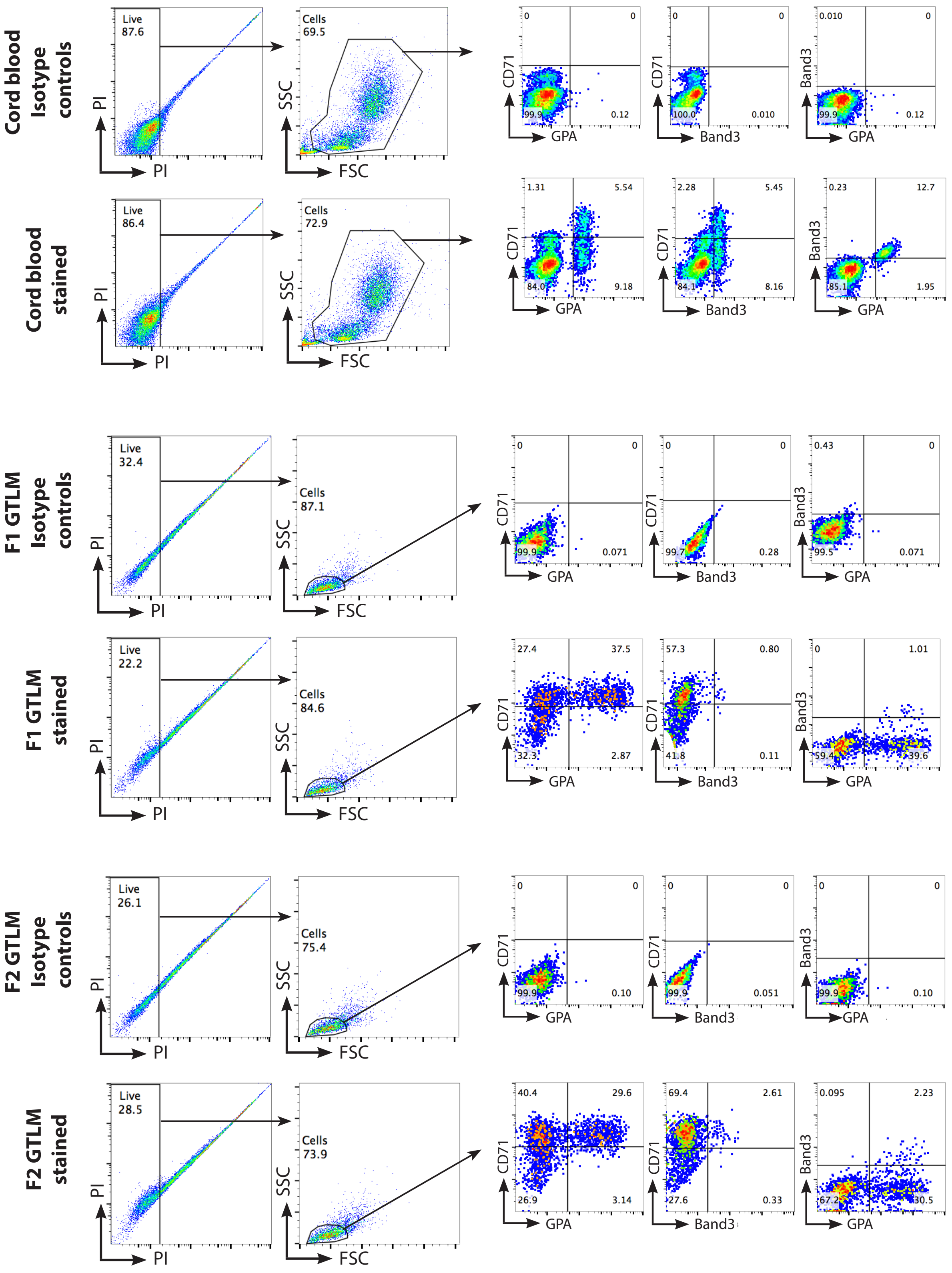
Cord blood Isotype controls
Cord blood stained
F1 GTLM Isotype controls
F1 GTLM stained
F2 GTLM Isotype controls
F2 GTLM stained
Live
87.6
86.4
32.4
22.2
26.1
28.5
Cells
69.5
72.9
87.1
84.6
75.4
73.9
PI
SSC
FSC
CD71
GPA
Band3
99.9
0.12
100.0
0.010
1.31
5.54
84.0
9.18
2.28
5.45
84.1
8.16
0.23
12.7
85.1
1.95
0.071
99.7
0.28
0.43
99.5
27.4
37.5
32.3
2.87
57.3
0.80
41.8
0.11
1.01
59.4
39.6
0.10
0.051
40.4
29.6
26.9
3.14
69.4
2.61
27.6
0.33
0.095
2.23
67.2
30.5

Figure S1, related to Figure 1. Forced Expression of *Gata1*, *Tal1*, *Lmo2* and *c-Myc* Reprograms Murine Adult Fibroblasts into Erythroid Progenitors

Representative live-cell bright field images of single wells after removal of individual TFs from the 7TF pool at day 8. Untransduced fibroblasts as negative control. Scale bar, 50 μm.

Figure S2, related to Figure 2. Induced Erythroid Progenitors Exhibit Properties of *Bona Fide* Erythroid Cells

(A) Time-course flow cytometry analysis of untransduced TTF (day 0) and bulk GTLM-transduced TTF harvested at day 2, 4, 6 and 8 showing YFP, CD45, CD71 and Ter119 expression, along with a graph displaying summary of data (n=3). Bone marrow from an *Epor*-Cre *R26*-eYFP adult mouse is shown as a staining control.

(B) Relative mRNA expression of *Gata1*, *Tal1*, *Lmo2* and *c-Myc* in untransduced TTF and bulk GTLM-transduced TTF harvested at day 5 and day 8, determined by qPCR. Primers were designed so that endogenous expression could be distinguished from total expression. Data are presented as mean ± SD (n=4-6 for iEPs, n=2 for untransduced TTF).

(C) Representative live-cell bright field, May-Grünwald Giemsa staining and Benzidin/Giemsa staining images of GTLM-iEPs generated from MEFs at day 8. Untransduced MEFs as negative control. Scale bar live-cell bright field, 50 μm. Scale bar cytospin, 20 μm. Bottom right, macroscopic inspection of cell pellets of GTLM-iEPs generated from MEFs and untransduced MEFs at day 8.

(D) Bright field and May-Grünwald Giemsa cytospin images of a representative red colony. D5-iEPs generated from MEFs were plated in semisolid media and

scored 8 days after. Scale bar live-cell bright field, 50 μm. Scale bar cytospin, 20 μm.

(E) Flow cytometry analysis of GTLM-iEPs derived from MEFs at day 8, gated on total live cells, showing CD71, Ter119 and CD45 expression.

Figure S3, related to Figure 3. iEP-Derived Red Colonies Retain a Primitive Erythroblast Expression Signature

(A) Volcano plot presenting differentially expressed genes between red iEP and non-red iEP. Genes with at least 4-fold higher expression in red iEP are highlighted with their most representative GO terms (pvalue$\leq 10^{-8}$) according to DAVID. Log_2fold difference is plotted on the *x* axis, and p-value-adjusted significance is plotted on the *y* axis ($-log_{10}$ scale).

(B) Log_2 fold change in expression of GTLM factors in non-red iEP, red iEP, FL colony and BM colony, relative to fibroblasts. Data are represented as mean ± SD (n=3).

(C) Volcano plot presenting differentially expressed genes between non-red iEP and fibroblasts. Genes with at least 4-fold higher expression in non-red iEP and genes with at least 4-fold more expression in fibroblasts are highlighted with their most representative GO terms (pvalue$\leq 10^{-8}$) according to DAVID. Log_2fold difference is plotted on the *x* axis, and p-value-adjusted significance is plotted on the *y* axis ($-log_{10}$ scale).

(D) Log_2 relative expression of Ldb1 determined by microarray. Data are represented as mean ± SD (n=3).

Figure S4, related to Figure 4. *Klf1* and *Myb* Enhance Adult Hemoglobin Expression in single iEPs

(A) Representative flow cytometry plots showing gating strategy used for index sorting of single cells for qRT-PCR. E9 yolk sac (E9 YS) was obtained by crossing an *Epor*-Cre *R26*-eYFP (*Epor* reporter) male with a WT female. Adult bone marrow was obtained from an *Epor* reporter mouse. GTLM-iEPs were obtained by reprogramming *Epor* reporter TTF, whereas GTLM+Klf1-iEPs and GTLM+Myb-iEPs were obtained by reprogramming *Epor* reporter TTF with constitutive expression of *Klf1* and *Myb*, respectively. ❖, doublets and dead-cell exclusion.

(B) Inverse Ct values of globin genes in single cells used to calculate the ranking in Figure 4B.

Figure S5, related to Figure 5. Induction of Erythroid Progenitors from Human Foreskin Fibroblasts by *Gata1*, *Tal1*, *Lmo2* and *c-Myc*

Representative flow cytometry plots showing gating strategy for cord blood mononuclear cells and transduced/untransduced human fibroblast samples.

Table S1, related to Figure 1 and Experimental Procedures “Molecular Cloning and production of retrovirus”. List of Genes Included in the Present Study and Corresponding Accession Numbers

Gene symbol	Gene ID	Species	Gene name
Bcl11a	NM_016707	Mus musculus	B-cell CLL/lymphoma 11A (zinc finger protein)
Bmi1	NM_007552	Mus musculus	Bmi1 polycomb ring finger oncogene
Cbfa2t3	NM_009824	Mus musculus	core-binding factor, runt domain, alpha subunit 2, translocated to, 3 (human)
Cbfb	NM_022309	Mus musculus	core binding factor beta
Cdx4	NM_007674	Mus musculus	caudal type homeo box 4
Cited2	NM_010828	Mus musculus	Cbp/p300-interacting transactivator, with Glu/Asp-rich carboxy-terminal domain, 2
E2F1	NM_007891	Mus musculus	E2F transcription factor 1
E2F2	NM_177733	Mus musculus	E2F transcription factor 2
E2F4	NM_148952	Mus musculus	E2F transcription factor 4
E2F8	NM_001013368	Mus musculus	E2F transcription factor 8
Egr1	NM_007913	Mus musculus	early growth response 1
Erg	NM_133659	Mus musculus	avian erythroblastosis virus E-26 (v-ets) oncogene related
Ets1	NM_011808	Mus musculus	E26 avian leukemia oncogene 1, 5' domain
Etv6	NM_007961	Mus musculus	ets variant gene 6 (TEL oncogene)
Fli1	NM_008026	Mus musculus	Friend leukemia integration 1
Zfpm1 (Fog1)	NM_009569	Mus musculus	zinc finger protein, multitype 1
Fos	NM_010234	Mus musculus	FBJ osteosarcoma oncogene
Foxo3a	NM_019740	Mus musculus	forkhead box O3
Gata1	NM_008089	Mus musculus	GATA binding protein 1
Gata2	NM_008090	Mus musculus	GATA binding protein 2
Gfi1b	NM_008114	Mus musculus	growth factor independent 1B
HoxA9	NM_010456	Mus musculus	homeo box A9
HoxB4	NM_010459	Mus musculus	homeo box B4
Ikzf1	NM_009578	Mus musculus	IKAROS family zinc finger 1
Irf2	NM_008391	Mus musculus	interferon regulatory factor 2
Klf1	NM_010635	Mus musculus	Kruppel-like factor 1 (erythroid)
Klf13	NM_021366	Mus musculus	Kruppel-like factor 13
Klf3	NM_008453	Mus musculus	Kruppel-like factor 3 (basic); similar to BKLF
Ldb1	NM_010697	Mus musculus	LIM domain binding 1
Lmo2	NM_008505	Mus musculus	LIM domain only 2
Lyl1	NM_008535	Mus musculus	lymphoblastomic leukemia 1
Mafk	NM_010757	Mus musculus	v-maf musculoaponeurotic fibrosarcoma oncogene family, protein K (avian)
Mef2c	NM_025282	Mus musculus	myocyte enhancer factor 2C
Meis1	NM_010789	Mus musculus	Meis homeobox 1

Mllt11	NM_019914	Mus musculus	myeloid/lymphoid or mixed-lineage leukemia (trithorax homolog, Drosophila); translocated to, 11
Myb	NM_010848	Mus musculus	myeloblastosis oncogene
Myc	NM_010849	Mus musculus	myelocytomatosis oncogene
Nfe2	NM_008685	Mus musculus	nuclear factor, erythroid derived 2
Nfkb1	NM_008689	Mus musculus	nuclear factor of kappa light polypeptide gene enhancer in B-cells 1, p105
Pou5f1 (Oct4)	NM_013633	Mus musculus	POU domain, class 5, transcription factor 1
Pbx1	NM_183355	Mus musculus	pre B-cell leukemia transcription factor 1; region containing RIKEN cDNA 2310056B04 gene; pre B-cell leukemia transcription factor 1
Runx1	NM_009821	Mus musculus	runt related transcription factor 1
Runx3	NM_019732	Mus musculus	runt related transcription factor 3
Sla	NM_009192	Mus musculus	src-like adaptor
Smarca4	NM_011417	Mus musculus	SWI/SNF related, matrix associated, actin dependent regulator of chromatin, subfamily a, member 4
Smarca5	NM_053124	Mus musculus	predicted gene 13034; SWI/SNF related, matrix associated, actin dependent regulator of chromatin, subfamily a, member 5
Sox12	NM_011438	Mus musculus	SRY-box containing gene 12
Sox6	NM_011445	Mus musculus	SRY-box containing gene 6
Sp3	NM_001098425	Mus musculus	trans-acting transcription factor 3
T (Brachyury)	NM_009309	Mus musculus	brachyury (T)
Tal1	NM_011527	Mus musculus	T-cell acute lymphocytic leukemia 1
Tf3 (E47)	NM_011548	Mus musculus	transcription factor 3
Tfdp1	NM_009361	Mus musculus	predicted gene 7390; transcription factor Dp 1; similar to Transcription factor Dp-1 (E2F dimerization partner 1) (DRTF1-polypeptide 1)
Tfdp2	NM_178667	Mus musculus	transcription factor Dp 2
Trib2	NM_144551	Mus musculus	tribbles homolog 2 (Drosophila)
Trim10	NM_011280	Mus musculus	tripartite motif-containing 10
Trim28	NM_011588	Mus musculus	tripartite motif-containing 28
Trim58	NM_001039047	Mus musculus	tripartite motif-containing 58
Zfp1	NM_011742	Mus musculus	zinc finger protein 1
Zfp143	NM_009281	Mus musculus	zinc finger protein 143
Zfp207	NM_011751	Mus musculus	zinc finger protein 207
Zfp219	NM_027248	Mus musculus	zinc finger protein 219
Zfp36l2	NM_001001806	Mus musculus	zinc finger protein 36, C3H type-like 2

Table S2, related to Figure 2, 4 and 5. List of the Taqman Assays Used for qPCR and single-cell qRT-PCR

Taqman assay ID	Gene	Taqman assay ID	Gene
Mm00836035_m1	Aff1	Mm00801891_m1	Nfe2
Mm00550729_m1	Ahctf1	Mm00433832_m1	Nr3c1
Mm00802083_m1	Alas2	Mm04207617_m1	Pbx1
Mm00657317_m1	Aldh1a1	Mm00479560_m1	Pbx2
Mm00508097_m1	Apq9	Mm00551055_m1	Pcgf6
Mm00431834_m1	Aqp1	Mm00482816_m1	Pdlim7
Mm01208559_m1	Aqp3	Mm00450111_m1	Postn
Mm01278161_m1	Aqp8	Mm00435969_m1	Prox1
Mm00492248_m1	Arid3a	Mm00456910_m1	RhD
Mm00479358_m1	Bcl11a	Mm00546741_m1	Rragd
Mm00487804_m1	c-Myc	Mm01213404_m1	Runx1
Mm00514283_s1	Cebpa	Mm03023996_m1	Smad4
Mm00516121_m1	Cited2	Mm00486320_s1	Sox4
Mm00801666_g1	Col1a1	Mm00488393_m1	Sox6
Mm00516347_m1	Crem	Mm01219775_m1	Stat3
Mm00484027_m1	Ctcf	Mm00839889_m1	Stat5b
Mm00624964_m1	E2f2	Mm01187033_m1	Tal1
Mm00514160_m1	E2f4	Mm00446971_m1	TBP
Mm00468171_m1	E2f5	Mm00443210_m1	Tcf4
Mm01329769_m1	Epb4.2	Mm00501505_m1	Tcf7l2
Mm00438760_m1	Epor	Mm00618407_m1	Tfdp2
Mm01175819_m1	Ets1	Mm00579691_m1	Thra
Mm00514851_m1	Foxh1	Mm01158806_m1	Zfp191
Mm01229605_g1	Foxn2	Mm00615562_m1	Zfp367
Mm01352636_m1	Gata1	Mm00494336_m1	Zfpm1
Mm00845395_s1	Hba-a1	Mm00523205_m1	Zgpat
Mm03039879_m1	Hba-x	Mm00518819_m1	Zxdc
Mm01611268_g1	Hbb-b1	Hs00163601_m1	ALAS2
Mm00433932_g1	Hbb-bh1	Hs00996794_m1	EPB42
Mm00433936_g1	Hbb-y	Hs00959427_m1	EPOR
Mm00468869_m1	Hif1a	Hs02758991_g1	GAPDH
Mm00469375_m1	Hif3a	Hs00361191_g1	HBA2/1
Mm00446968_m1	HPRT	Hs00758889_s1	HBB
Mm00516788_m1	Irf7	Hs00362216_m1	HBE1
Mm00492679_m1	Irf9	Hs00361131_g1	HBG2/1
Mm00516096_m1	Klf1	Hs00923579_m1	HBZ
Mm01254146_mH	Klf7	Hs00235006_m1	ITGA1
Mm01190673_m1	Lin28b	Hs00559595_m1	ITGB1
Mm01281680_m1	Lmo2	Hs00610592_m1	KLF1
Mm01318991_m1	Mef2a	Hs00920556_m1	MYB
Mm00841956_mH	Mrrf	Hs00958111_m1	VIM

Mm00501741_m1	Myb	Hs00419119_m1	ZFPM1

Table S3, related to Figure S2. List of Primers and Internal Oligos Used to Check Expression of Reprogramming Genes (see Figure S2B).

Gene	Primer	Sequence
Gata1 endogenous	F	agggagactcagaggccaag
	R	GCCCCTAGACCAGGAAAATC
	Internal oligo	aagcccaggttcaaccccag
Tal1 endogenous	F	Gtcctacctcgacccctctc
	R	CGTCCTGTCCCTCTAGTTGC
	Internal oligo	tatgccccaggATGACGGAG
Lmo2 endogenous	F	GTCCGAGTCCAGGCAGCTA
	R	CTCATCCACGGGTTCCTCAG
	Internal oligo	GCCAGCCACGCGCCACAAAGGG
c-Myc endogenous	F	tgaaggctggatttcctttg
	R	TTCTCTTCCTCGTCGCAGAT
	Internal oligo	cgATGCCCCTCAACGTGAAC
Tal1 total	F	GCCTCACTAGGCAGTGGGTT
	R	CCTCTTCACCCGGTTGTTGT
	Internal oligo	TGGGGAACCGGATGCCTTCCCCA
Lmo2 total	F	GGACGGAAATTGTGCAGGAG
	R	CTTTGTCTTTCACCCGCATC
	Internal oligo	TGTGACAAGCGGATCCGTGC

Gene	Company	ID
Gata1 total	IDT	Mm.PT.56a.10444529
c-Myc total	IDT	Mm.PT.56a.28494642
Actb	IDT	Mm.PT.58.33540333

Table S4, related to Experimental Procedures "Molecular Cloning and production of retrovirus". List of the Primers Used to Clone Constructs

cDNA (restriction) orientation	Primer sequence
Gata1 (BamHI) F	caaccGGATCCGCCACCATGGATTTTCCTGGTCTAGG
Gata1 (NotI) R	cacaagGCGGCCGCtattctgtgtacctTCAAGAAC
Klf1 (BamHI) F	TcctaGGATCCGCCACCATGAGGCAGAAGAGAGAGAGGAGGCCTG
Klf1 (NotI) R	cttgtGCGGCCGCacTCAGAGGTGACGCTTCATGTGCAGAGC
Lmo2 (BglII) F	ACGCCGAGATCTGCCACCATGTCCTCGGCCATCGAAAGG
Lmo2 (NotI) R	cctggGCGGCCGCCTAGATGATCCCATTGATCTTG
Myb (BamHI) F	gccccGGATCCGCCACCatggcccggagaccccgacacagc
Myb (NotI) R	cataatGCGGCCGCtggaaatgtctcacatgaccagagttc
c-Myc (BamHI) F	cgttGGATCCGCCACCATGCCCCTCAACGTGAACTTC
c-Myc (NotI) R	attccaGCGGCCGCgagttaggtcagtTTATGCACCAG
Nfe2 (BamHI) F	tcagctGGATCCGCCACCATGCCCCCGTGTCCTCCTCAG
Nfe2 (NotI) R	agcaccaGCGGCCGCtctctagaccagcTCAATCTGTAG
Sox6 (BglII) F	gaaggaaAGATCTgaaagagagaagaATGTCTTCCAAGC
Sox6 (NotI) R	agcaaacaGCGGCCGCTCAGTTGGCACTGACAGGCTCTG
Tal1 (BamHI) F	taaatatGGATCCGCCACCATGACGGAGAGGCCGCCGAGCGAGGC
Tal1 (NotI) R	tcagttggGCGGCCGCaaagttcaaactgtaagggaagttc

SUPPLEMENTAL EXPERIMENTAL PROCEDURES

Hematopoietic lineage depletion of mouse fibroblast cultures

Fibroblast cultures were trypsinized and re-suspended at $1x10^7$ cells/mL in PBS + 2% FCS. 10μg purified rat anti-mouse antibodies (CD45, Ter119, Gr-1, B220, CD4, CD5, CD11b and CD8a (Biolegend) and CD105 (SouthernBiotech)) were added per 10^7 cells and incubated for 10 min at 4ºC. Cells were washed and re-suspended in PBS + 2% FCS to $1x10^7$ cells/mL and mixed with 100ul Dynabeads sheep anti-rat IgG (Life Technologies). Cell suspension was incubated for 30 min at 4ºC with gentle rotation and then placed in contact with a magnet for 2 min. Supernatant containing the unbound cells was kept and cryopreserved for subsequent experiments.

Human fibroblast cultures

Two primary fibroblast lines were derived from human foreskin biopsies obtained with informed consent from patients. Fibroblasts were cultured in DMEM containing 10% FBS, 2 mM GlutaMAX (Invitrogen), 1% antibiotics (Invitrogen). All cell lines were maintained in an incubator (37°C, 5% CO_2) with media changes every second day.

Isolation of mononuclear cells from cord blood bags

Human Cord Blood bags were obtained from Banc de Sang i Teixits, Hospital Duran i Reynals, Barcelona. Mononuclear cells (MNC) were isolated from CB using Lympholyte-H (Cederlane, Ontario, CA) by density gradient centrifugation, red blood-lysis and two rounds of washing with PBS.

Cytospin and May-Grünwald Giemsa staining

Around 10,000 cells were centrifuged and resuspended in 50μL of PBS with 2% FCS. Cells were cytocentrifuged using Shandon cytospin 3 (Block Scientific, Inc., NY, USA) at 500g for 3 minutes to allow adherence to the slides. Working Giemsa solution was prepared by mixing 3mL of Giemsa stock solution (Histolabs, Gothenburg) to 60mL of distilled water. Air-dried slides were stained in May-Grünwald solution (Merck, Germany) for 5 mins, briefly rinsed with distilled water and transferred to working Giemsa solution for 10 min. The slides were finally washed in distilled water and allowed to dry before examined under the microscope.

Flow cytometry antibodies and dilutions

Anti-mouse CD71-Biotin, 1:500 (BD Biosciences), Streptavidin-Qdot605, 1:600 (Life Technologies), anti-mouse Ter119-APC, 1:400 (Biolegend), anti-mouse CD45.2-APC-Cy7, 1:200 (eBioscience), anti-human CD71-FITC, (dilution as recommended by manufacturer) (BD Biosciences), anti-human CD235a-APC, (dilution as recommended by manufacturer) (Immunostep) and anti-human Band3-PE, (dilution as recommended by manufacturer) (IBGRL Research Products).

Single cell qRT-qPCR and data analysis

Single YFP+ Ter119+ cells were sorted into 96-well PCR plates (Sarstedt) containing 4 μl of lysis buffer [0.4% of NP40 buffer (Sigma), 65μM of dNTP mix (Takara), 2.4mM DTT (Invitrogen), 0.5 U/μL RNAseOUT (Life

Technologies) in nuclease-free water] using the index-sorting function of the Diva software and according to the gating strategy shown in Figure S4A. Target-specific pre-amplification was performed using CellsDirect One-Step qRT-PCR Kit (Life Technologies). Pre-amplification master mix [6.25μL of 2X reaction buffer, 1μL of SuperScriptIII RT/Platinum Taq mix, 1.5μL of Taqman assays equal volume mix and 0.007μL of XenoRNA control (Ambion, Life Technologies)] was added to each well and reverse transcription followed by pre-amplification was performed in a Biorad T100 thermal cycler using the following program: 1h at 50ºC, 2 min at 95ºC and 25 cycles of 15 sec at 95ºC and 4 min at 60ºC. No-RT controls were treated the same way except that SuperscriptIII RT enzyme was substituted by Taq Polymerase (Life Technologies). Pre-amplified samples were diluted 1:5 and run on Fluidigm 96.96 arrays on a Biomark device (Fluidigm) together with the Taqman assays listed in Table S2 and reagents according to manufacturer's instructions. Positive controls (10 and 20 cells) were also included in each 96-well plates. Data analysis was first performed in Fluidigm Real-Time PCR Analysis software, and Ct thresholds were set automatically using the auto detector function. Reactions with Ct>40 were considered negative. This data was then analyzed using the Single Cell Expression Visualizer webtool (http://stemsysbio.bmc.lu.se/SCexV/) (Lang et al., 2015) to generate heatmaps and Principal Component Analysis plots. XenoRNA (Ambion, Life Technologies) was used as a loading control and normalization method. Cells displaying a Ct value >14 in the XenoRNA were excluded from the analysis, as well as control samples.
